# *syn*‐Selective Epoxidation of Chiral Terminal Allylic Alcohols with a Titanium Salalen Catalyst and Hydrogen Peroxide

**DOI:** 10.1002/anie.202201790

**Published:** 2022-05-09

**Authors:** Fabian Severin, Giovanni M. Fusi, Christina Wartmann, Jörg‐Martin Neudörfl, Albrecht Berkessel

**Affiliations:** ^1^ Department of Chemistry Organic Chemistry University of Cologne Greinstraße 4 50939 Cologne Germany; ^2^ Dipartimento di Scienza e Alta Tecnologia—DiSAT Università degli Studi dell'Insubria Via Valleggio 9 22100 Como Italy

**Keywords:** Allylic Alcohols, Asymmetric Epoxidation, Match/Mismatch Effect, Salalen Ligands, Titanium

## Abstract

In the Sharpless asymmetric epoxidation of chiral secondary allylic alcohols, one substrate enantiomer is predominantly converted to the *anti*‐epoxy alcohol. We herein report the first highly *syn*‐selective epoxidation of terminal allylic alcohols using a titanium salalen complex as catalyst, at room temperature, and aqueous hydrogen peroxide as oxidant. With enantiopure terminal allylic alcohols as substrates, the epoxy alcohols were obtained with up to 98 % yield and up to >99 : 1 dr (*syn*). Catalyst loadings as low as 1 mol % can be applied without eroding the *syn*‐diastereoselectivity. Modification of the allylic alcohol to an ether does not affect the diastereoselectivity either [>99 : 1 dr (*syn*)]. Inverting the catalyst configuration leads to the *anti*‐product, albeit at lower dr (ca. 20 : 1). The synthetic potential is demonstrated by a short, gram‐scale preparation of a tetrahydrofuran building block with three stereocenters, involving two titanium salalen catalyzed epoxidation steps.

## Graphical abstract

While the Sharpless asymmetric epoxidation/kinetic resolution provides access to *anti*‐configured terminal epoxy alcohols with high selectivity, no analogous *syn*‐selective epoxidation process has been available. We report that this “missing link” is provided by a readily available titanium salalen catalyst. Besides allylic alcohols, allylic ethers are epoxidized with extreme *syn*‐selectivity, at low catalyst loading, with aqueous H_2_O_2_ as oxidant.

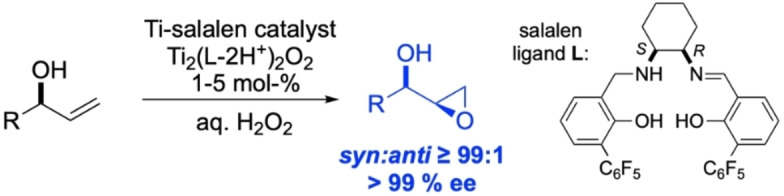

Chiral epoxides are generally valued as versatile building blocks in stereoselective synthesis.[^1^, ^2^] In this context, enantiopure epoxy alcohols, the epoxidation products of allylic alcohols, continue to play a particularly important role.^[3]^ Since its first disclosure in 1980, the Sharpless asymmetric epoxidation (AE) has served in countless cases for the highly enantioselective epoxidation of prochiral allylic alcohols (see Scheme 1a for an example).[^4^, ^5^, ^6^] Similarly, the Sharpless AE allows the highly efficient kinetic resolution (KR) of chiral allylic alcohols.^[7]^ In most cases, but not exclusively, chiral secondary allylic alcohols have served as substrates for the latter process, and a representative example is shown in Scheme 1b.[^8^, ^9^] Note that with the exception of *cis*‐2,3‐disubstituted allylic alcohols, the epoxide product is formed predominantly as the *anti*‐diastereomer. For terminal allylic alcohols such as the one shown in Scheme 1b, the *anti*‐preference is particularly pronounced (*anti*:*syn*=99 : 1).^[7]^ Similar *anti*‐preference was observed by Yamamoto et al. in the vanadium bis‐hydroxamic acid catalyzed KR of chiral secondary allylic alcohols.^[10]^ As a consequence of the *anti*‐selectivity, the preparation of enantiopure terminal *syn*‐epoxy alcohols for synthesis requires at least a two‐step process, such as Sharpless KR followed by Mitsunobu inversion of the epoxy alcohol configuration.^[11]^ For non‐enantioselective epoxidations, Adam et al.,^[12]^ and Scettri et al.^[13]^ have reported a pronounced influence of the secondary allylic alcohol structure and of the nature of the epoxidizing agent on the diastereoselectivity of the oxygen transfer. For terminal secondary allylic alcohol substrates, the highest *syn*:*anti* ratio achieved was 71 : 29 [stoichiometric Ti(O*i*Pr)_4_, *t*BuOOH].^[12]^ Enzymatic, monooxygenase‐catalyzed resolution by *syn*‐selective epoxidation has been reported for a few terminal secondary allylic alcohol substrates.^[14]^ Obviously, there is still a lack of general catalytic methodology for the selective preparation of enantiopure *syn*‐configurated terminal epoxy alcohols.

**Scheme 1:**
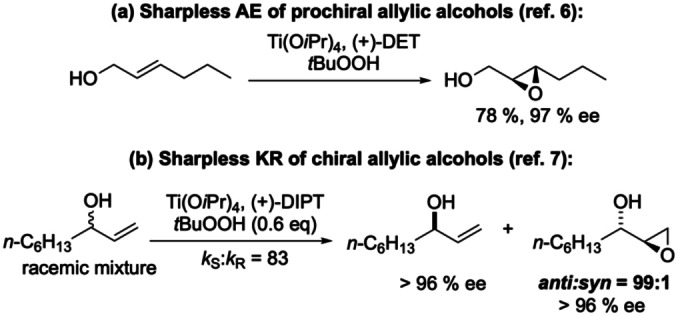
Titanium tartrate complex catalyzed epoxidation of allylic alcohols: Sharpless asymmetric epoxidation (a), and kinetic resolution (b).

We have reported earlier that the dimeric titanium complex **2**, derived from the *cis*‐DACH salalen ligand **1**, is a highly active and selective, yet readily available catalyst for the asymmetric epoxidation of *terminal* olefins, such as 1‐decene (Scheme 2a, b).[^15^, ^16^, ^17^, ^18^, ^19^, ^20^, ^21^] With aqueous hydrogen peroxide as oxidant, high yields of terminal epoxides with >95 % ee are routinely achieved. We could recently elucidate the intricate mechanism of this unique type of catalyst,^[22]^ and also shed light on the related importance of Ti‐dimers for the function of the industrial heterogeneous epoxidation catalyst TS‐1.^[23]^

We were curious to explore whether our Ti‐salalen catalyst **2** would maintain its high reactivity and stereoselectivity when applied to the epoxidation of chiral secondary allylic alcohols. As shown in Scheme 2c for undec‐1‐en‐3‐ol (**3a**), this approach could provide the “missing link” to terminal *syn*‐epoxy alcohols. As outlined in detail below, we were delighted to find that indeed the generation of *syn*‐epoxy alcohols represents the “matched case”^[24]^ in this system, resulting in products of extremely high diastereo‐ and enantiopurities (e.g. **4a**, Scheme 2c).

**Scheme 2:**
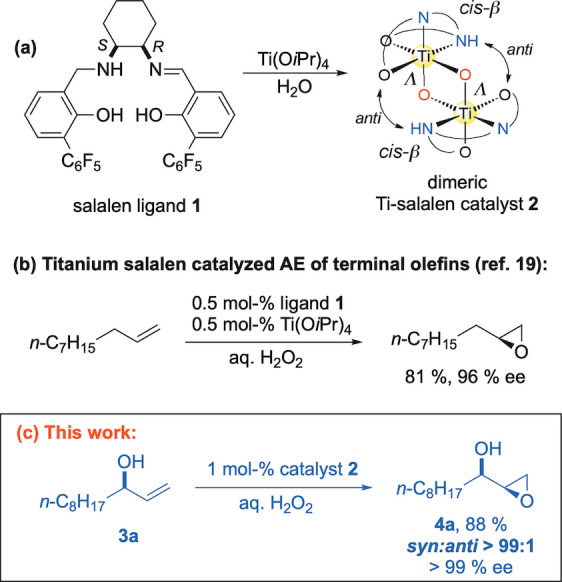
a) Structures of ligand **1** and Ti‐salalen catalyst **2** (schematic) developed by our group; b) asymmetric epoxidation of a terminal olefin with catalyst **2**; c) epoxidation of a terminal allylic alcohol with catalyst **2**.

We first surveyed the kinetic resolution of racemic undec‐1‐en‐3‐ol (*rac*‐**3a**) with the Ti‐salalen catalyst **2**, the results are summarized in Table 1. As may have been expected, catalyst **2** predominantly afforded the same epoxide configuration as for non‐functionalized terminal olefin substrates (compare e.g. with 1‐decene in Scheme 2a). Moreover, the (*R*)‐configurated allylic alcohol was predominantly converted, resulting in the hoped‐for *syn*‐selectivity of epoxy alcohol formation. However, while the enantiopurity of the *syn*‐epoxy alcohol product (3*R*)‐**4a** was in the high range (94–97 % ee) typical for catalyst **2**, the diastereoselectivity of *syn*‐epoxy alcohol formation was only moderate (ca. 8 : 1 in DCM as solvent; Table 1, entry 1; see below and Supporting Information for the determination of product configurations). In other words, the high enantioselectivity of catalyst **2** is maintained for terminal allylic alcohol substrates, while—in contrast to the Sharpless KR (Scheme 1b)—the configuration of the substrate allylic alcohol is of only moderate influence with regard to conversion rate.

**Table 1:** Kinetic resolution of racemic undec‐1‐en‐3‐ol (*rac*‐**3a**).^[a]^

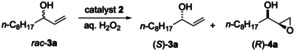				
Entry	Conversion	ee **3a** ^[d]^	Yield **4a**	*Syn:anti* **4a** ^[e]^
1^[b]^	42 %	42 %	32 %	7.9 : 1 (97 % ee *syn*)
2^[c]^	51 %	42 %	41 %	3.3 : 1 (94 % ee *syn*)

[a] 5 Mol‐% catalyst **2**, 0.5 equiv 50 % aq. H_2_O_2_, 20 °C, 48 h. Conversions, yields, ees and drs determined by GC on chiral stationary phase (see Supporting Information for analytical details). Predominant product configurations are shown in the Table head. [b] DCM as solvent. [c] Acetonitrile as solvent. [d] Configuration of major enantiomer: *S*. [e] Configuration at C‐3 of major diastereomer: *R*.

The above observation called for the use of enantiopure allylic alcohols as substrates. The data shown in Table 1 identify the combination of catalyst **2** (derived from the (1*R*,2*S*)‐ligand **1**, Scheme 2a) with the (*R*)‐configurated terminal allylic alcohol **3a** as the “matched pair”.^[24]^ Terminal allylic alcohols of this configuration^[25]^ can be obtained in large quantities and in virtually enantiopure form from the racemate by enzymatic kinetic resolution, using readily available *Candida antarctica* lipases (A+B).^[26]^ With this method, the four terminal allylic alcohols **3 a**–**c**, **e** shown in Table 2 were readily provided with >99 % ee, while **3d** of 98 % ee served as substrate (Table 2; see Supporting Information for experimental details).

**Table 2:** Epoxidation of the allylic alcohols **3a**–**e** with hydrogen peroxide, in the presence of the titanium salalen catalyst **2**.

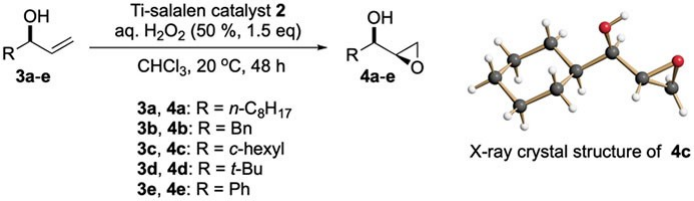					
Entry	Substrate/ product^[a]^	Catalyst **2** loading [mol‐%]	Yield **4 a**–**e** [%]^[b]^	*Syn:anti* **4 a**–**e** ^[b]^	ee **4 a**–**e** [%]^[b]^
1	**3 a**, **4 a**	5	91	>99 : 1	>99
2	**3 a**, **4 a**	1	88	>99 : 1	>99
3	**3 b**, **4 b**	5	86	>99 : 1	>99
4	**3 c**, **4 c**	5	93	>99 : 1	>99
5	**3 c**, **4 c**	1	70	>99 : 1	>99
6	**3 c**, **4 c** ^[c]^	1	88	>99 : 1	>99
7	**3 d**, **4 d**	10	97	98 : 2	>99
8	**3 d**, **4 d** ^[d]^	5	97	98 : 2	>99
9	**3 e**, **4 e**	5	98	>99 : 1	>99

[a] Enantiopurity **3a**–**c**, **e** >99 % ee, **3d**: 98 % ee. [b] Determined by GC on chiral stationary phase (see Supporting Information for analytical details). [c] Additional 1.5 equiv H_2_O_2_ after 24 h and 48 h, total reaction time 72 h. [d] Additional 1.5 equiv H_2_O_2_ after 24 h.

Exposure of the allylic alcohols **3a**–**e** to hydrogen peroxide and catalyst **2** resulted in smooth conversion to the pure *syn*‐epoxy alcohols **4a**–**e** in high yield (Table 2). Of the various solvents tried for this epoxidation, chloroform proved best (see Supporting Information for solvent screening). Inspection of Table 2 reveals that for all substrates **3a**–**e**, 5 mol‐% of catalyst were sufficient to achieve high product yields (entries 1, 3, 4, 8, 9). Note, however, that for the *tert*‐butyl allylic alcohol **3d**, either the catalyst loading had to be increased (entry 7), or addition of a second portion (1.5 equiv) of H_2_O_2_ was necessary to achieve full conversion (entry 8). For the *n*‐octyl allylic alcohol **3a** and the *c*‐hexyl substrate **3c**, the catalyst loading could even be reduced to 1 mol‐%, with no detectable loss of stereoselectivity, and without significant erosion of product yield (entries 2, 5, 6). However, to achieve full conversion of the *c*‐hexyl substrate **3c** at this low catalyst loading, the addition of two further portions of H_2_O_2_ was necessary (entry 6).

To further support the above “matched” assignment for the interaction of catalyst **2** with e.g. the substrate **3a**, we additionally subjected the allylic alcohol **3a** to epoxidation with the enantiomer of catalyst **2**, i.e*. ent*‐**2**. As shown in Scheme 3, top, indeed the *anti*‐diastereomer **5a** was obtained, but with an (expected) lower diastereoselectivity of 18 : 1 (*anti:syn*). In line with our mechanistic study on catalyst **2**—and in contrast to the Sharpless AE—there is no direct coordination of the allylic alcohol's hydroxyl group to the titanium ions in catalyst **2**.^[22]^ With this in mind, we set out to explore whether e.g. allylic ethers may also engage in the “matched” epoxidation process with catalyst **2**. To our delight, the methyl ether **6** derived from allylic alcohol **3c** gave the epoxy ether **7** with just the same excellent *syn*‐selectivity as the parent allylic alcohol (Scheme 3, bottom).

**Scheme 3:**
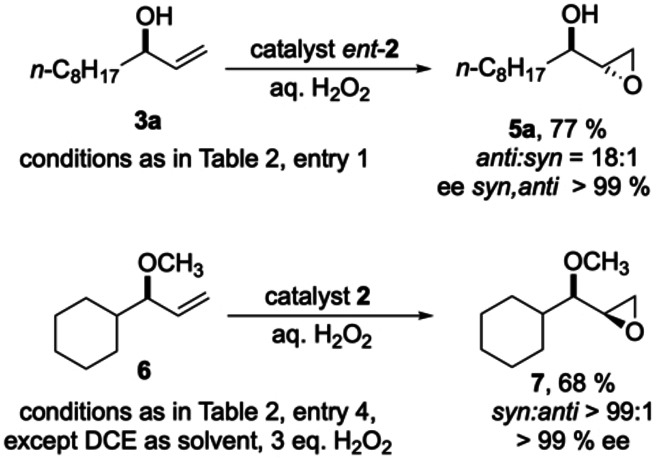
Top: *anti*‐selective epoxidation of the allylic alcohol **3a** with the “mismatched” catalyst *ent*‐**2**; bottom: *syn*‐selective epoxidation of the methyl ether **6** with the “matched” catalyst **2**.

The configurational assignments made in Tables 1,2 and Scheme 3 warrant further explanation. For all substrate allylic alcohols, the assignment of the enantiomer peaks in the chiral GC analysis rests on comparison with the pure enantiomers obtained from CAL‐A,B based kinetic resolution. For the latter, the absolute configuration for the remaining allylic alcohols **3 a**–**e** had been established before.[^27^, ^28^] For the assignment of the four product peaks, the enantiopure allylic alcohols were epoxidized with *m*CPBA to the corresponding *syn/anti* pairs. The *syn/anti* assignment of the resulting pairs was done based on typical shift patterns in their ^1^H NMR spectra (see Supporting Information for details).[^29^, ^30^] Additionally, the *syn*‐configuration and diastereomeric purity of the product epoxy alcohols is clearly documented by the NMR spectra of the isolated products (see Supporting Information for NMR spectra). Finally, we have been able to crystallize the epoxy alcohols resulting from the Ti‐salalen **2**‐catalyzed epoxidation of the allylic alcohol **3c** (**4c**, Table 2, R=*c*‐hexyl, *S*‐configuration) and from the (*R*)‐configurated pentadec‐1‐en‐3‐ol **3f** (**4f**, see Scheme 4 below).^[31]^ X‐Ray crystallography of these two samples (**4c** and **4f**) confirmed the (2*R*,3*R*)‐configuration in both cases (see Supporting Information for X‐ray data), and thus both the *syn*‐diastereoselectivity of the allyl alcohol epoxidation, and the enantiospecificity of the enzymatic resolution of the substrates.

**Scheme 4:**
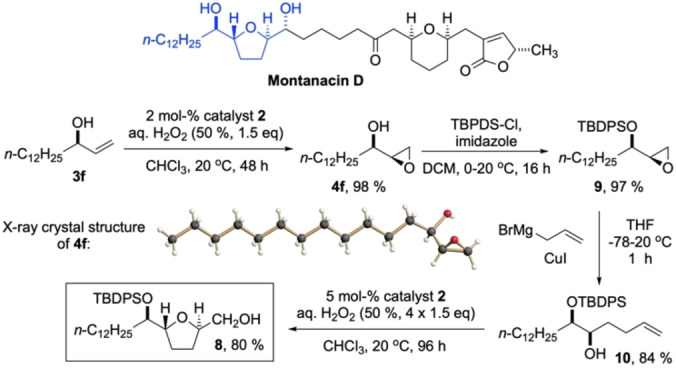
Top: Montanacin D and its *syn‐trans‐syn* tetrahydrofuran substructure (highlighted in blue); bottom: four‐step synthesis of the stereochemically uniform building block **8** (yields of isolated materials, 64 % total over four steps); X‐ray crystal structure of the enantiopure *syn*‐epoxy alcohol **4f**.

Finally, to demonstrate the synthetic potential of our *syn*‐selective epoxidation method, we chose as the target the 2,5‐bis(hydroxyalkyl)tetrahydrofuran subunit that is found e.g. in annonaceous acetogenins, such as the Montanacins.^[32]^ The molecular structure of Montanacin D, harboring a *syn‐trans‐syn* THF unit, is shown in Scheme 4, together with that of the envisaged *syn‐trans*‐THF building block **8**. As the starting material, we chose (*R*)‐pentadec‐1‐en‐3‐ol (**3f**), again available on large scale by CAL‐B resolution (see Supporting Information for details). Epoxidation with catalyst **2** afforded the pure *syn*‐epoxy alcohol **4f** in almost quantitative yield.^[33]^ After TBDPS‐protection of the hydroxyl group (97 %), copper(I)‐catalyzed epoxide opening in **9** with allyl magnesium bromide provided the terminal olefin **10** in 84 % yield. The epoxidation of the latter was again carried out with catalyst **2**. To our delight, cyclization of the intermediate (*S*)‐epoxide occurred spontaneously, and the *syn‐trans*‐tetrahydrofuran building block **8** was isolated in 80 % yield, on gram scale.^[34]^ Note that by proper choice of the allylic alcohol starting material, and of the epoxidation catalyst type (Sharpless or Berkessel‐Katsuki) and configuration, all stereocenters of e.g. compound **8** can be established at will.^[35]^

In summary, we have shown that the titanium salalen complex **2** (“Berkessel‐Katsuki catalyst”)^[20]^—developed previously for the highly enantioselective epoxidation of non‐functionalized terminal olefins—is an outstanding catalyst for the hitherto impossible *syn*‐selective epoxidation of terminal secondary allylic alcohols and ethers. *Syn*‐epoxidation occurs in the “matched pair” of this substrate–catalyst system, and *syn*‐selectivities≥99 : 1 were observed throughout. Nevertheless, *anti*‐epoxidation can be forced by switching to the “mismatched pair”, with *anti:syn* selectivity still in the range of ca. 20 : 1. Further advantages of the Ti‐salalen catalyst **2** are the low catalyst loading (1–2 mol % in most cases), and the use of aqueous hydrogen peroxide as oxidant.[^36^, ^37^] The practicality of this new tool for the catalytic preparation of stereochemically uniform *syn*‐epoxy alcohols was demonstrated by the short and efficient synthesis of a tetrahydrofuran building block for natural product synthesis.

## Conflict of interest

The authors declare no conflict of interest.

## Supporting information

As a service to our authors and readers, this journal provides supporting information supplied by the authors. Such materials are peer reviewed and may be re‐organized for online delivery, but are not copy‐edited or typeset. Technical support issues arising from supporting information (other than missing files) should be addressed to the authors.

Supporting Information

## Data Availability

The data that support the findings of this study are available in the Supporting Information of this article.
