# Supplementary material for: syn‐Selective Epoxidation of Chiral Terminal Allylic Alcohols with a Titanium Salalen Catalyst and Hydrogen Peroxide
Source: Angew Chem Int Ed Engl. 2022 May 9;61(26):e202201790. doi: 10.1002/anie.202201790 (PMC9325473; doi:10.1002/anie.202201790)
Supplement: Supplementary file 1 — Supporting Information [file ANIE-61-0-s001.pdf]

Angewandte Chemie
Eine Zeitschrift der Gesellschaft Deutscher Chemiker

Supporting Information

# *syn*-Selective Epoxidation of Chiral Terminal Allylic Alcohols with a Titanium Salalen Catalyst and Hydrogen Peroxide

*F. Severin, G. M. Fusi, C. Wartmann, J.-M. Neudörfl, A. Berkessel**

**Table of Contents**


1. Materials and Instrumentation 3
1.1. Materials 3
1.2. Instruments 3

2. Experimental Procedures 4
2.1. General procedure for the configurational assignment of the epoxy alcohols **4a**-**e** 4
2.2. General preparative procedures 4
2.3. Preparation of enantiopure allylic alcohols 4
2.4. Preparation of mixtures of the stereoisomers of the epoxy alcohol products 7
2.5. Catalytic epoxidations 9
2.5.1. Kinetic resolution of the allylic alcohol *rac*-**3a** by catalytic epoxidation 9
2.5.2. Optimization of the catalytic epoxidation of enantiopure undec-1-en-3-ol (**3a**): solvent screening 11
2.5.3. Optimization of the catalytic epoxidation of enantiopure undec-1-en-3-ol (**3a**): catalyst loading 11
2.5.4. Optimization of the catalytic epoxidation of enantiopure undec-1-en-3-ol (**3a**): additives 12
2.5.5. Catalytic epoxidation of the enantiopure allylic alcohols **3b**-**e** 12
2.5.6. Epoxidation of enantiopure undec-1-en-3-ol (**3a**) with the titanium salalen catalyst *ent*-**2** 14
2.5.7. Epoxidation of (*S*)-(1-methoxyallyl)cyclohexane (**6**) 14
2.6. Analytical data of hitherto unknown epoxide products 15
2.7. Synthesis of the THF-building block **8** 16

3. HPLC and GC-Methods 18
3.1. Allylic alcohols and related compounds 18
3.2. *syn/anti*-Epoxy alcohols obtained from *m*CPBA oxidation of racemic allylic alcohols, and *syn*-epoxy alcohols obtained from catalytic epoxidations with the titanium salalen catalyst **2** 25

4. NMR-Spectra 35
4.1. Allylic alcohols and related compounde 35
4.2. *syn/anti*-Epoxy alcohols obtained from *m*CPBA epoxidation 41
4.3. *syn*-Epoxy alcohols obtained by catalytic epoxidation with the titanium salalen catalyst **2** 44
4.4. THF Building block **8** and synthetic intermediates **9** and **10** 52

5. X-ray Crystallographic Data 56
5.1. X-Ray crystal structure of (*R*,*R*)-cyclohexyl(oxiran-2-yl)methanol (**4c**) 56
5.2. X-Ray crystal structure of (*R*,*R*)-1-(oxiran-2-yl)tridecan-1-ol (**4f**) 57

6. References 58

# 1. Materials and Instrumentation

## 1.1. Materials

All commercial reagents were used as received. *Candida antarctica* lipases A and B were bought in immobilized form from Merck Sigma-Aldrich. Anhydrous solvents were distilled and dried prior use over sodium or $CaH_2$. The Ti-catalysts **2** and *ent*-**2** were synthesized according to the literature.[1] The allylic alcohols were synthesized according to the literature procedure by *Breit* and *Grünanger*,[2] and the analytic data for undec-1-en-3-ol (*rac*-**3a**)[2], pentadec-1-en-3-ol (*rac*-**3f**)[3], 1-cyclohexylprop-2-en-1-ol (*rac*-**3c**)[4], 4,4-dimethylpent-1-en-3-ol (*rac*-**3d**)[4], and 1-phenylbut-3-en-2-ol (*rac*-**3b**)[4]) are in agreement with the literature. α-Vinylbenzyl alcohol (*rac*-**3e**) was bought from Merck Sigma-Aldrich. For the assignment of the configuration of the epoxy alcohols, they were synthesized from the racemic allylic alcohols by epoxidation with *m*CPBA, as well as from the enantiopure allylic alcohols, if necessary.

## 1.2. Instruments

Nuclear magnetic resonance (NMR) spectra were recorded on a Bruker Avance I 300, a Bruker Avance 499, or a Bruker Avance III 500 instrument in $CDCl_3$ at ambient temperature. Chemical shifts (δ) are reported in ppm and are relative to the tetramethylsilane (TMS) signal ($^1$H) or solvent signals ($^{13}$C). Coupling constants were reported in Hz with the following abbreviations for multiplicities: br = broad, s = singlet, d = doublet, t = triplet, q = quartet, m = multiplet. Infrared (IR) spectra were recorded on a Shimadazu IRAffinity-1 spectrometer with ATR technique. Wave numbers were reported in $cm^{-1}$ and the intensities of absorption bands are indicated by the following abbreviations: s = strong, m = medium, w = weak. GC-MS analysis was done on an Agilent Technologies 7890A instrument with injector and autosampler, and an Agilent Technologies 5975C Triple-Axis Detector. A HP-5 MS column (length: 30.0 m, inner diameter: 0.25 mm, film thickness: 0.25 µm) was used with $H_2$ as carries gas and the following temperature program 50 °C, 5 min, 20 °C/min to 280 °C, 10 min. HR-GC-MS analysis was done on a Thermo Scientific Exactive GC with an Orbitrap Analyser. Chiral HPLC analysis was done on a HITACHI-Chromaster system with pump (5160), autosampler (5260), column oven (5310) and detector (5430), and a Daicel Chiracel OD-H column (length: 25 cm, inner diameter: 4.6 mm). Chiral GC analysis was done on an Agilent Technologies 6890N instrument with injector 7683B, autosampler and a Chirasil-Dex CB column (length: 25.0 m, inner diameter: 250 µm, film thickness: 0.25 µm) and on an HP 6890 instrument with injector 6890, autosampler and a Lipodex A (length: 25.0 m, inner diameter: 250 µm, film thickness: 0.25 µm) or a Hydrodex β-3-P (length: 25.0 m, inner diameter: 250 µm, film thickness: 0.25 µm). Melting points were measured on an Apotec instrument of Kleinfeld Labortechnik, and are uncorrected. Elemental Analyses (EA) were performed on an Elementar Vario MICRO cube from Elementar Analysesysteme GmbH.

## 2. Experimental Procedures

### 2.1. General procedure for the configurational assignment of the epoxy alcohols 4a-e

First, the racemic allylic alcohols *rac*-**3a**-**e** were subjected to epoxidation with *m*CPBA (see 2.2, "general procedure B"), affording mixtures of diastereomeric racemates. These racemic mixtures were assigned as *syn* and *anti* by $^{1}$H NMR, according to *Mihelich* and *Sharpless et al.*[5] The NMR assignment of *syn/anti* to the major/minor racemates was then transferred to the GC- and HPLC-analyses. For the assignment of absolute configurations, the enantiomerically pure allylic alcohols **3a**-**e** were epoxidized.

### 2.2. General preparative procedures

**General procedure A: Enzymatic kinetic resolution of the allylic alcohols *rac*-3a-e**

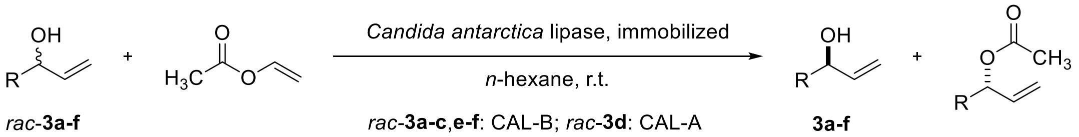


*rac*-**3a-f** *rac*-**3a-c**,**e-f**: CAL-B; *rac*-**3d**: CAL-A **3a-f**

The kinetic resolution was carried out according to the procedure of *Porto et al.*.[6] To a solution of the racemic allylic alcohol (3.77 mmol, 1.00 eq) in 30 mL *n*-hexane (HPLC grade), 120 mg of immobilized lipase was added. Vinyl acetate (37.8 mmol, 10.0 eq) was then added, and the reaction mixture was shaken at 150 rpm at room temperature in a stoppered 100 mL Erlenmeyer flask. The reaction was monitored via chiral GC. Upon complete consumption of one of the allylic alcohol substrates, the enzyme was filtered off and washed four times with 30 mL cyclohexane. The solvent was evaporated under reduced pressure, and the crude product was purified by flash column chromatography on silica.

**General procedure B: Epoxidation of allylic alcohols with *m*CPBA**

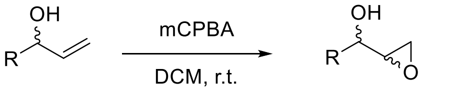


*rac*-**3a-f** *rac-syn/anti*-**4a-f**

To a solution of the racemic allylic alcohol (1.78 mmol, 1.00 eq) in 20 mL dichloromethane was added *m*CPBA (ca. 75% peracid) (500 mg, ca. 2.20 mmol, ca. 1.25 eq). When the TLC control indicated full consumption of the allylic alcohol, the reaction was quenched with 20 mL sat. aq. $Na_2S_2O_3$-solution or 20 mL sat. aq. $Na_2SO_3$-solution, and 20 mL sat. $NaHCO_3$-solution. The phases were separated, and the aqueous phase was extracted three times with 15 mL dichloromethane. The combined organic phases were dried over $MgSO_4$ or $Na_2SO_4$, filtered, and the solvent was evaporated under reduced pressure. The crude product was purified by flash column chromatography on silica.

### 2.3. Preparation of enantiopure allylic alcohols

**Kinetic resolution of racemic undec-1-en-3-ol (*rac*-3a)**

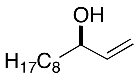


**3a**

The kinetic resolution was performed on a 3.77 mmol scale according to *general procedure A*.
The product (*R*)-undec-1-en-3-ol (**3a**) was obtained as a colorless liquid. Yield: 296 mg (1.74 mmol, 46%); $R_f$ = 0.26 ($SiO_2$, *c*Hex:EtOAc = 6:1); $^{1}$H-NMR (300 MHz, $CDCl_3$) δ [ppm] = 5.87 (ddd, *J* = 16.9, 10.4, 6.2 Hz; 1H), 5.22 (dt, *J* = 17.2, 1.5 Hz; 1H), 5.10 (dt, *J* = 10.4, 1.4 Hz; 1H), 4.10 (q, *J* = 6.4 Hz, 1H), 1.60 – 1.20 (m, 14H), 0.93 – 0.83 (m, 3H). Analytical data are in agreement with the literature.[2] The enantiomeric excess was determined to be >99% by chiral GC (Lipodex A; 93 °C isothermal 45 min, 10 °C/min to 130 °C, isothermal 30 min, 10 °C/min to 180 °C isothermal 5 min; flow 1.0 mL/min; $\tau_R$ = [minor, (*S*)-**3a**] = 39.2 min; [major, (*R*)-**3a**] = 40.3 min).

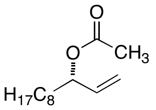

*ent*-**3a-Ac**

The product (*S*)-undec-1-en-3-yl acetate (*ent*-**3a-Ac**) was obtained as a colorless liquid. Yield: 400 mg (1.88 mmol, 50%); $R_f$ = 0.80 ($SiO_2$, cHex:EtOAc = 6:1); $^1$H-NMR (300 MHz, $CDCl_3$) δ [ppm] = 5.77 (ddd, *J* = 17.0, 10.5, 6.3 Hz; 1H), 5.32 – 5.11 (m, 3H), 2.06 (s; 3H), 1.73 – 1.48 (m; 2H), 1.40 – 1.16 (m; 12H), 0.94 – 0.81 (m; 3H). Analytical data are in agreement with the literature.[7] The enantiomeric excess was determined to be 94% after hydrolysis of the acetate to the alcohol *ent*-**3a** by chiral GC (Lipodex A; see analytical details above).

**Kinetic resolution of 1-phenylbut-3-en-2-ol (*rac*-3b)**

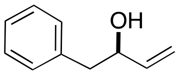

**3b**

The kinetic resolution was performed on a 1.09 mmol scale according to *general procedure A*.
The product (*R*)-1-phenylbut-3-en-2-ol (**3b**) was obtained as a colorless liquid. Yield: 37 mg (0.25 mmol, 23%); $R_f$ = 0.17 ($SiO_2$, pentane:$Et_2O$ = 6:1); $^1$H-NMR (300 MHz, $CDCl_3$) δ [ppm] = 7.36 – 7.28 (m; 2H), 7.28 – 7.20 (m; 3H), 5.94 (ddd, *J* = 17.2, 10.5, 5.8 Hz; 1H), 5.25 (dt, *J* = 17.2, 1.4 Hz; 1H), 5.13 (dt, *J* = 10.5, 1.4 Hz; 1H), 4.36 (dtd, *J* = 7.9, 5.6, 4.0 Hz; 1H), 2.89 (dd, *J* = 13.6, 5.2 Hz; 1H), 2.79 (dd, *J* = 13.6, 7.9 Hz; 1H), 1.61 (d, *J* = 4.0 Hz; 1H). Analytical data are in agreement with the literature.[4] The enantiomeric excess was determined to be >99% by chiral GC (Chiralsil-DEX CB; 100 °C isothermal 30 min, 10 °C/min to 180 °C isothermal 5 min; flow 2.0 mL/min; $\tau_R$ = [major (*R*)-**3b**] = 20.5 min; [minor (*S*)-**3b**] = 22.1 min).

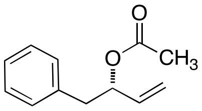

*ent*-**3b-Ac**

The product (*S*)-1-phenylbut-3-en-2-yl acetate (*ent*-**3b-Ac**) was obtained as a colorless liquid. Yield: 118 mg (620 µmol, 57%); $R_f$ = 0.61 ($SiO_2$, pentane:$Et_2O$ = 6:1); $^1$H-NMR (500 MHz, $CDCl_3$) δ [ppm] = 7.32 – 7.24 (m; 2H), 7.24 – 7.15 (m; 3H), 5.81 (ddd, *J* = 17.0, 10.5, 6.2 Hz; 1H), 5.51 – 5.42 (m; 1H), 5.20 (dt, *J* = 17.2, 1.3 Hz; 1H), 5.15 (dt, *J* = 10.5, 1.2 Hz; 1H), 2.96 (dd, *J* = 13.7, 7.4 Hz; 1H), 2.88 (dd, *J* = 13.7, 6.3 Hz; 1H), 2.01 (s; 3H). Analytic data are in agreement with the literature.[8] The enantiomeric excess was determined to be 45% by chiral GC (Chiralsil-DEX CB; 100 °C isothermal 30 min, 10 °C/min to 180 °C isothermal 5 min; flow 2.0 mL/min; $\tau_R$ = [minor (*R*)-**3b-Ac**] = 13.1 min; [major (*S*)-*ent*-**3b-Ac**] = 15.0 min).

**Kinetic resolution of racemic 1-cyclohexylprop-2-en-1-ol (*rac*-3c)**

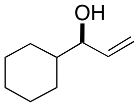

**3c**

The kinetic resolution was performed on a 1.00 mmol scale according to *general procedure A*.
The product (*S*)-1-cyclohexylprop-2-en-1-ol (**3c**) was obtained as a colorless liquid. Yield: 64 mg (0.46 mmol, 46%); $R_f$ = 0.20 ($SiO_2$, *c*Hex:EtOAc = 10:1); $^1$H-NMR (300 MHz, $CDCl_3$) δ [ppm] = 5.87 (ddd, *J* = 17.1, 10.4, 6.6 Hz; 1H), 5.20 (dt, *J* = 17.3, 1.5 Hz; 1H), 5.14 (dt, *J* = 10.4, 1.5 Hz; 1H), 3.85 (m; 1H), 1.91 – 1.60 (m; 5H), 1.49 – 1.33 (m; 2H), 1.32 – 0.91 (m; 5H). Analytical data are in agreement with the literature.[4] The enantiomeric excess was determined to be >99% by chiral GC (Chiralsil-DEX CB; 85 °C isothermal 45 min, 10 °C/min to 140 °C isothermal 20 min, 10 °C/min to 180 °C isothermal 5 min; flow 1.2 mL/min; $\tau_R$ = [minor (*R*)-**3c**] = 40.6 min; [major (*S*)-**3c**] = 42.2 min).

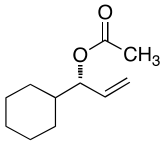

*ent*-**3c-Ac**

The product (*R*)-1-cyclohexylprop-2-en-1-yl acetate (*ent*-**3c-Ac**) was obtained as a colorless liquid. Yield: 85 mg (0.47 mmol, 46%); $R_f$ = 0.44 ($SiO_2$, cHex:EtOAc = 10:1); $^1$H-NMR (500 MHz, $CDCl_3$) δ [ppm] = 5.75 (ddd, *J* = 17.3, 10.5, 6.9 Hz; 1H), 5.24 – 5.15 (m; 2H), 5.04 (t, *J* = 6.8 Hz; 1H), 2.06 (s; 3H), 1.80 – 1.61 (m; 5H), 1.61 – 1.47 (m; 1H), 1.32 – 1.06 (m; 3H), 1.06 – 0.91 (m; 2H). Analytical data are in agreement with the literature.[9] The enantiomeric excess was determined to be >99% after hydrolysis of the acetate to the alcohol *ent*-**3c** by chiral GC (Chiralsil-DEX CB; see analytical details above).

**Kinetic resolution of racemic 4,4-dimethylpent-1-en-3-ol (*rac*-3d)**

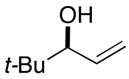

**3d**

The kinetic resolution was performed on a 1.75 mmol scale according to *general procedure A*.

The product (*S*)-4,4-dimethylpent-1-en-3-ol (**3d**) was obtained as a colorless liquid. Yield: 52 mg (0.47 mmol, 26%); $R_f$ = 0.39 ($SiO_2$, *n*Pentan:$Et_2O$ = 4:1); $^1$H-NMR (500 MHz, $CDCl_3$) δ [ppm] = 5.93 (ddd, *J* = 17.2, 10.5, 6.7 Hz; 1H), 5.26 – 5.20 (m; 1H), 5.20 – 5.14 (m; 1H), 3.75 (dt, *J* = 6.7, =1.1 Hz; 1H), 0.91 (s; 9H). Analytical data are in agreement with the literature.[4] The enantiomeric excess was determined to be 98% by chiral GC (Chiralsil-DEX CB; 50 °C isothermal 38 min, 10 °C/min to 140 °C isothermal 20 min, 10 °C/min to 180 °C isothermal 5 min; flow 1.5 mL/min; $\tau_R$ = [minor (*R*)-**3d**] = 29.7 min; [major (*S*)-**3d**] = 31.6 min).

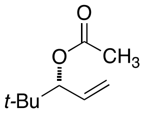

*ent*-**3d-Ac**

The product (*R*)-4,4-dimethylpent-1-en-3-yl acetate (*ent*-**3d-Ac**) was obtained as a colorless liquid. Yield: 171 mg (1.09 mmol, 63%); $R_f$ = 0.70 ($SiO_2$, *n*Pentan:$Et_2O$ = 4:1); $^1$H-NMR (500 MHz, $CDCl_3$) δ [ppm] = 5.85 – 5.73 (m; 1H), 5.25 – 5.21 (m; 1H), 5.21 – 5.17 (m; 1H), 5.00 – 4.96 (m; 1H), 2.08 (s; 3H), 0.92 (s; 9H). Analytical data are in agreement with the literature.[10] The enantiomeric excess was determined to be 60% after hydrolysis of the acetate to the alcohol *ent*-**3d** by chiral GC (Chiralsil-DEX CB; see analytical details above).

**Kinetic resolution of racemic α-vinylbenzyl alcohol (*rac*-3e)**

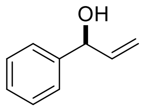

**3e**

The kinetic resolution was performed on a 11.2 mmol scale after *general procedure A*.

The product (*S*)-α-vinylbenzyl alcohol (**3e**) was obtained as a colorless liquid. Yield: 620 mg (4.62 mmol, 41%); $R_f$ = 0.20 ($SiO_2$, *c*Hex:EtOAc = 4:1); $^1$H-NMR (500 MHz, $CDCl_3$) δ [ppm] = 7.38 – 7.34 (m, 4H), 7.30 – 7.27 (m, 1 H), 6.10 – 6.01 (m, 1H), 5.35 (d, J = 17.8 Hz, 1H), 5.21 – 5.18 (m, 2H), 1.99 (d, J = 3.8 Hz, 1H); Analytic data are in agreement with the racemic mixture and literature.[6c] The enantiomeric excess was determined to be >99% by chiral GC (Chirasil-DEX CB; 40 °C isothermal 1 min, 2 °C/min to 130 °C, 20 °C/min to 170 °C isothermal 5 min; flow 1.0 mL/min; $\tau_R$ = [minor (*R*)-**3e**] = 42.4 min; [major (*S*)-**3e**] = 43.2 min).

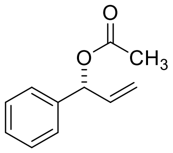

*ent*-**3e-Ac**

The product (*R*)-1-phenyl-2-propenyl acetate (*ent*-**3e-Ac**) was obtained as a colorless liquid. Yield: 880 mg (5.00 mmol, 45%); $R_f$ = 0.52 ($SiO_2$, cHex:EtOAc = 4:1); $^1$H-NMR (500 MHz, $CDCl_3$) δ [ppm] = 7.38 – 7.28 (m, 5H), 6.27 (dt, J = 5.8 Hz, 1.4 Hz, 1 H), 6.05 – 5.97 (m, 1H), 5.32 – 5.22 (m, 2H), 2.11 (s, 3H); Analytic data are in agreement with the literature.[6c] The enantiomeric excess was determined to be >99% by chiral GC (Chirasil-DEX CB; 40 °C isothermal 1 min, 2 °C/min to 130 °C, 20 °C/min to 170 °C isothermal 5 min; flow 1.0 mL/min; $\tau_R$ = [minor (*S*)-**3e-Ac**] = 34.6 min; [major (*R*)-**3e-Ac**] = 36.2 min).

**Kinetic resolution of racemic pentadec-1-en-3-ol (*rac*-3f)**

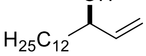

**3f**

The kinetic resolution was performed on a 11.7 mmol scale according to *general procedure A*.

The product (*R*)-pentadec-1-en-3-ol (**3f**) was obtained as a colorless solid, m.p. 33-35 ºC (m.p. ref.[11]: 28-30 ºC) Yield: 1.21 g (5.34 mmol, 46%); $R_f$ = 0.30 ($SiO_2$, *c*Hex:EtOAc = 6:1); $^1$H-NMR (300 MHz, $CDCl_3$) δ [ppm] = 5.86 (ddd, *J* = 16.9, 10.4, 6.2 Hz; 1H), 5.21 (dt, *J* = 17.2, 1.4 Hz; 1H), 5.09 (dt, *J* = 10.3, 1.2 Hz; 1H), 4.09 (qt, *J* = 6.3, 1.2 Hz; 1H), 1.59 – 1.45 (m; 2H), 1.45 – 1.14 (m; 21H), 0.87 (t, *J* = 6.7 Hz; 3H). Analytical data are in agreement with the literature.[3] The enantiomeric excess was determined to be >99% by chiral HPLC after derivatization with 3,5-dinitrobenzoyl chloride (Daicel Chiracel OD-H; *n*-hexane:isopropanol = 98:2; flow = 1.0 mL/min, 18 °C, fixed 210 nm; $\tau_R$ = [major (*R*)-**3f**] = 13.8 min; [minor (*S*)-**3f**] = 18.3 min).

**Preparation of (*S*)-(3-methoxyallyl)cyclohexane (6)**

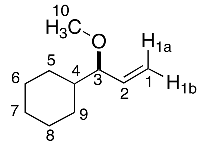


**6**

Under inert atmosphere, a solution of 140 mg (*S*)-1-cyclohexylprop-2-en-1-ol (**3c**, 1.00 mmol, 1.00 eq) in 2 mL THF was cooled to 0 °C. Sodium hydride [48 mg (60% in mineral oil), 2.00 mmol, 2.00 eq] was added, and the mixture was stirred at rt for 30 min. Methyl iodide (213 mg, 1.50 mmol, 1.50 eq) was then added, and the reaction mixture was stirred for 3 h. The reaction was quenched by addition of 10 mL sat. aq. $NH_4Cl$-solution. The aqueous phase was extracted with dichloromethane (3x10 mL). The combined organic phases were washed with brine, dried over $MgSO_4$, and filtered. The solvent was evaporated under reduced pressure, and the crude product was purified by flash column chromatography on silica gel (*c*Hex:DCM = 7:3 to 100% DCM). The product was obtained as a colorless liquid. Yield: 140 mg. (0.91 mmol, 91%); $R_f$ = 0.56 ($SiO_2$, *c*Hex:EtOAc = 10:1); $^1$H-NMR (500 MHz, $CDCl_3$) δ [ppm] = 5.63 (ddd, $^3J_{H2\text{-}H1a}$ = 17.3 Hz, $^3J_{H2\text{-}H1b}$ = 10.3 Hz, $^3J_{H2\text{-}H3}$ = 8.1 Hz; 1H, H2), 5.22 (dd, $^3J_{H1b\text{-}H2}$ = 10.3 Hz, $^2J_{H1b\text{-}H1a}$ = 1.9 Hz; 1H, H1b), 5.14 (ddd, $^3J_{H1a\text{-}H2}$ = 17.2 Hz, $^2J_{H1a\text{-}H1b}$ = 1.9 Hz, $^4J_{H1a\text{-}H3}$ = 0.7 Hz; 1H, H1a), 3.25 (s; 3H, H10), 3.23 – 3.19 (m; 1H, H3), 1.89 – 1.83 (m; 1H, H5a), 1.77 – 1.68 (m; 2H, H6a, H7), 1.68 – 1.59 (m; 2H, H8a, H9a), 1.48 – 1.38 (m; 1H, H4), 1.28 – 1.07 (m; 3H, H6b, H7b, H8b), 1.01 – 0.89 (m; 2H, H5b, H9); $^{13}$C-NMR (125 MHz, $CDCl_3$) δ [ppm] = 137.5 (1C, C2), 117.9 (1C, C1), 88.1 (1C, C3), 56.6 (1C, C10), 42.5 (1C, C4), 29.3 (1C, C5), 28.9 (1C, C9), 26.8 (1C, C8), 26.3 (1C, C6), 26.3 (1C, C7); IR (ATR): ṽ [cm$^{-1}$] = 3077 (w), 2979 (w), 2923 (s), 2853 (s), 2819 (w), 1642 (w), 1450 (s), 1420 (w), 1327 (w), 1263 (w), 1231 (w), 1195 (w), 1174 (w), 1117 (s), 1101 (s), 1086 (s), 995 (s), 971 (m), 922 (s), 888 (m), 885.2(w), 841.7 (w), 691.0 (w); GC-MS: $\tau_R$ = 8.3 min, m/z = 153.3 (M-H), 122.1 (M-$CH_4O$), 107.2, 94.1, 79.1, 71.1 (M-$C_6H_{11}$), 67.1, 55.1; HR-GC-MS: $\tau_R$ = 11.15 min, measured: m/z = 122.10891, theoretical: m/z 122.10955 (M-$CH_4O$); Analytical data are in agreement with the literature ($^1$H-NMR).[12]

## 2.4. Preparation of mixtures of the stereoisomers of the epoxy alcohol products

**Preparation of racemic *syn*/*anti*-1-(oxiran-2-yl)nonan-1-ol (*rac-syn/anti*-4a)**

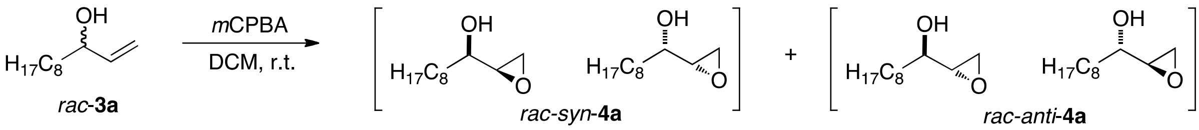


The epoxidation was performed on a 1.76 mmol scale according to *general procedure B*. The mixture of stereoiomeric products was obtained as a colorless liquid, with a ratio of the *syn*/*anti*-racemates of 56:44. Yield: 304 mg (1.63 mmol, 93%); $R_f$ = 0.13 ($SiO_2$, *c*Hex:EtOAc = 6:1).

***syn*-Epoxy alcohol *rac-syn*-4a**: $^1$H-NMR (300 MHz, $CDCl_3$) δ [ppm] = 3.44 (dq, *J* = 7.4, 5.7 Hz; 1H), 2.98 (td, *J* = 4.5, 2.7 Hz; 1H), 2.86 – 2.79 (m; 1H), 2.76 – 2.69 (m; 1H), 1.84 (dt, *J* = 6.0, 1.6 Hz; 1H), 1.67 – 1.20 (m; 14H), 0.88 (t, *J* = 6.9 Hz; 3H); Analytical data are in agreement with the literature.[13]

***anti*-Epoxy alcohol *rac-anti*-4a**: $^1$H-NMR (300 MHz, $CDCl_3$) δ [ppm] = 3.84 (ddt, *J* = 7.4, 4.9, 2.8 Hz; 1H), 3.02 (q, *J* = 3.3 Hz; 1H), 2.86 – 2.79 (m; 1H), 2.76 – 2.69 (m; 1H), 1.79 (d, *J* = 2.4 Hz; 1H), 1.67 – 1.20 (m; 14H), 0.88 (t, *J* = 6.9 Hz; 3H).

$^{13}$C-NMR (75 MHz, $CDCl_3$) δ [ppm] = 71.8 (1C, CH), 68.5 (1C, CH), 55.5 (1C, CH), 54.7 (1C, CH), 45.3 (1C, $CH_2$), 43.5 (1C, $CH_2$), 34.6 (1C, $CH_2$), 33.6 (1C, $CH_2$), 32.0 (2C, $CH_2$), 29.8 (2x1C, $CH_2$), 29.6 (2C, $CH_2$), 29.4 (2C, $CH_2$), 25.4 (2C, $CH_2$), 22.8 (2C, $CH_2$), 14.3 (2C, $CH_3$).

IR (ATR): ṽ [cm$^{-1}$] = 3439 (br), 2955 (m), 2922 (s), 2855 (s), 1458 (w), 1377 (w), 1254 (w), 1086 (m), 1057 (m), 974 (w), 910 (m), 851 (m), 721 (m); GC-MS: $\tau_R$ = 11.3 min, m/z = 152.2 (M–$H_2O_2$), 143.2 (M–$C_2H_3O$), 83.2, 69.1.

**Preparation of racemic *syn*/*anti*-1-(oxiran-2-yl)-2-phenylethan-1-ol (*rac-syn/anti*-4b)**

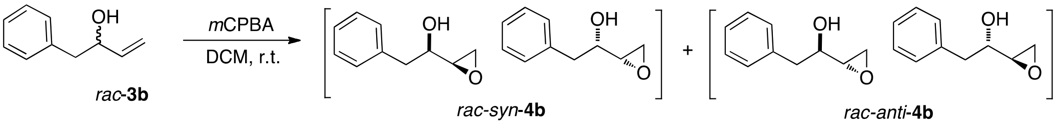


The epoxidation was performed on a 1.96 mmol scale according to *general procedure B*. The mixture of stereoisomeric products was obtained as a colorless liquid, with a ratio of the *syn*/*anti*-racemates of 59:41. Yield: 292 mg (1.78 mmol, 91%); $R_f$ = 0.17 ($SiO_2$, pentane:$Et_2O$ = 6:1).

***syn*-Epoxy alcohol *rac-syn*-4b**: $^1$H-NMR (300 MHz, $CDCl_3$) δ [ppm] = 7.36 – 7.28 (m; 2H), 7.28 – 7.21 (m; 3H), 3.72 (dtd, *J* = 7.3, 6.2, 4.6 Hz; 1H), 3.06 – 3.02 (m; 1H), 2.99 – 2.82 (m; 2H), 2.75 (dd, *J* = 4.9, 4.0 Hz; 1H), 2.62 (dd, *J* = 4.9, 2.7 Hz; 1H), f (m, 1H); $^{13}$C-NMR

(75 MHz, $CDCl_3$) δ [ppm] = 137.3 (1C, Cq), 129.5 (2C, $CH_2$), 128.7 (2C, $CH_2$), 126.8 (1C, CH), 72.4 (1C, CH), 54.7 (1C, CH), 45.2 (1C, $CH_2$), 41.2 (1C, $CH_2$); Analytical data are in agreement with the literature.[14]

***anti*-Epoxy alcohol *rac-anti*-4b**: $^1$H-NMR (300 MHz, $CDCl_3$) δ [ppm] = 7.36 – 7.28 (m; 2H), 7.28 – 7.21 (m; 3H), 4.01 (ddt, *J* = 7.9, 4.5, 2.9 Hz; 1H), 3.06 – 3.02 (m; 1H), 2.99 – 2.82 (m; 2H), 2.80 (dd, *J*= 5.0, 2.7 Hz; 1H), 2.75 (dd, *J* = 4.9, 4.0 Hz; 1H), 1.89 – 1.85 (m; 1H); $^{13}$C-NMR (75 MHz, $CDCl_3$) δ [ppm] = 137.3 (1C, Cq), 129.5 (2C, $CH_2$), 128.7 (2C, $CH_2$), 126.8 (1C, CH), 69.9 (1C, CH), 54.2 (1C, CH), 44.0 (1C, $CH_2$), 40.2 (1C, $CH_2$); Analytical data are in agreement with the literature.[14]

IR (ATR): ṽ [cm$^{-1}$] = 3424 (br), 3061 (w), 3026 (w), 2999 (w), 2922 (w), 1603 (w), 1495 (w), 1454 (w), 1254 (w), 1105 (w), 1080 (w), 1043 (w), 1032 (w), 989 (w), 926 (w), 912 (w), 885 (m), 870 (w), 849 (w), 831 (w), 746 (m), 698 (s); GC-MS: $\tau_R$ = 10.4 min, m/z = 164.1 (M), 146.1 (M–$H_2O$), 128.1, 118.1, 103.1, 92.1, 91.1 (M–$C_3H_5O_2$).

**Preparation of racemic *syn*/*anti*-cyclohexyl(oxiran-2-yl)methanol (*rac-syn/anti*-4c)**

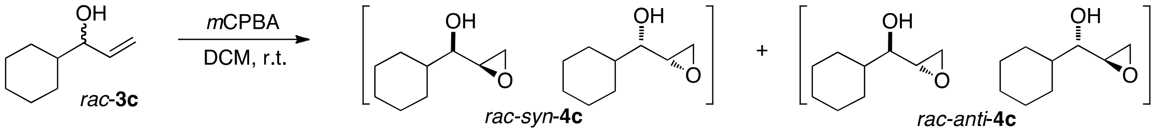


The epoxidation was performed on a 1.00 mmol scale according to *general procedure B*. The mixture of stereoisomeric products was obtained as a colorless liquid, with a ratio of the *syn*/*anti*-racemates of 57:43. Yield: 150 mg (0.96 mmol, 96%); $R_f$ = 0.19 ($SiO_2$, *c*Hex:EtOAc = 4:1).

**Mixture of the epoxy alcohols *rac-syn/anti*-4c**: $^1$H-NMR (500 MHz, $CDCl_3$) δ [ppm] = 3.66-3.60 (m; 1 H), 3.19 (dd, J = 11.7, 5.8 Hz; 1 H), 3.11 – 3.07 (m; 1 H), 3.07 – 3.02 (m; 1 H), 2.85 (dt, J = 5.3, 3.3 Hz; 2 H), 2.77 (dd, J = 5.0, 4.1 Hz; 1H), 2.72 (dd, J = 5.0, 2.8 Hz; 1 H), 1.98 – 1.63 (m; 10 H), 1.64 – 1.51 (m; 2 H), 1.35 – 1.01 (m; 10 H); Analytical data are in agreement with the literature.[15]

**Preparation of racemic *syn*/*anti*-2,2-dimethyl-1-(oxiran-2-yl)propan-1-ol (*rac-syn/anti*-4d)**

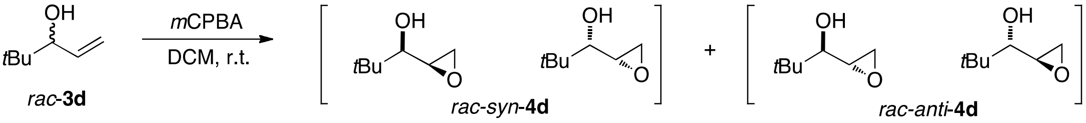


The epoxidation was performed on a 0.66 mmol scale according to *general procedure B*. The mixture of stereoisomeric products was obtained as a colorless liquid, with a ratio of the *syn*/*anti*-racemates of 48:52. Yield: 72 mg (0.56 mmol, 84%); $R_f$ = 0.23 ($SiO_2$, *c*Hex:EtOAc = 4:1).

**Mixture of the epoxy alcohols *rac-syn/anti*-4d:** $^1$H-NMR (300 MHz, $CDCl_3$) δ [ppm] = 3.52 (d, J = 2.9 Hz; 1H), 3.17 – 3.03 (m; 3H), 2.87 – 2.80 (m; 2H), 2.76 (dd, J = 5.1, 4.0 Hz; 1H), 2.70 (dd, J = 5.0, 2.6 Hz; 1H), 1.73 (brs; 2H), 1.00 (s; 18H).

**Preparation of racemic *syn-lanti*-1-(oxiran-2-yl)-phenyl methanol (*rac-syn/anti*-4e)**

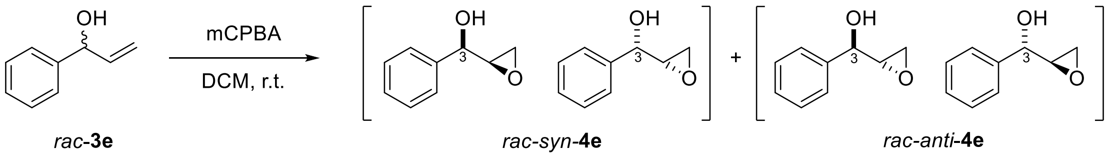


The epoxidation was performed on a 2.24 mmol scale after *general procedure B*. The mixture of stereoisomeric products was obtained as a colorless liquid, as a diastereomeric mixture with a ratio of the *syn*/*anti*-racemates of 1:1. Yield: 264 mg (1.89 mmol, 84%); $R_f$ = 0.13 ($SiO_2$, *c*Hex:EtOAc = 4:1).

**Mixture of the epoxy alcohols *rac-syn/anti*-4e**: $^1$H-NMR (300 MHz, $CDCl_3$) δ [ppm] =7.45 – 7.30 (m, 10 H), 4.94 (d, J = 2.5 Hz, 1H, H-$3_{anti}$), 4.48 (t, J = 4.7 Hz, 1H, H-$3_{syn}$), 3.25 – 3.21 (m, 2H), 2.97 (dd, J = 5.0, 2.8 Hz, 1H), 2.88 – 2.82 (m, 2H), 2.78 – 2.75 (m, 1H), 2.47 (d, J = 4.0 Hz, 1H), 2.29 (s, 1H); $^{13}$C-NMR (75 MHz, $CDCl_3$) δ [ppm] = 140.1 (1C, Cq), 139.4 (1C, Cq), 128.7 (1C, CH), 128.6 (1C, CH), 128.3 (1C, CH), 128.2 (1C, CH), 126.4 (1C, CH), 126.3 (1C, CH), 74.5 (1C, CH, C-$3_{syn}$), 70.8 (1C, CH, C-$3_{anti}$), 56.1 (1C, CH), 55.1 (1C, CH), 45.4 (1C, $CH_2$), 43.6 (1C; $CH_2$). Analytic data are in agreement with the literature.[16]

IR (ATR): ṽ [cm$^{-1}$] = 3435 (b), 3062 (w), 2997 (w), 1382 (w), 1254 (w), 1196 (w), 1040 (m), 1024 (m), 926 (m), 912 (m), 741 (s), 698 (s); GC-MS: $\tau_R$ = 10.4 min, m/z = 150.1 (M), 117.1, 107.1 (M-$C_2O$), 91.1, 79.1, 63.1, 51.1.

**Preparation of racemic *syn*/*anti*-1-(oxiran-2-yl)tridecan-1-ol (*rac-syn/anti*-4f)**

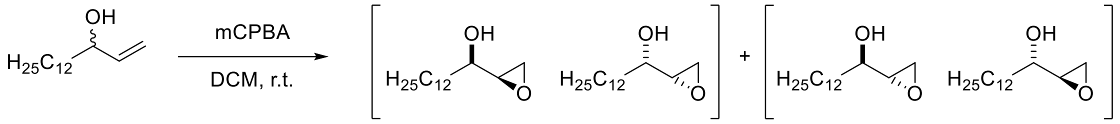

*rac*-**3f** *rac-syn*-**4f** *rac-anti*-**4f**

The epoxidation was performed on a 1.60 mmol scale according to *general procedure B*. The mixture of stereoisomeric products was obtained as a colorless liquid, with a ratio of the *syn*/*anti*-racemates of 56:44. Yield: 344 mg (1.42 mmol, 89%); $R_f$ = 0.26 ($SiO_2$, *c*Hex:EtOAc = 4:1).

***syn*-Epoxy alcohol *rac-syn*-4f**: $^1$H-NMR (300 MHz, $CDCl_3$) δ [ppm] = 3.48 – 3.38 (m; 1H), 2.98 (td, *J* = 4.5, 2.7 Hz; 1H), 2.84 – 2.79 (m; 1H), 2.75 – 2.71 (m; 1H), 1.90 – 1.84 (m; 1H), 1.66 – 1.19 (m; 22H), 0.88 (t, *J* = 6.9 Hz; 3H); Analytical data are in agreement with the literature.[3]

***anti*-Epoxy alcohol *rac-anti*-4f**: $^1$H-NMR (300 MHz, $CDCl_3$) δ [ppm] = 3.84 (ddt, *J* = 7.4, 4.9, 2.7 Hz; 1H), 3.02 (q, *J* = 3.3 Hz; 1H), 2.84 – 2.79 (m; 1H), 2.75 – 2.71 (m; 1H), 1.83 – 1.79 (m; 1H), 1.66 – 1.19 (m; 22H), 0.88 (t, *J* = 6.9 Hz; 3H); Analytical data are in agreement with the literature.[3]

$^{13}$C-NMR (75 MHz, $CDCl_3$) δ [ppm] = 71.8 (1C, CH), 68.5 (1C, CH), 55.5 (1C, CH), 54.7 (1C, CH), 45.3 (1C, $CH_2$), 43.5 (1C, $CH_2$), 34.6 (1C, $CH_2$), 33.6 (1C, $CH_2$), 32.1 (2C, $CH_2$), 29.8 (10C, $CH_2$), 29.7 (2C, $CH_2$), 29.5 (2C, $CH_2$), 25.4 (2C, $CH_2$), 22.8 (2C, $CH_2$), 14.3 (2C, $CH_3$).

IR (ATR): $\tilde{\nu}$ [$cm^{-1}$] = 3339 (br), 2955 (w), 2914 (s), 2874 (w), 2849 (s), 1466 (w), 1256 (w), 1111 (w), 1072 (w), 966 (w), 935 (w), 901 (w), 878 (w), 854 (m), 754 (w), 719 (m), 662 (w); GC-MS: $\tau_R$ = 13.7 min, m/z = 208.3 (M–$H_2O_2$), 199.2 (M–$C_2H_3O$), 125.0, 111.1, 97.2, 83.1.

**Preparation of racemic *syn*/*anti*-2-(cyclohexyl(methoxy)methyl)oxirane (*syn/anti-rac*-7)**

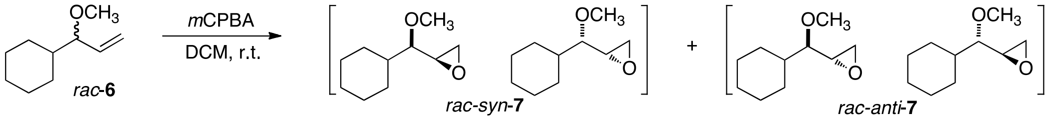

*rac*-**6** *rac-syn*-**7** *rac-anti*-**7**

The epoxidation was performed on a 1.24 mmol scale according to *general procedure B*. The mixture of stereoisomeric products was obtained as a colorless liquid, with a ratio of the *syn*/*anti*-racemates of 42:58. Yield: 208 mg (1.23 mmol, 98%); $R_f$ = 0.25 ($SiO_2$, *c*Hex:EtOAc = 10:1).

**Mixture of the epoxy alcohols *rac-syn/anti*-7:** $^1$H-NMR (300 MHz, $CDCl_3$) δ [ppm] = 3.47 (s; 3H), 3.38 (s; 3H), 3.00 – 2.92 (m; 2H), 2.82 –2.72 (m; 4H), 2.57 – 2.51 (m; 1H), 2.51 – 2.46 (m; 1H), 1.92 – 1.52 (m; 12H), 1.32 – 1.00 (m; 10H).

## 2.5. Catalytic Epoxidations

**General procedure for catalytic epoxidations**

A 5 mL reaction tube with a stirring bar (10 mm x 5 mm) was charged with the allylic alcohol (100 µmol, 1.00 eq), diphenyl ether (100 µmol, 1.00 eq, internal standard), 7.2 mg titanium salalen catalyst **2** (5 µmol, 0.05 eq) and 0.5 mL solvent. No inert atmosphere was applied. The solution was thermostated to 20 °C and stirred at 400 rpm. After 15 min, an aliquot (10 µL) was withdrawn with an Eppendorf pipette, passed through cotton/$MgSO_4$:$MnO_2$ (in a Pasteur pipette), and eluted with ethyl acetate. The epoxidation was then started by the addition of aqueous $H_2O_2$ (8.5 µL, 150 µmol, 1.50 eq, 50 % w/w in water) with an Eppendorf pipette. Aliquots were withdrawn in regular intervals, treated like the $t_0$-sample, and analyzed by chiral GC. For the NMR-characterization of the product epoxy alcohols **4a** to **4e**, the reaction mixtures were filtered through a small pad of $MgSO_4$:$MnO_2$. Solvents were removed from the filtrate under reduced pressure, and the residue was purified by flash column chromatography on silica.

*2.5.1. Kinetic resolution of the allylic alcohol rac-**3a** by catalytic epoxidation*

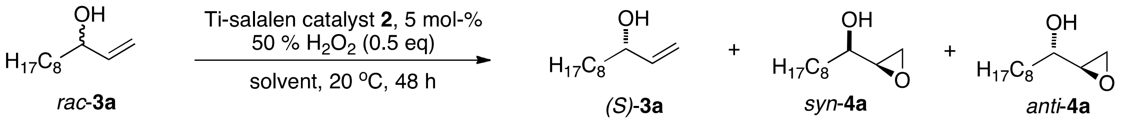


This kinetic resolution of racemic undec-1-en-3-ol (*rac*-**3a**) was performed on a 100 µmol scale according to the *general procedure for catalytic epoxidations* using 2.8 µL $H_2O_2$ (50 µmol, 0.50 eq). The aliquots withdrawn were analyzed by chiral GC (Lipodex A; 93 °C isothermal 45 min, 10 °C/min to 130 °C, isothermal 30 min, 10 °C/min to 180 °C, isothermal 5 min; flow 1.0 mL/min; $\tau_R$ = [major (*S*)-**3a**] = 39.2 min; [minor (*R*)-**3a**] = 40.3 min; ($Ph_2O$) = 49.9 min; [minor *syn*-(*S*,*S*)-**4a**] = 59.5; [major *anti*-(*R*,*S*)-**4a**] = 59.9 min; [minor *anti*-(*S*,*R*)-**4a**] = 60.5 min; [major *syn*-(*R*,*R*)-**4a**] = 61.3) min.

**Table S1.** Kinetic resolution of racemic undec-1-en-3-ol (*rac*-**3a**).

| Entry | solvent | time | conversion allylic alcohol[a] | *ee* allylic alcohol[a] | yield epoxy alcohol[a] | *ee* epoxy alcohol[a,b] | *dr syn:anti* epoxy alcohol[a] |
|---|---|---|---|---|---|---|---|
| 1 | DCM | 2 h | 0% | 1% | 1% | >99% | n.d. |
| | | 4 h | 7% | 7% | 7% | >99% | 9.8:1 |
| | | 20 h | 40% | 39% | 30% | 96% | 7.8:1 |
| | | 24 h | 40% | 41% | 30% | 97% | 8.0:1 |
| | | 48 h | 41% | 42% | 32% | 97% | 7.9:1 |
| 2 | MeCN | 2 h | 7% | 0% | 0% | n.d. | n.d. |
| | | 4 h | 9% | 0% | 1% | >99% | 4.4:1 |
| | | 20 h | 26% | 14% | 18% | 93% | 4.1:1 |
| | | 24 h | 31% | 18% | 20% | 96% | 4.3:1 |
| | | 48 h | 51% | 42% | 41% | 94% | 3.3:1 |

[a] Determined by GC on chiral stationary phase. [b] Enantiomeric excess of major diastereomer, *i.e. syn*-epoxy alcohol.

### 2.5.2. *Optimization of the catalytic epoxidation of enantiopure undec-1-en-3-ol (**3a**): solvent screening*

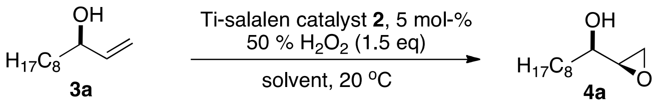


The catalytic epoxidation was performed on a 100 µmol scale, using the enantiopure undec-1-en-3-ol (*R*)-**3a** as the substrate. According to the *general procedure for catalytic epoxidations*, 8.5 µL $H_2O_2$ (150 µmol, 1.50 eq) in different solvents were employed. Aliquots were analyzed by chiral GC (Figure S1).

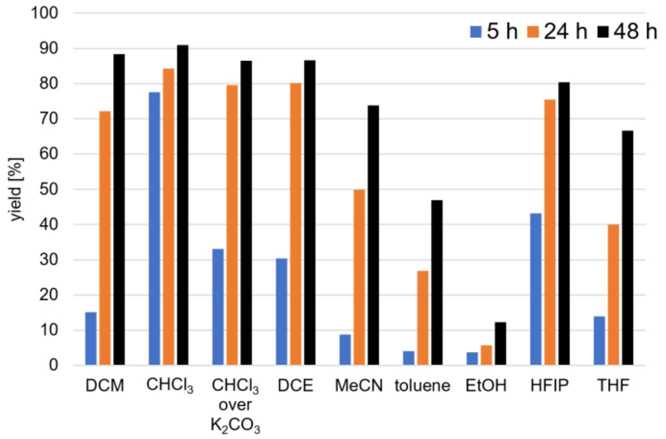


**Figure S1.** Yields of the epoxide *(R)*-**4a** in various solvents; dr >99:1 in all cases, except in EtOH as solvent (dr 30:1 after 48 h).

### 2.5.3. *Optimization of the catalytic epoxidation of enantiopure undec-1-en-3-ol (**3a**): catalyst loading*

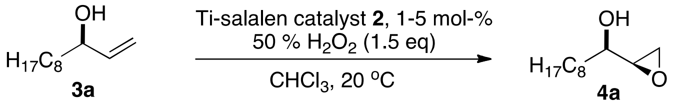


The catalytic epoxidation was performed on a 100 µmol scale, using the enantiopure undec-1-en-3-ol (*R*)-**3a** as the substrate. According to the *general procedure for catalytic epoxidations*, 8.5 µL $H_2O_2$ (150 µmol, 1.50 eq) were employed in chloroform as solvent, at different catalyst loadings (1-5 mol-%). Aliquots were analyzed by chiral GC (Figure S2).

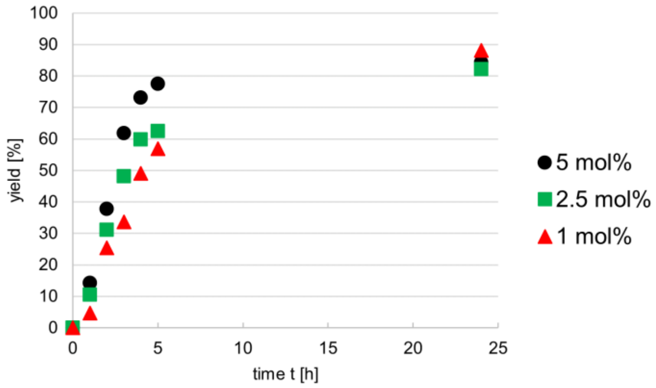


**Figure S2.** Yields of the epoxide **4a** at different catalyst loadings; dr > 99:1 in all cases; full conversion after 24 h for all three catalyst loadings.

*2.5.4. Optimization of the catalytic epoxidation of enantiopure undec-1-en-3-ol (**3a**): additives*

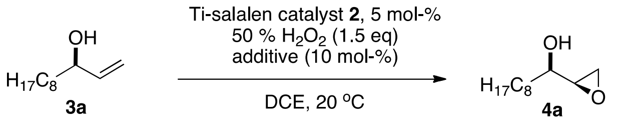


The catalytic epoxidation was performed on a 100 µmol scale, using the enantiopure undec-1-en-3-ol (*R*)-**3a** as the substrate. According to the *general procedure for catalytic epoxidations*, 8.5 µL $H_2O_2$ (150 µmol, 1.50 eq) were employed. DCE was used as solvent, with and without the additives PFBA (pentafluorobenzoic acid) or DTBP (2,6-di-*tert*-butyl pyridine) being added (10 µmol, 0.10 eq). Aliquots were analyzed by chiral GC (Figure S3).

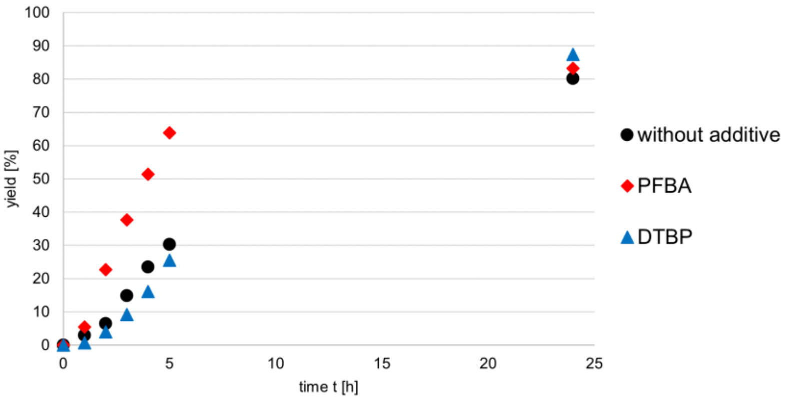


**Figure S3.** Yields of the epoxide **4a** in the presence/absence of additives; dr > 99:1 in all cases; full conversion after 24 h in all cases.

*2.5.5. Catalytic epoxidation of the enantiopure allylic alcohols **3a**-**e***

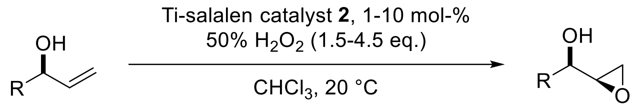


**3a-e** **4a-e**

According to the *general procedure for catalytic epoxidations*, reactions were performed on a 100 µmol scale, using the enantiopure allylic alcohols **3a**-**e** as the substrate. Chloroform was used as solvent.

**Table S2.** Summary of catalytic epoxidation of different allylic alcohols.

| Entry | Substrate, R = | Ti-catalyst (mol %) | $H_2O_2$ (eq) | Epoxide yield [%][a] (reaction time) | *ee*[a] | *dr*[a,c] |
|---|---|---|---|---|---|---|
| 1 | **3a**, R = $n$-$C_8H_{17}$ | 5 | 1.5 | 91 (48 h) | >99% | >99:1 |
| 2 | **3a**, R = $n$-$C_8H_{17}$ | 2.5 | 1.5 | 82 (24 h) | >99% | >99:1 |
| 3 | **3a**, R = $n$-$C_8H_{17}$ | 1 | 1.5 | 88 (48 h) | >99% | >99:1 |
| 4 | **3b**, R = Bn | 5 | 1.5 | 86 (48 h) | >99% | >99:1 |
| 5 | **3c**, R = $c$-$C_6H_{11}$ | 5 | 1.5 | 93 (48 h) | >99% | >99:1 |
| 6 | **3c**, R = $c$-$C_6H_{11}$ | 1 | 1.5 | 70 (48 h) | >99% | >99:1 |
| 7 | **3c**, R = $c$-$C_6H_{11}$ | 1 | 4.5[b] | 85 (48 h)<br>88 (72 h) | >99% | >99:1 |
| 8 | **3d**, R = $t$-$C_4H_9$[d] | 5 | 1.5 | 85 (48 h) | >99% | 98:2 |
| 9 | **3d**, R = $t$-$C_4H_9$[d] | 5 | 3[b] | 97 (48 h) | >99% | 98:2 |
| 10 | **3d**, R = $t$-$C_4H_9$[d] | 10 | 1.5 | 97 (48 h)<br>99 (72 h) | >99% | 98:2 |
| 11 | **3e,** R = Ph | 5 | 1.5 | 90 (24 h)<br>98 (48 h) | >99% | >99:1 |

[a] Determined by GC on chiral stationary phase. [b] 1.5 eq $H_2O_2$ every 24 h. [c] *syn:anti* [d] The allylic alcohol **3d** used as substrate had 98 % *ee*.

*2.5.6. Epoxidation of enantiopure undec-1-en-3-ol (**3a**) with the titanium salalen catalyst ent-**2***

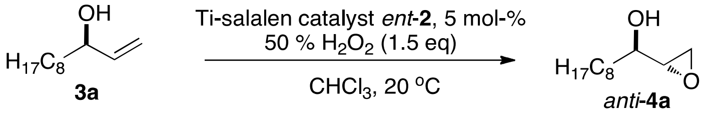


A reaction tube with a stirring bar (10 mm x 5 mm) was charged with the salalen ligand *ent*-**1** (6.5 mg, 10 µmol, 0.10 eq) and transferred into a glovebox. Ti(O*i*Pr)$_4$ (2.8 mg, 10 µmol, 0.10 eq) was added, together with 0.5 mL DCM. After 2 h, all volatiles were removed under reduced pressure. Then, 16.9 mg (*R*)-undec-1-en-3-ol (**3a**, 100 µmol, 1.00 eq), 16.1 mg diphenyl ether (95 µmol, 0.95 eq) and 0.5 mL $CHCl_3$ were added. The solution was thermostated to 20 °C and stirred at 400 rpm (no inert atmosphere applied). After 15 min, an aliquot (10 µL) was withdrawn with an Eppendorf pipette, passed through cotton/$MgSO_4$:$MnO_2$ (in a Pasteur pipette), and eluted with ethyl acetate. The epoxidation was then started by the addition of aqueous $H_2O_2$ (8.5 µL, 150µmol, 1.50 eq, 50 % w/w in water) with an Eppendorf pipette. Aliquots were withdrawn in regular intervals, treated like the $t_0$-sample, and analyzed by chiral GC (Table S3)

**Table S3.** Time course of the catalytic epoxidation of (*R*)-undec-1-en-3-ol (**3a**) with the titanium salalen catalyst *ent*-**2**.

| Entry | Reaction time [h] | Conversion [%][a] | Yield of **4a** [%][a] | *dr* [a,b] |
|---|---|---|---|---|
| 1 | 1 | 7 | 2 | 1:13 |
| 2 | 2 | 15 | 6 | 1:13 |
| 3 | 3 | 22 | 11 | 1:14 |
| 4 | 4 | 31 | 16 | 1:15 |
| 5 | 5 | 38 | 21 | 1:15 |
| 6 | 24 | 92 | 70 | 1:18 |
| 7 | 48 | 95 | 77 | 1:18 |

[a] Determined by GC on chiral stationary phase. [b] *syn:anti*.

*2.5.7. Epoxidation of (S)-(1-methoxyallyl)cyclohexane (**6**)*

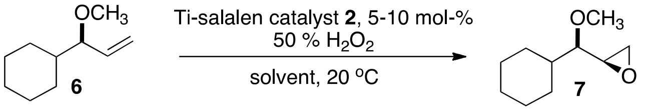


The catalytic epoxidation was performed on a 100 µmol scale, using the enantiopure allylic ether (*S*)-(1-methoxyallyl)cyclohexane (**6**) as the substrate. According to the *general procedure for catalytic epoxidations*, various amounts of $H_2O_2$ (150 µmol, 1.50 eq) were employed in different chloroform or DCE as solvent. Aliquots were analyzed by chiral GC (Table S4). The configuration of the product **7** was verified by O-methylation of enantiopure (*R*,*R*)-cyclohexyl(oxiran-2-yl)methanol (**4c**) and comparison of the NMR and GC data.

**Table S4.** Catalytic epoxidation of (*S*)-(1-methoxyallyl)cyclohexane (**6**) under various conditions.

| Entry | Solvent | Ti-catalyst **2** [mol %] | $H_2O_2$ [eq] | Epoxide yield[a] (reaction time) | *ee*[a] | *dr*[a,d] |
|---|---|---|---|---|---|---|
| 1 | $CHCl_3$ | 5 | 1.5 | 24 (48 h) | >99% | >99:1 |
| 2 | $CHCl_3$ | 5 | 7.5[b] | 33 (48 h)<br>49 (168 h) | >99% | >99:1 |
| 3 | $CHCl_3$ | 10 | 3[c] | 46 (48 h)<br>48 (168 h) | >99% | >99:1 |
| 4 | DCE | 5 | 3[c] | 68 (48 h)<br>71 (168 h) | >99% | >99:1 |

[a] Determined by GC on chiral stationary phase, [b] 1.5 eq $H_2O_2$ every 24 h, [c] 2nd portion $H_2O_2$ after 48 h. [d] *syn:anti*.

## 2.6. Analytical data of hitherto unknown epoxide products

**(*R*)-2,2-Dimethyl-1-((*R*)-oxiran-2-yl)propan-1-ol (4d)**

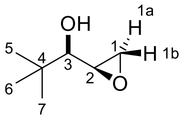


Pale yellow liquid; $R_f$ = 0.29 ($SiO_2$, *n*pentane:$Et_2O$ = 7:3); $^1$H-NMR (500 MHz, $CDCl_3$) δ [ppm] = = 3.13 – 3.09 (m; 1H, H2), 3.09 – 3.05 (m; 1H, H3), 2.82 (dd, $^2J_{H1b\text{-}H1a}$ = 5.0 Hz, $^3J_{H1b\text{-}H2}$ = 4.0 Hz; 1H, H1b), 2.69 (dd, $^2J_{H1a\text{-}H1b}$ = 5.0, $^3J_{H1a\text{-}H2}$ = 2.8 Hz; 1H, H1a), 1.99 (s, 1H, OH), 1.00 (s; 9H, H5-7); $^{13}$C-NMR (125 MHz, $CDCl_3$) δ [ppm] =78.4 (1C, C3), 52.1 (1C, C2), 45.4 (1C, C1), 34.9 (1C, C4), 25.8 (3C, C5-7); IR (ATR): ṽ [$cm^{-1}$] = 3461 (br), 2956 (s), 2871 (s), 1704 (w), 1481 (s), 1417 (w), 1397 (m), 1365 (s), 1287 (w), 1256 (s), 1187 (s), 1135 (w), 1107 (s), 1061 (s), 1011 (s), 990 (w), 928 (s), 904 (s), 880 (s), 846 (s), 809 (m), 767 (m), 745 (m), 672 (m), 526 (s); GC-MS: $\tau_R$ = 6.5 min, m/z = 129.9 (M), 115.1 (M-$CH_3$), 98.1 (M-$CH_4O$), 85.1, 74.1, 69.1, 57.1 ($C_4H_9$).

**(*R*)-2-((*R*)-Cyclohexyl(methoxy)methyl)oxirane (7)**

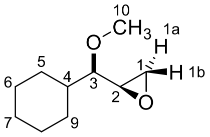


Colorless liquid; $R_f$ = 0.25 ($SiO_2$, *c*Hex:EtOAc = 10:1); $^1$H-NMR (500 MHz, $CDCl_3$) δ [ppm] = 3.46 (s; 3H, H10), 2.96 (ddd, $^3J_{H2\text{-}H3}$ = 7.2 Hz, $^3J_{H2\text{-}H1b}$ =4.2 Hz, $^3J_{H2\text{-}H1a}$ = 2.8 Hz; 1H, H2), 2.77 (dd, $^2J_{H1b\text{-}H1a}$ = 4.9 Hz, $^3J_{H1b\text{-}H2}$ =4.3 Hz; 1H, H1b), 2.55 – 2.50 (m; 1H, H3), 2.48 (dd, $^2J_{H1a\text{-}H1b}$ = 4.9 Hz, $^3J_{H1a\text{-}H2}$ = 2.8 Hz; 1H, H1a), 1.89 – 1.82 (m; 1H, H5), 1.78 – 1.69 (m; 3H, H6, H8, H9), 1.69 – 1.62 (m; 1H, H7), 1.62 – 1.50 (m; 1H, H4), 1.29 – 0.99 (m; 5H, H5, H6, H7, H8, H9); $^{13}$C-NMR (125 MHz, $CDCl_3$) δ [ppm] = 87.1 (1C, C3), 58.6 (1C, C10), 53.7 (1C, C2), 43.6 (1C, C1), 41.4 (1C, C4), 29.3 (1C, C5), 29.2 (1C, C9), 26.6 (1C, C7), 26.4 (1C, C6), 26.3 (1C, C8); IR (ATR): ṽ [$cm^{-1}$] = 3046 (w), 2980 (w), 2924 (s), 2853 (s), 2826 (m), 1450 (s), 1410 (w), 1310 (w), 1255 (w), 1185 (w), 1144 (w), 1102 (s), 1085 (s), 976 (m), 967 (m), 912 (s), 886 (s), 854 (s), 838 (s), 815 (s), 795 (w), 690 (w), 671 (w), 618 (m), 515 (s); GC-MS: $\tau_R$ = 10.1 min, m/z = 152.0 (M-$H_2O$), 138.1 (M-$CH_4O$), 127.1 (M-$C_2H_3O$), 95.1, 87.0 (M-$C_6H_{11}$), 79.1, 67.1, 55.1; HR-GC-MS: $\tau_R$ = 14.41 min, measured: m/z = 127.11160, theoretical: m/z 127.11229 (M-$C_2H_3O$); elemental analysis calcd (%) for $C_{10}H_{18}O_2$: C, 70.55; H, 10.66; found: C, 70.38; H, 10.55.

## 2.7. Synthesis of the THF-building block 8

### Synthesis of (*R*)-1-[(*R*)-oxiran-2-yl]tridecan-1-ol (4f)

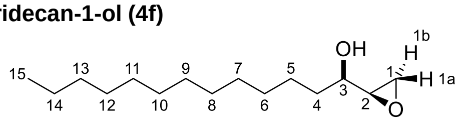

(*R*)-Pentadec-1-en-3-ol (**3f**) (453 mg, 2.00 mmol, 1.00 eq), 81 mg diphenyl ether (0.48 mmol, 0.25 eq) and 58 mg of the titanium salalen catalyst **2** (0.04 mmol, 0.02 eq) were dissolved in 10 mL chloroform. No inert atmosphere was applied. The solution was thermostated to 20 °C, and 170.5 µL $H_2O_2$ (3.00 mmol, 1.50 eq, 50% w/w in water) were added. The biphasic mixture was stirred at 20 °C for 48 h. The reaction mixture was then passed through $MgSO_4$:$MnO_2$ and eluted with dichloromethane. The solvent was evaporated under reduced pressure, and the crude product was purified two times by flash column chromatography on silica (*c*Hex:EtOAc = 4:1, then DCM:MeOH = 98:2). The product was obtained as a colorless solid. Yield: 477 mg (1.97 mmol, 98%); $R_f$ = 0.19 ($SiO_2$, *c*Hex:EtOAc = 4:1); m.p. 47.5 – 48.8 °C (lit. m.p.: 49.5 – 49.9 ºC); $^1$H-NMR (500 MHz, $CDCl_3$) δ [ppm] = 3.42 (br s; 1H, H3), 2.97 (ddd, $^3J_{H2\text{-}H3}$ = 5.1, $^3J_{H2\text{-}H1b}$ = 4.1, $^3J_{H2\text{-}H1a}$ = 2.8 Hz; 1H, H2), 2.81 (dd, $^2J_{H1b\text{-}H1a}$ = 4.9 Hz, $^3J_{H1b\text{-}H2}$ = 4.1 Hz; 1H, H1b), 2.71 (dd, $J_{H1a\text{-}H1b}$ = 4.9 Hz, $J_{H1a\text{-}H2}$ = 2.8 Hz; 1H, H1a), 1.95 (br s; 1H, OH), 1.64 – 1.53 (m; 2H, H4), 1.52 – 1.41 (m; 1H, H5), 1.41 – 1.18 (m; 19H, H5-14), 0.87 (t, $^3J_{H15\text{-}H14}$ = 7.0 Hz; 3H, H15); $^{13}$C-NMR (125 MHz, $CDCl_3$) δ [ppm] = 71.8 (1C, C3), 55.5 (1C, C2), 45.3 (1C, C1), 34.6 (1C, C4), 32.1 (1C, C13), 29.8 ($CH_2$), 29.8 ($CH_2$), 29.7 ($CH_2$), 29.5 ($CH_2$), 25.4 (1C, C5), 22.8 (1C, C14), 14.3 (1C, C15); IR (ATR): ṽ [$cm^{-1}$] = 3662 (w), 3362 (br), 3291 (br), 2962 (w), 2916 (s), 2849 (s), 1473 (s), 1463 (s), 1404 (m), 1338 (w), 1255 (w), 1125 (m), 1068 (m), 1031 (w), 963 (s), 889 (s), 874 (s), 824 (w), 791 (w), 752 (s), 729 (s), 720 (s), 664 (m), 648 (m), 541 (w), 509 (w); GC-MS: $\tau_R$ = 13.7 min, m/z = 208.3 (M–$H_2O_2$), 199.1 (M-$C_3H_7$ / M-$C_2H_3O$), 166.2, 152.1, 137.3, 125.1 ($C_8H_{13}O$), 111.2 ($C_7H_{11}O$), 97.1 ($C_6H_9O$), 83.1($C_5H_7O$), 69.1 ($C_4H_5O$), 55.1; Analytic data are in agreement with the literature.[3]

### Synthesis of *tert*-butyl[((*R*)-1-[(*R*)-oxiran-2-yl]tridecyl)oxy]diphenylsilane (9)

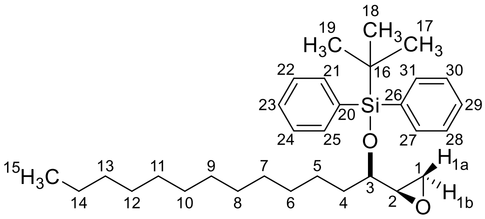

The reaction was carried out according to a modified procedure by *Trost* and *Rey*.[17] (*R*)-1-[(*R*)-oxiran-2-yl]tridecan-1-ol (**4f**) (408 mg, 1.68 mmol, 1.00 eq) was dissolved in 7 mL DCM. No inert atmosphere was applied. The solution was cooled to 0 °C, and 344 mg (5.05 mmol, 3.00 eq) imidazole and 525 µL *tert*-butyl(chloro)diphenylsilane (2.02 mmol, 1.20 eq) were added. The reaction mixture was stirred at room temperature overnight. The reaction mixture was then diluted with 20 mL DCM and washed with 20 mL water. The aqueous phase was extracted two times with 10 mL DCM. The combined organic phases were washed with brine, dried over $MgSO_4$, filtered, and the solvent was evaporated under reduced pressure. The product was obtained as a colorless liquid and used without further purification. Yield: 784 mg (1.63 mmol, 97%); $R_f$ = 0.26 ($SiO_2$, *c*Hex:DCM = 7:3); $^1$H-NMR (500 MHz, $CDCl_3$) δ [ppm] = 7.77 – 7.64 (m; 4H, H21, H25, H27, H31), 7.46 – 7.40 (m; 2H, H23 + H29), 7.40 – 7.32 (m; 4H, H22, H24, H28, H30), 3.36 (dd, $^3J_{H3\text{-}H2}$ = $^3J_{H3\text{-}H4}$ = 6.3 Hz; 1H, H3), 3.05 (ddd, $^3J_{H2\text{-}H3}$ = 6.7 Hz, $^3J_{H2\text{-}H1b}$ = 4.1 Hz, $^3J_{H2\text{-}H1a}$ = 2.7 Hz, 1H, H2), 2.72 (dd, $^2J_{H1b\text{-}H1a}$ = 4.9 Hz, $^3J_{H1b\text{-}H2}$ = 4.2 Hz, 1H, H1b), 2.47 (dd, $^2J_{H1a\text{-}H1b}$ = 4.9 Hz, $^3J_{H1a\text{-}H2}$ = 2.7 Hz; 1H, H1a), 1.55 – 1.42 (m; 2H, H4), 1.38 – 1.00 (m; 20H, H5-14), 1.10 (s; 9H, H17-19), 0.90 (t, $^3J_{H15\text{-}H14}$ = 7.0 Hz; 3H, H15); $^{13}$C-NMR (125 MHz, $CDCl_3$) δ [ppm] = 136.1 (2C, C21, C25), 136.1 (2C, C27, C31), 134.4 (1C, C20), 134.1 (1C, C26), 129.7 (1C, C23), 129.7 (1C, C29), 127.6 (2C, C22, C24), 127.5 (2C, C28, C30), 75.3 (1C, C3), 55.8 (1C, C2), 45.0 (1C, C1), 34.9 (1C, C4), 32.1 (1C, C13), 29.8 (1C, $CH_2$), 29.8 (1C, $CH_2$), 29.8 (1C, $CH_2$), 29.7 (1C, $CH_2$), 29.7 (1C, $CH_2$), 29.6 (1C, $CH_2$), 29.5 (1C, $CH_2$), 27.2 (3C, C17-19), 25.0 (1C, C5), 22.9 (1C, C14), 19.6 (1C, C16), 14.3 (1C, C15); IR (ATR): ṽ [$cm^{-1}$] = 3662 (w), 3072 (w), 3049 (w), 2924 (s), 2854 (s), 1958 (w), 1891 (w), 1823 (w), 1590 (w), 1464 (m), 1428 (s), 1391 (w), 1362 (w), 1308 (w), 1257 (w), 1105 (s), 1071 (s), 999 (w), 929 (br), 844 (w), 822 (m), 739 (m), 700 (s), 611 (s), 507 (s); GC-MS: $\tau_R$ = 16.8 min, m/z = 503.1, 465.3 (M-$CH_3$), 423.3 (M-$C_4H_9$), 393.3, 345.2, 225.1 (M-$C_{16}H_{19}OSi$), 199.1, 165.0, 139.0; elemental analysis calcd (%) for $C_{31}H_{48}O_2Si$: C, 77.44; H, 10.06; found: C, 77.45; H, 10.08; Analytic data are in agreement with the literature.[18]

**Synthesis of (5*R*,6*R*)-6-[(*tert*-butyldiphenylsilyl)oxy]octadec-1-en-5-ol (10)**

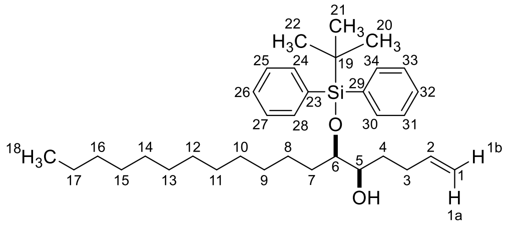

The reaction was carried out according to a modified procedure by *Trost* and *Rey*.[17] *tert*-butyl[((*R*)-1-[(*R*)-oxiran-2-yl]tridecyl)oxy]diphenylsilane (**9**) (723 mg, 1.50 mmol, 1.00 eq) was dissolved in 15 mL dry THF under argon atmosphere. 50 mg CuI (0.26 mmol, 0.17 eq) was added, and the suspension was cooled to –78 °C. Allylmagnesium bromide (4.5 mL, 4.50 mmol, 3.00 eq, 1M solution in $Et_2O$,) was added in a dropwise manner, with stirring. The reaction mixture was allowed to warm to rt, and was left stirring for 1 h. The reaction mixture was then cooled to 0 °C and quenched with 20 mL sat. aq. $NH_4Cl$-solution. The phases were separated, and the aqueous phase was extracted two times with 20 mL diethyl ether. The combined organic phases were washed with brine, dried over $MgSO_4$, filtered, and the solvent was evaporated under reduced pressure. The crude product was purified by flash column chromatography on silica (*c*Hex:$Et_2O$ = 24:1). The product was obtained as a colorless liquid. Yield: 662 mg (1.27 mmol, 84%); $R_f$ = 0.30 ($SiO_2$, *c*Hex:$Et_2O$ = 24:1); $^1$H-NMR (500 MHz, $CDCl_3$) δ [ppm] = 7.72 – 7.66 (m; 4H, H24, H28, H30, H34), 7.46 – 7.41 (m; 2H, H26, H32), 7.41 – 7.34 (m; 4H, H25, H27, H31, H33), 5.79 (ddt, $^3J_{H2\text{-}H1a}$ = 16.9 Hz, $^3J_{H2\text{-}H1b}$ = 10.2 Hz, $^3J_{H2\text{-}H3}$ = 6.6 Hz; 1H, H2), 5.01 – 4.96 (m; 1H, H1a), 4.96 – 4.92 (m; 1H, H1b), 3.61 – 3.54 (m; 1H, H6), 3.54 – 3.46 (m; 1H, H5), 2.22 (d, $^3J_{OH\text{-}H5}$ = 7.1 Hz; 1H, OH), 2.20 – 2.11 (m; 1H, H3), 2.08 – 1.97 (m; 1H, H3), 1.64 – 1.53 (m; 2H, H4, H7), 1.53 – 1.40 (m; 1H, H4), 1.39 – 0.93 (m; 21 H, H7-17), 1.08 (s; 9H, H20-22), 0.90 (t, *J* = 7.0 Hz; 3H, H18); $^{13}$C-NMR (125 MHz, $CDCl_3$) δ [ppm] = 138.5 (1C, C5), 136.0 (2C, C24, C28), 135.9 (2C, C30, C34), 134.1 (1C, C23), 133.5 (1C, C29), 129.8 (1C, C26), 129.7 (1C, C32), 127.7 (2C, C25, C27), 127.5 (2C, C31, C33), 114.6 (1C, C1), 76.3 (1C, C6), 72.2 (1C, C5), 33.4 (1C, C7), 33.2 (1C, C4), 32.0 (1C, C16), 30.1 (1C, C3), 29.8 ($CH_2$), 29.8 ($CH_2$), 29.7 ($CH_2$), 29.6 ($CH_2$), 29.6 ($CH_2$), 29.5 ($CH_2$), 29.5 ($CH_2$), 27.3 (3C, C20-22), 25.0 (1C, C8), 22.8 (1C, C17), 19.6 (1C, C19), 14.2 (1C, C18); IR (ATR): ṽ [$cm^{-1}$] = 3578 (w), 3073 (w), 3051 (w), 2924 (s), 2854 (s), 1957 (w), 1889 (w), 1823 (w), 1641 (w), 1590 (w), 1464 (m), 1428 (s), 1390 (w), 1378 (w), 1362 (w), 1306 (w), 1261 (w), 1190 (w), 1110 (s), 1074 (br), 998 (m), 910 (m), 821 (m), 739 (m), 700 (s), 608 (m), 505 (s); GC-MS: $\tau_R$ = 18.7 min, m/z = 490.3, 465.3 (M-$C_4H_9$), 437.4 (M-$C_5H_9O$), 409.3 (M-$C_8H_{17}$), 387.3, 355.2, 325.4, 281.0, 249.3, 199.1, 139.0, 109.1, 81.1, 57.1; Analytic data are in agreement with the literature.[18]

**Synthesis of (2*R*,5*R*)-5-[(*R*)-1-[(*tert*-butyldiphenylsilyl)oxy]tridecyl)tetrahydrofuran-2-yl]methanol (8)**

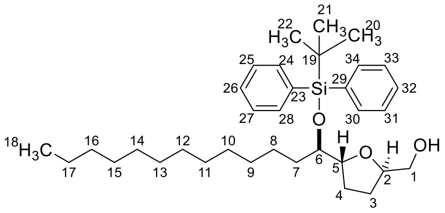

(5*R*,6*R*)-6-[(*tert*-Butyldiphenylsilyl)oxy]octadec-1-en-5-ol (**10**) (1.69 g, 3.23 mmol, 1.00 eq) and the titanium salalen catalyst **2** (233 mg, 0.16 mmol, 0.05 eq) were dissolved in 16 mL chloroform. No inert atmosphere was applied. The solution was thermostated to 20 °C, and $H_2O_2$ (275 µL, 4.85 mmol, 1.50 eq, 50% w/w in water) was added. The biphasic mixture was stirred at 20 °C. After 24 h, 48 h, and 72 h, 1.50 eq of $H_2O_2$ were additionally added. After 96 h, the reaction mixture passed through $MgSO_4$:$MnO_2$ and eluted with chloroform. The solvent was evaporated under reduced pressure, and the crude product was purified two times by flash column chromatography on silica (*c*Hex:EtOAc = 9:1 to 4:1, then 100% DCM). The product was obtained as a colorless liquid. Yield: 1.38 g (2.57 mmol, 80%); $R_f$ = 0.42 ($SiO_2$, *c*Hex:EtOAc = 4:1); $^1$H-NMR (500 MHz, $CDCl_3$) δ [ppm] = 7.80 – 7.65 (m; 4H, H24, H28, H30, H34), 7.45 – 7.39 (m; 2H, H26, H32), 7.39 – 7.33 (m; 4H, H25, H27, H31, H33), 3.93 (ψt, $J_{H5\text{-}H6}$ = 7.9 Hz, $J_{H5\text{-}H4}$ = 6.8 Hz; 1H, H5), 3.76 – 3.68 (m; 1H, H2), 3.61 – 3.50 (m; 1H, H6), 3.36 (dd, $J_{H1a\text{-}H1b}$ = 11.5 Hz, $J_{H1a\text{-}H2}$ = 3.0 Hz; 1H, H1a), 3.20 (dd, $J_{H1b\text{-}H1a}$ = 11.5 Hz, $J_{H1b\text{-}H2}$ = 6.5 Hz; 1H, H1b), 1.96 – 1.70 (m; 2H, H4, H3), 1.57 – 1.45 (m; 2H, H3, H4), 1.45 – 1.09 (m; 22H, H7-17), 1.05 (s; 9H, H20-22), 0.90 (t, $J_{H18\text{-}H17}$ = 6.9 Hz; 3H, H18); $^{13}$C-NMR (125 MHz, $CDCl_3$) δ [ppm] = 136.3 (2C, C30, C34), 135.9 (2C, C24, C28), 135.6 (1C, C29), 134.3 (1C, C23), 129.4 (1C, C32), 129.3 (1C, C26), 127.4 (2C, C31, C33), 127.2 (2C, C25, C27), 82.2 (1C, C5), 79.2 (1C, C2), 76.6 (1C, C6), 65.1 (1C, C1), 33.4 (1C, C7), 32.1 (1C, C16), 29.8 ($CH_2$), 29.8 ($CH_2$), 29.8 ($CH_2$), 29.8 ($CH_2$), 29.7 ($CH_2$), 29.7 ($CH_2$), 29.5 ($CH_2$), 28.6 (1C, C3), 27.8 (1C, C4), 27.3 (3C, C20-22), 25.3 (1C, C8), 22.8 (1C, C17), 19.8 (1C, C19), 14.3 (1C, C18); IR (ATR): ṽ [$cm^{-1}$] = 3571 (w), 3453 (br), 3071 (w), 3049 (w), 1957 (w), 1889 (w), 1823 (w), 1590 (w), 1464 (m), 1428 (m), 1389 (w), 1378 (w), 1361 (w), 1329 (w), 1261 (w), 1190 (w), 1110 (s), 1051 (s), 998 (w), 939 (w), 889 (w), 822 (m), 739 (m), 700 (s), 609 (m), 507 (s); GC-MS: $\tau_R$ = 22.5 min, m/z = 507.3 (M-$CH_3O$), 481.4 (M-$C_4H_9$), 461.0, 437.4 (M-$C_5H_9O_2$), 403.4, 379.1, 355.2, 281.1, 239.1 ($C_{16}H_{19}Si$), 199.1, 167.1, 135.1, 78.1, 57.1.

## 3. HPLC and GC-Methods

### 3.1. Allylic alcohols and related compounds

| Structure | $\tau_R$ | Chromatography and Method |
|---|---|---|
| 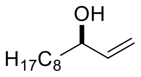  **3a** | $\tau_R$ = 39.2 min (*S*)<br>$\tau_R$ = 40.3 min (*R*) | GC, Lipodex A<br>split = 50:1; split flow = 50 mL/min, $N_2$; flow 1.0 mL/min; 93 °C isothermal 45 min, 10 °C/min to 130 °C isothermal 30 min, 10 °C/min to 180 °C isothermal 5 min. |

GC of *rac*-undec-1-en-3-ol (*rac*-**3a**):

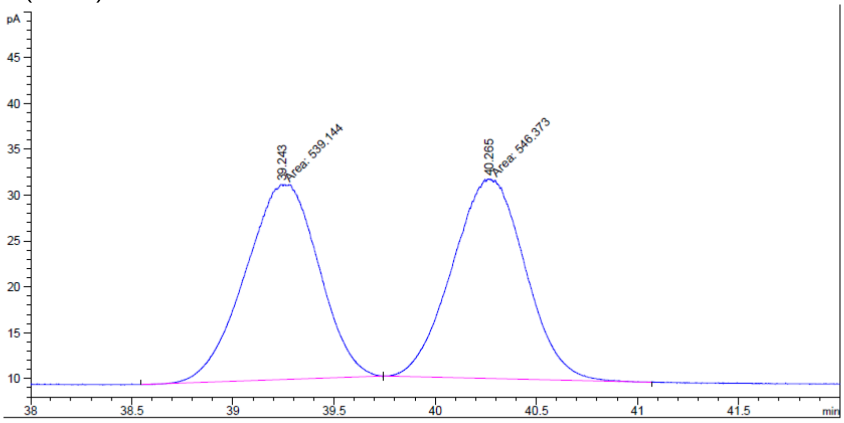


GC of the allylic alcohol **3a**, from the kinetic resolution of *rac*-undec-1-en-3-ol (*rac*-**3a**) with the enzyme CAL B, after 19 h:

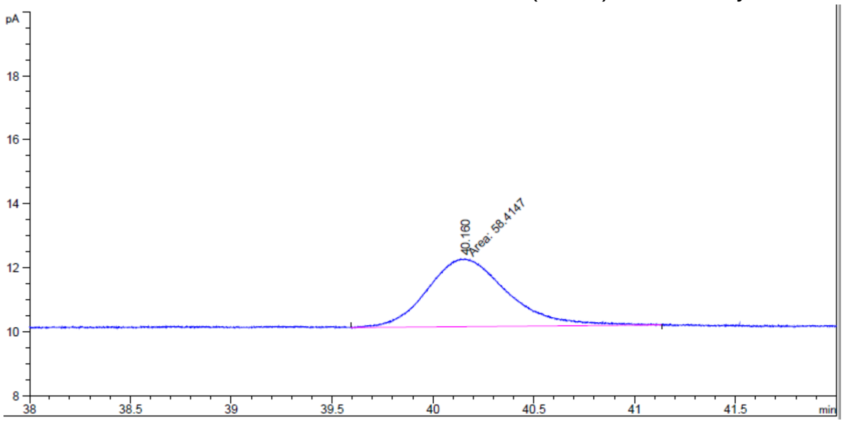


GC of *ent*-**3a**, obtained from undec-1-en-3-yl acetate (*ent*-**3a-Ac**) after ester hydrolysis:

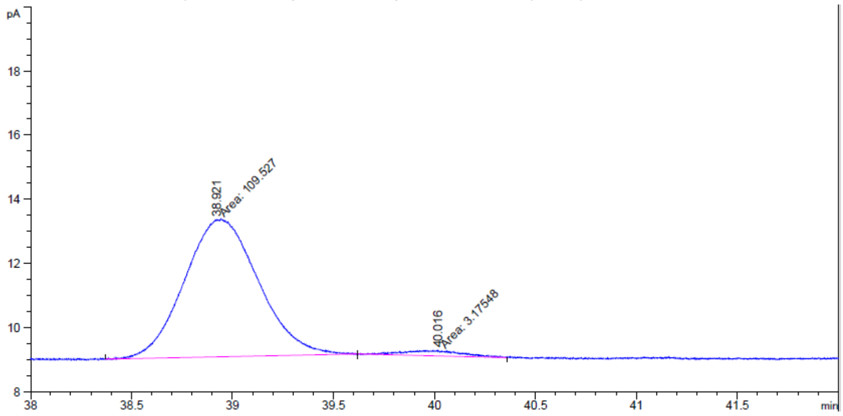

| Structure | $\tau_R$ | Chromatography and Method |
|---|---|---|
| 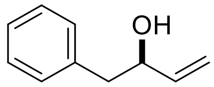 **3b** 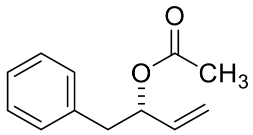 *ent*-**3b-Ac** | $\tau_R$ = 13.1 min **3b-Ac** (*R*)<br>$\tau_R$ = 15.0 min ***ent*-3b-Ac** (*S*)<br>$\tau_R$ = 20.5 min **3b** (*R*)<br>$\tau_R$ = 22.1 min *ent*-**3b** (*S*) | GC, Chirasil-Dex CB<br>split = 80:1; split flow = 160.0 mL/min, $N_2$; flow 2.0 mL/min; 100 °C isothermal 30 min, 10 °C/min to 180 °C isothermal 10 min. |

GC of *rac*-1-phenylbut-3-en-2-ol (*rac*-**3b**):

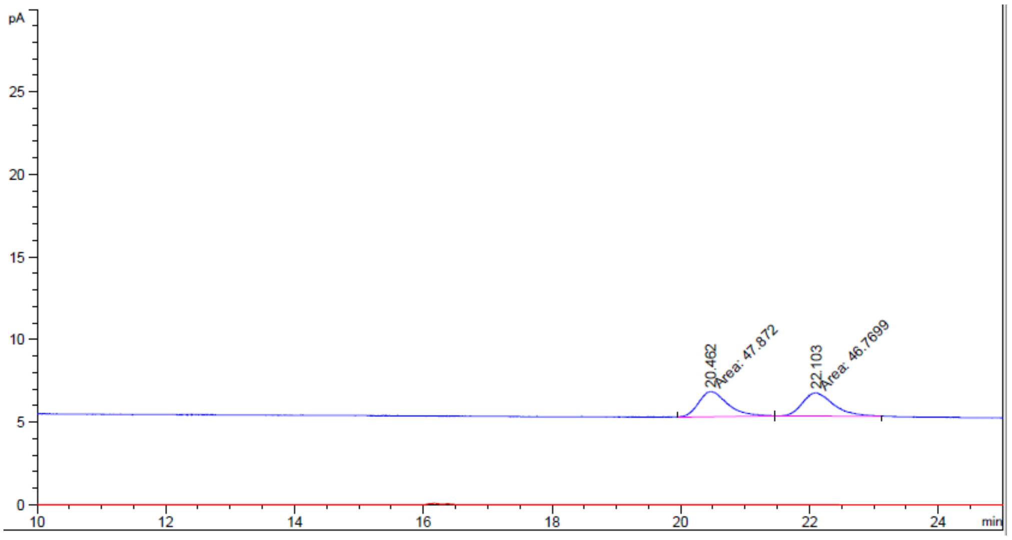


GC of the allylic alcohol **3b**, plus the acetates **3b-Ac** and *ent*-**3b-Ac**, from the kinetic resolution of *rac*-1-phenylbut-3-en-2-ol (*rac*-**3b**) with the enzyme CAL B, after 48 h:

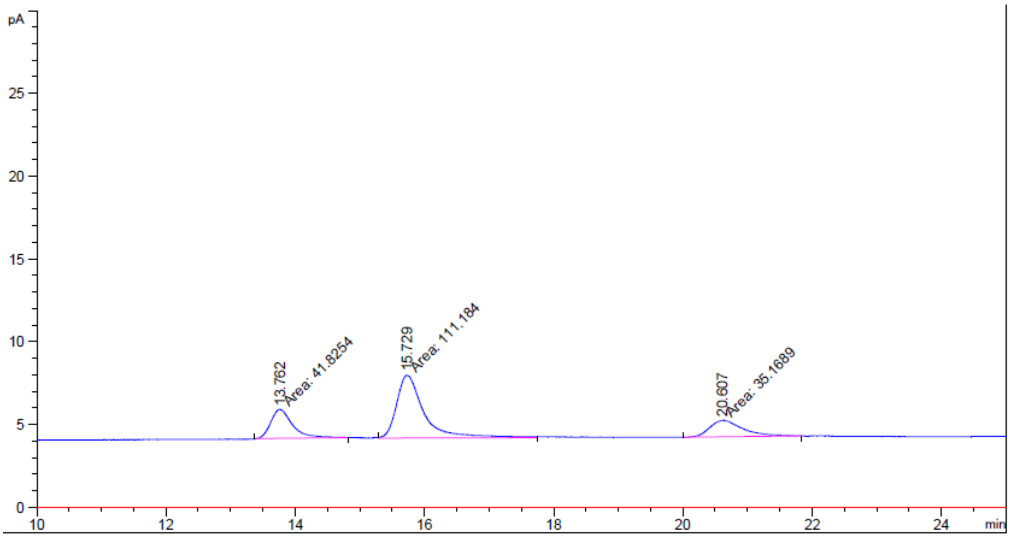

GC of *rac*-1-phenylbut-3-en-2-yl acetate (*rac*-**3b-Ac**)

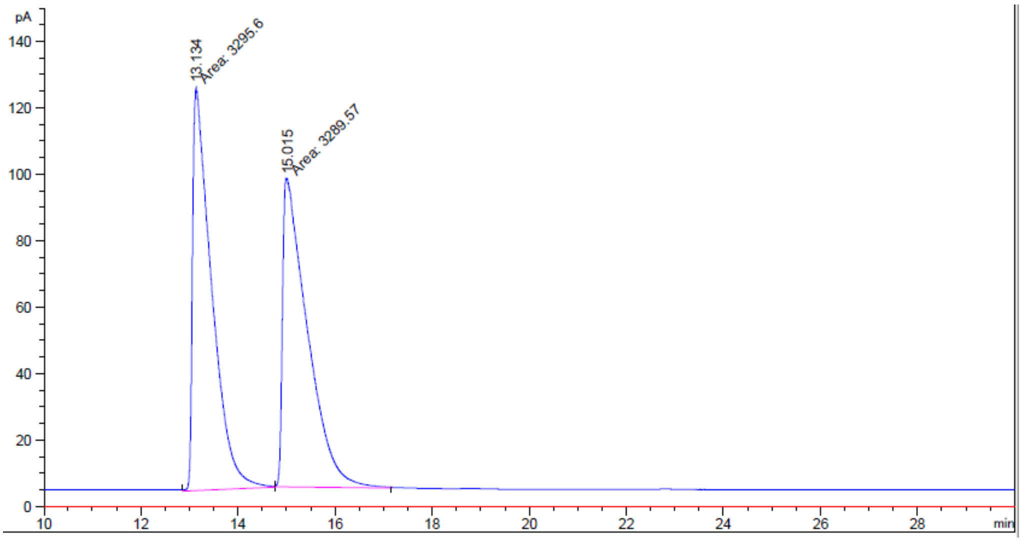

| Structure | $\tau_R$ | Chromatography and Method |
|---|---|---|
| 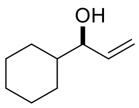 **3c** | $\tau_R$ = 40.6 min (*R*)<br>$\tau_R$ = 42.2 min (*S*) | GC, Chirasil-Dex CB<br>split = 80:1; split flow = 97.9 mL/min, $N_2$;<br>flow 1.2 mL/min; 85 °C isothermal 45 min,<br>10 °C/min to 140 °C isothermal 20 min,<br>10 °C/min to 180 °C isothermal 5 min. |

GC of *rac*-1-cyclohexylprop-2-en-1-ol (*rac*-**3c**):

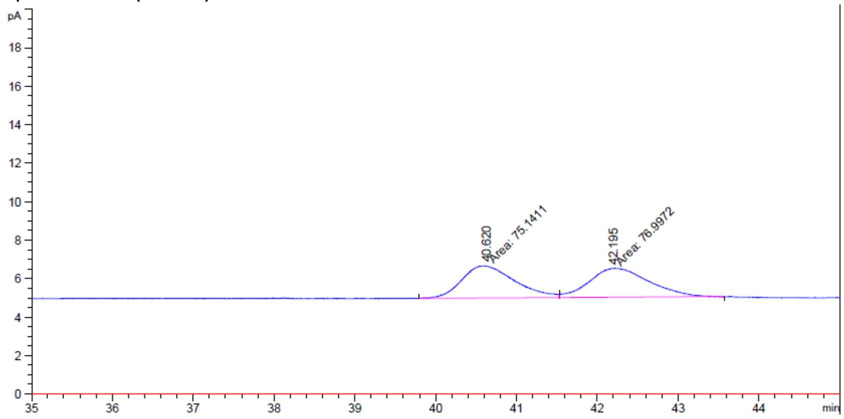


GC of the allylic alcohol **3c**, from the kinetic resolution of *rac*-1-cyclohexylprop-2-en-1-ol (*rac*-**3c**) with the enzyme CAL B, after 20 h:

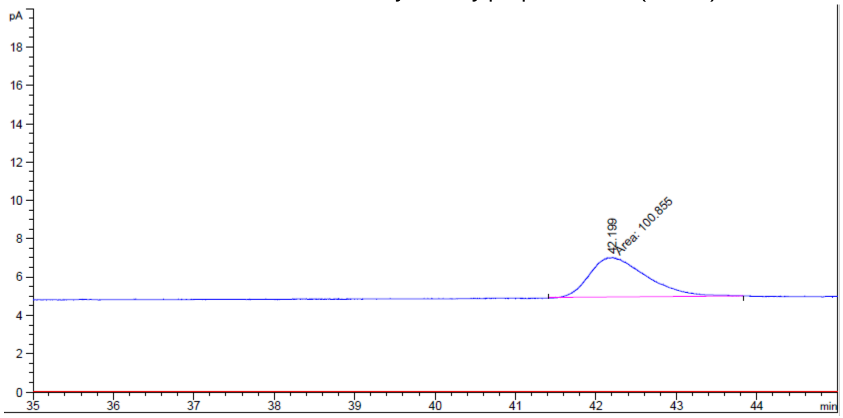


GC of *ent*-**3c**, obtained from 1-cyclohexylallyl acetate (*ent*-**3c-Ac**) after ester hydrolysis:

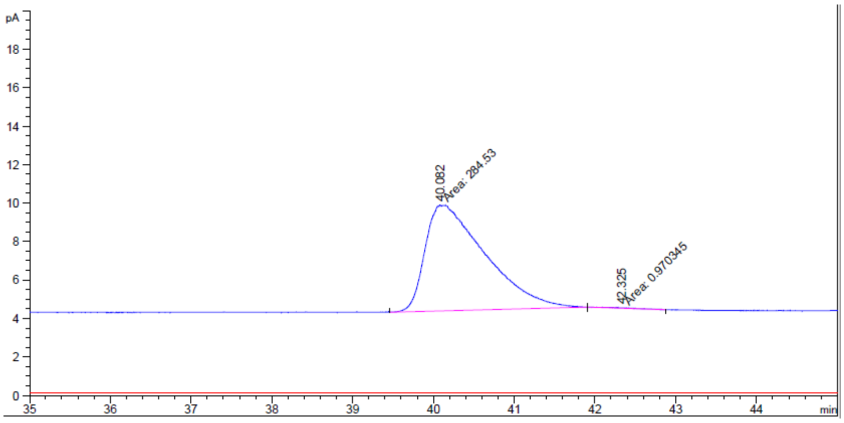

| Structure | $\tau_R$ | Chromatography and Method |
|---|---|---|
| 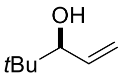 **3d** | $\tau_R$ = 29.7 min (*R*)<br>$\tau_R$ = 31.6 min (*S*) | GC, Chirasil-Dex CB<br>split = 80:1; split flow = 116.6 mL/min, $N_2$; flow 1.5 mL/min; 50 °C isothermal 38 min, 10 °C/min to 140 °C isothermal 20 min, 10 °C/min to 180 °C isothermal 5 min. |

GC of *rac*-4,4-dimethylpent-1-en-3-ol (*rac*-**3d**):

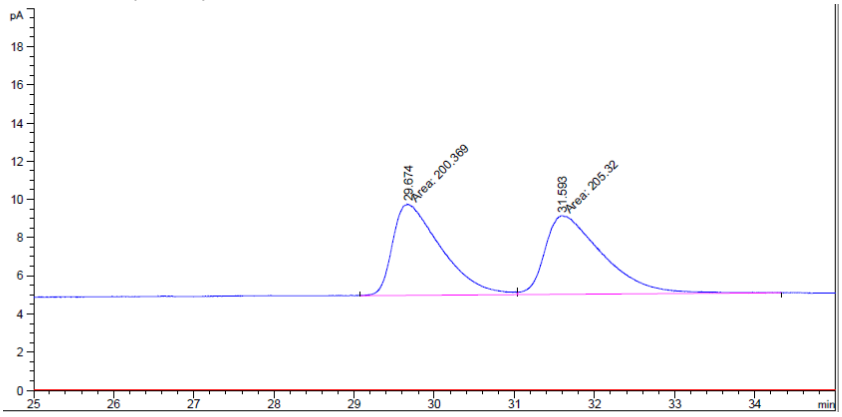


GC of the allylic alcohol **3d**, from the kinetic resolution of *rac*-4,4-dimethylpent-1-en-3-ol (*rac*-**3d**) with the enzyme CAL A, after 16 h:

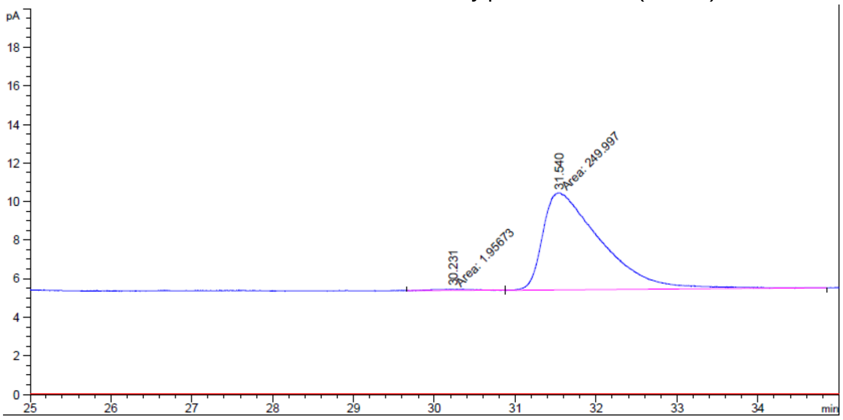


GC of *ent*-**3d** (+ **3d** as minor component), obtained from 4,4-dimethylpent-1-en-3-yl acetate (*ent*-**3d-Ac**) after ester hydrolysis:

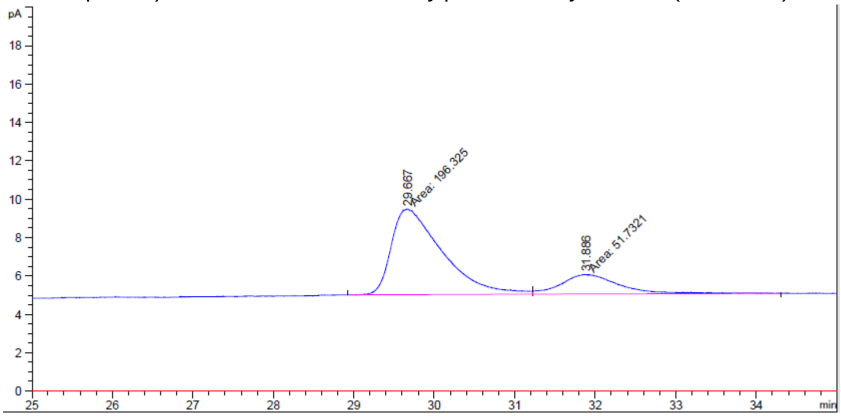

| Structure | $τ_R$ | Chromatography and Method |
|---|---|---|
| 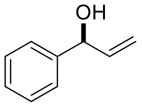  **3e** 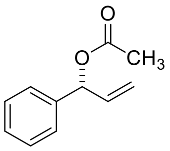  *ent*-**3e-Ac** | $τ_R$ = 34.6 min **3e-Ac** (*S*);<br>$τ_R$ = 36.2 min *ent*-**3e-Ac** (*R*)<br>$τ_R$ = 42.4 min *ent*-**3e** (*R*);<br>$τ_R$ = 43.2 min **3e** (*S*). | Chirasil-DEX CB<br>split = 50:1; split flow = 50 mL/min, $N_2$; flow 1.0 mL/min; 40 °C isothermal 1 min, 2 °C/min to 130 °C, 20 °C/min to 170 °C isothermal 5 min. |

GC of *rac*-α-vinylbenzyl alcohol (*rac*-**3e**):

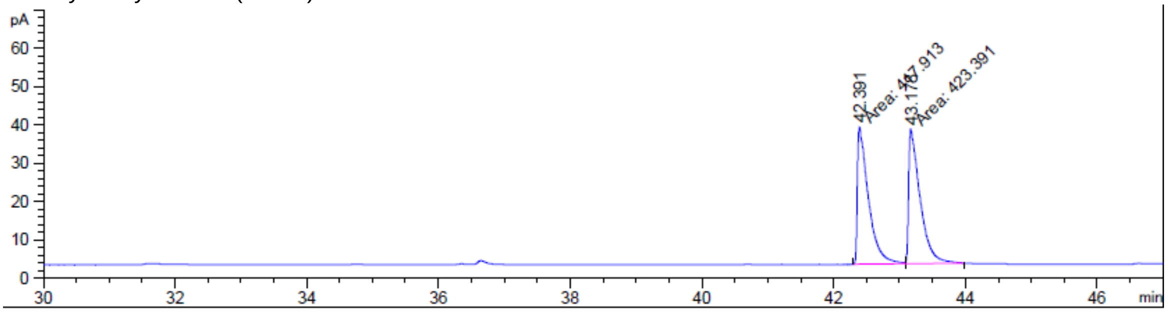


GC of the allylic alcohol **3e** plus the acetate *ent*-**3e-Ac** from the kinetic resolution of *rac*-α-vinylbenzyl alcohol (*rac*-**3e**) with the enzyme CAL B, after 48 h:

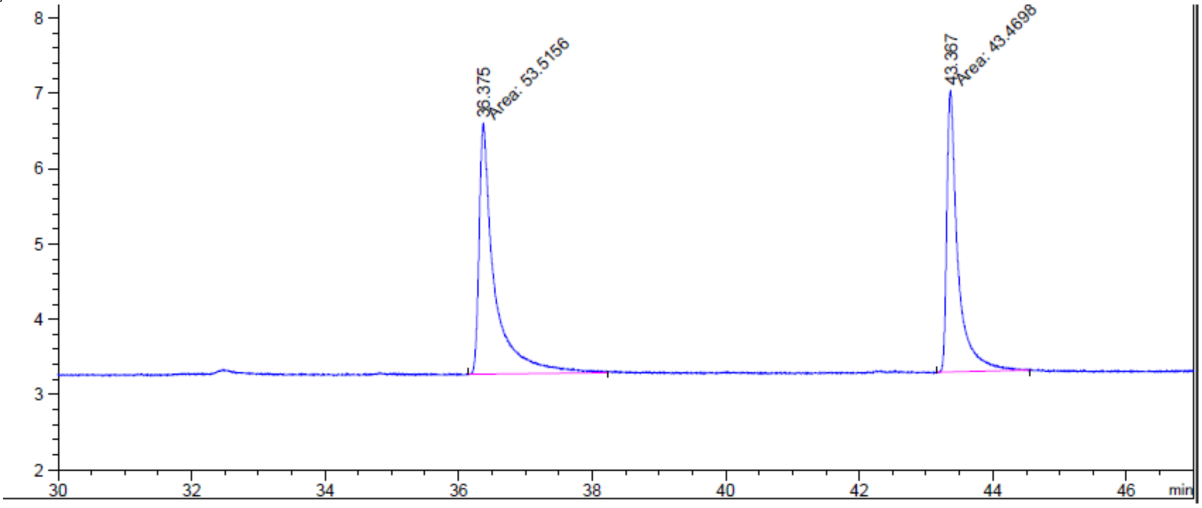


GC of *rac*-1-phenyl-2-propenyl acetate (*rac*-**3e-Ac**):

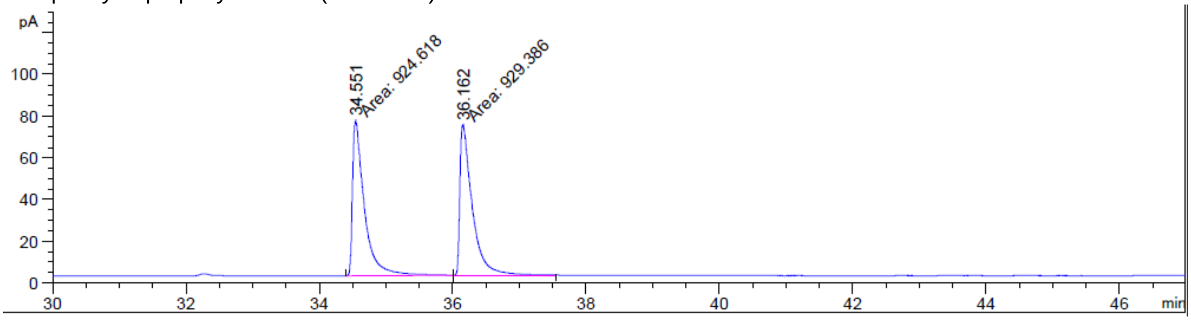

| Structure | $\tau_R$ | Chromatography and Method |
|---|---|---|
| 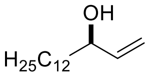 **3f** | $\tau_R$ = 13.8 min (*R*)<br>$\tau_R$ = 18.3 min (*S*) | HPLC, Daicel Chiracel OD-H<br>*n*-hexane:isopropanol = 92:2,<br>flow = 1.0 mL/min, 18 °C, fixed 210 nm. |

HPL-Chromatogram of *rac*-pentadec-1-en-3-ol (*rac*-**3f**) after derivatization with 3,5-dinitrobenzoyl chloride:

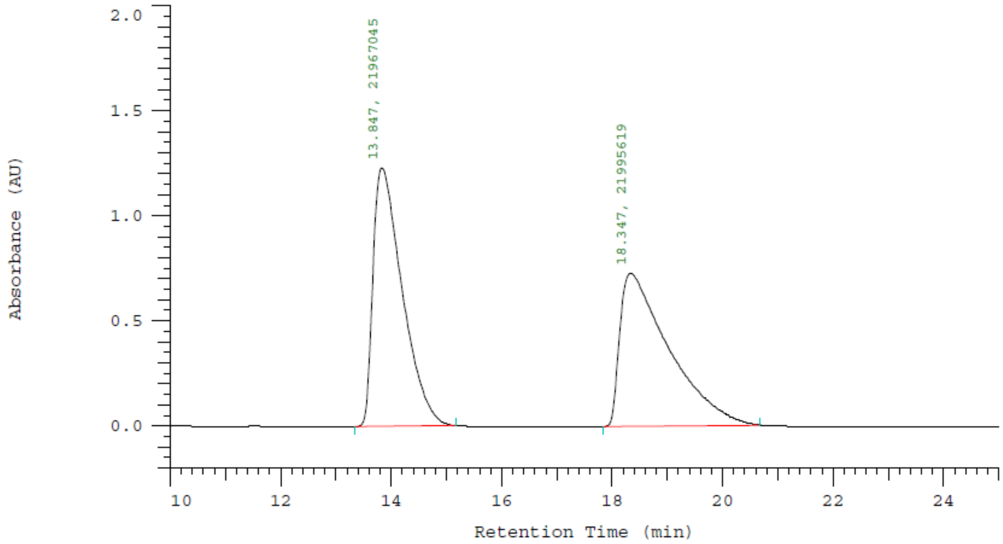


HPL-Chromatogram of the allylic alcohol **3f**, from the kinetic resolution of *rac*-pentadec-1-en-3-ol (*rac*-**3f**) with the enzyme CAL B, after 24 h and derivatization with 3,5-dinitrobenzoyl chloride:

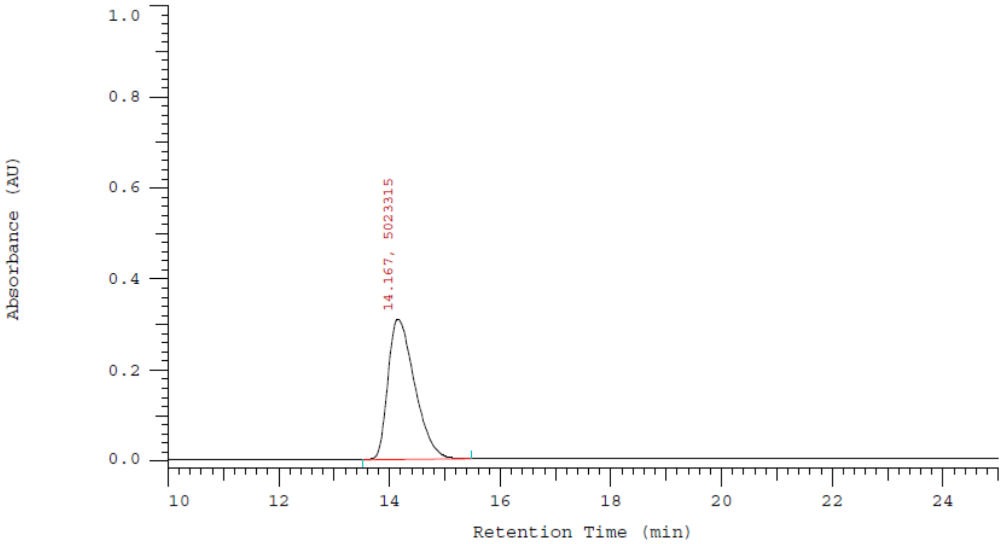

### 3.2. *syn/anti*-Epoxy alcohols obtained from *m*CPBA oxidation of racemic allylic alcohols, and *syn*-epoxy alcohols obtained from catalytic epoxidations with the titanium salalen catalyst 2

| Structure | $\tau_R$ | Chromatography and Method |
|---|---|---|
| 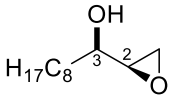<br>(2*R*,3*R*)-**4a**<br>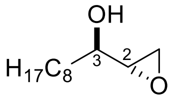<br>(2*S*,3*R*)-**4a** | $\tau_R$ = 39.2 min (*S*)-**3a**;<br>$\tau_R$ = 40.3 min (*R*)- **3a**;<br>$\tau_R$ = 49.9 min ($Ph_2O$);<br>$\tau_R$ = 59.5 min (2*S*,3*S*)-**4a**;<br>$\tau_R$ = 59.9 min (2*R*,3*S*)-**4a**;<br>$\tau_R$ = 60.5 min (2*S*,3*R*)-**4a**;<br>$\tau_R$ = 61.3 min (2*R*,3*R*)-**4a**. | GC, Lipodex A<br>split = 50:1; split flow = 50 mL/min, $N_2$;<br>flow 1.0 mL/min; 93 °C isothermal 45 min, 10 °C/min to 130 °C isothermal 30 min, 10 °C/min to 180 °C isothermal 5 min. |

GC obtained from the epoxidation of the allylic alcohol *rac*-**3a** with *m*CPBA (overview):

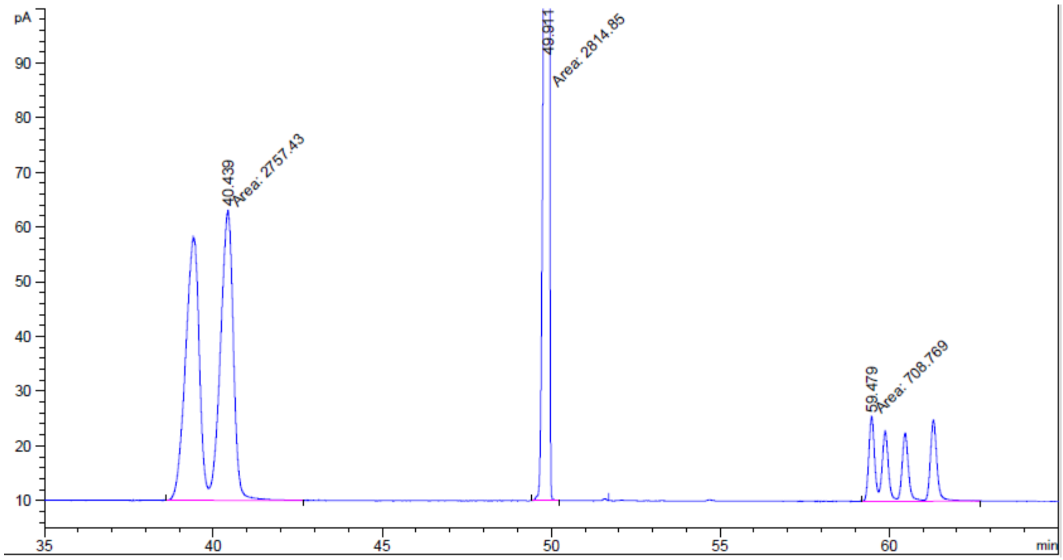


GC obtained from the epoxidation of the allylic alcohol *rac*-**3a** with *m*CPBA (epoxy alcohol region):

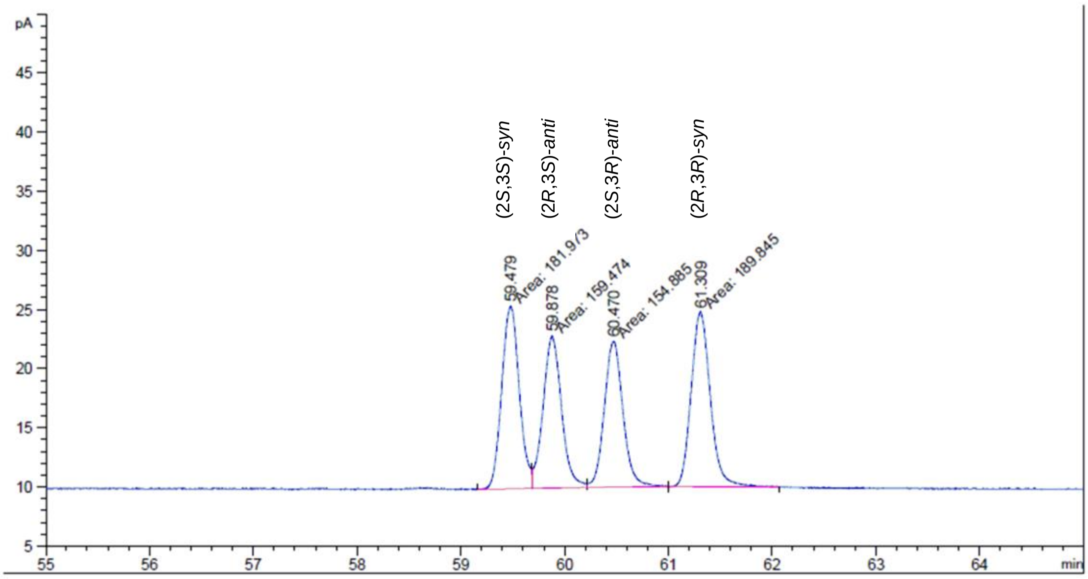

GC obtained from the epoxidation of the allylic alcohol **3a** with the titanium salalen catalyst **2** (epoxy alcohol region):

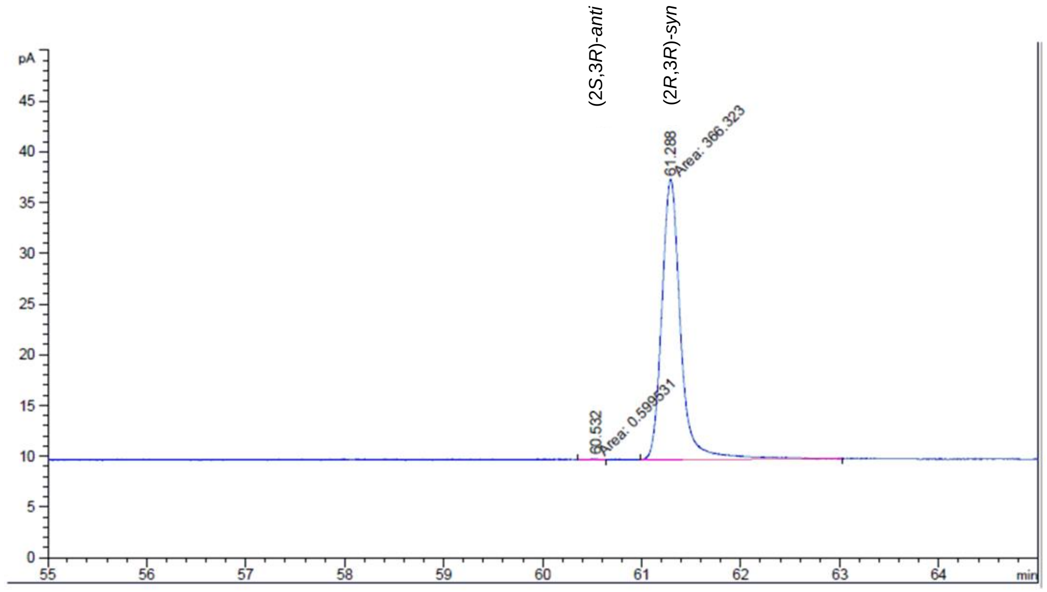


GC obtained from the epoxidation of the allylic alcohol **3a** with the titanium salalen catalyst *ent*-**2** (epoxy alcohol region):

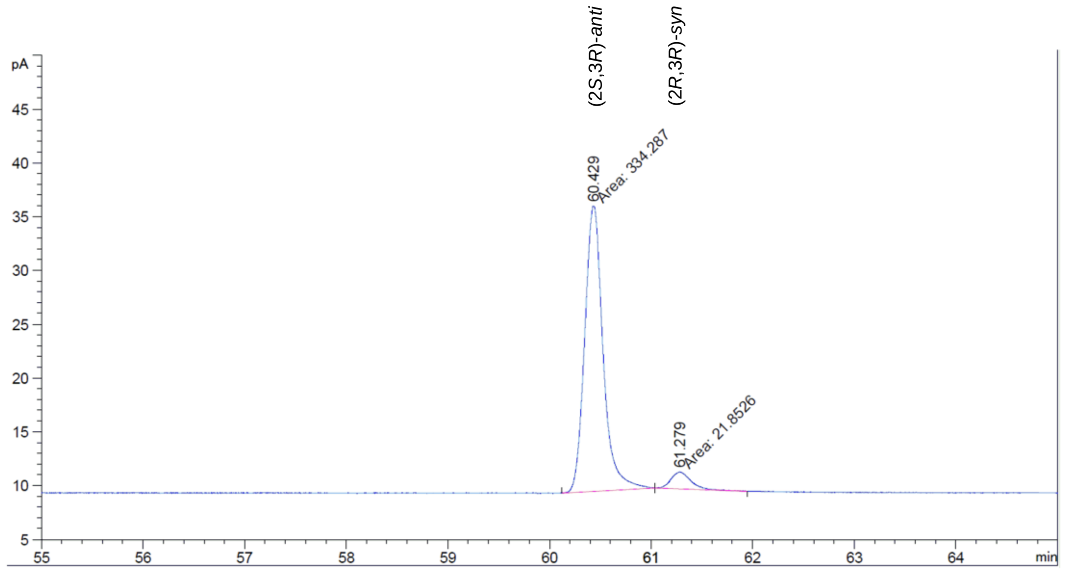

GC obtained from the kinetic resolution of *rac*-undec-1-en-3-ol (*rac*-**3a**) with the titanium salalen catalyst **2** after 24 h in DCM:

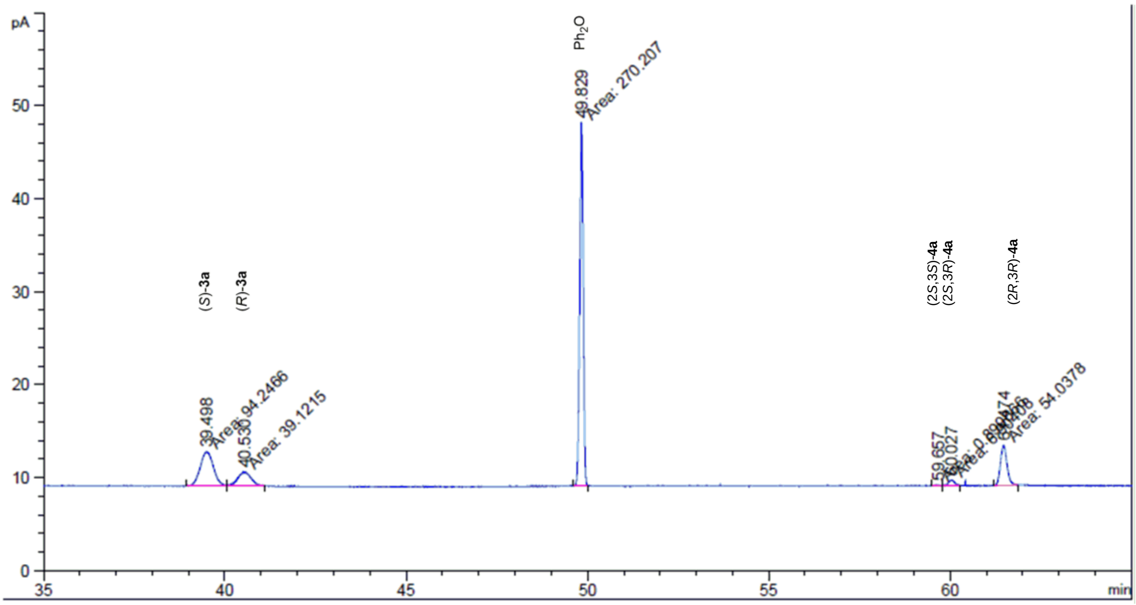


GC obtained from the kinetic resolution of *rac*-undec-1-en-3-ol (*rac*-**3a**) with the titanium salalen catalyst **2** after 24 h in acetonitrile:

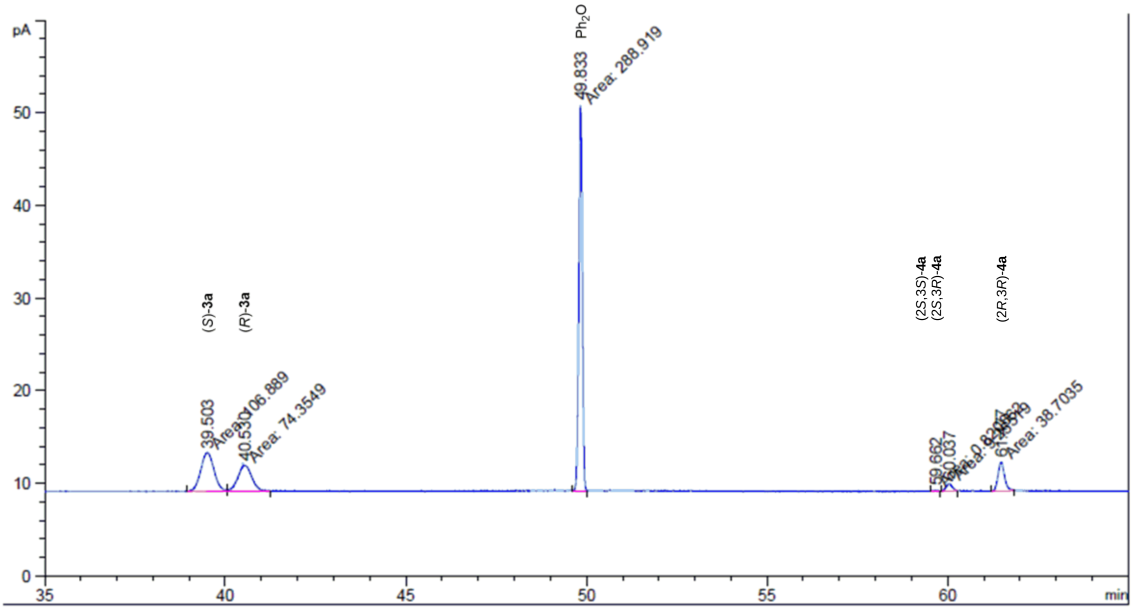

| Structure | $\tau_R$ | Chromatography and Method |
|---|---|---|
| 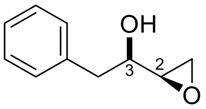  (2*R*,3*R*)-**4b** 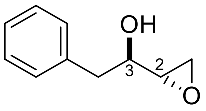  (2*S*,3*R*)-**4b** | $\tau_R$ = 28.1 min (2*R*,3*S*)- **4b**;<br>$\tau_R$ = 28.7 min (2*S*,3*R*)- **4b** and (2*S*,3*S*)- **4b**;<br>$\tau_R$ = 30.2 min (2*R*,3*R*)-**4b**. | GC, Lipodex A<br>split = 50:1; split flow = 50 mL/min, $N_2$; flow 1.0 mL/min; 120 °C isothermal 32 min, 10 °C/min to 180 °C isothermal 5 min. |

GC obtained from the epoxidation of the allylic alcohol *rac*-**3b** with *m*CPBA (epoxy alcohol region):

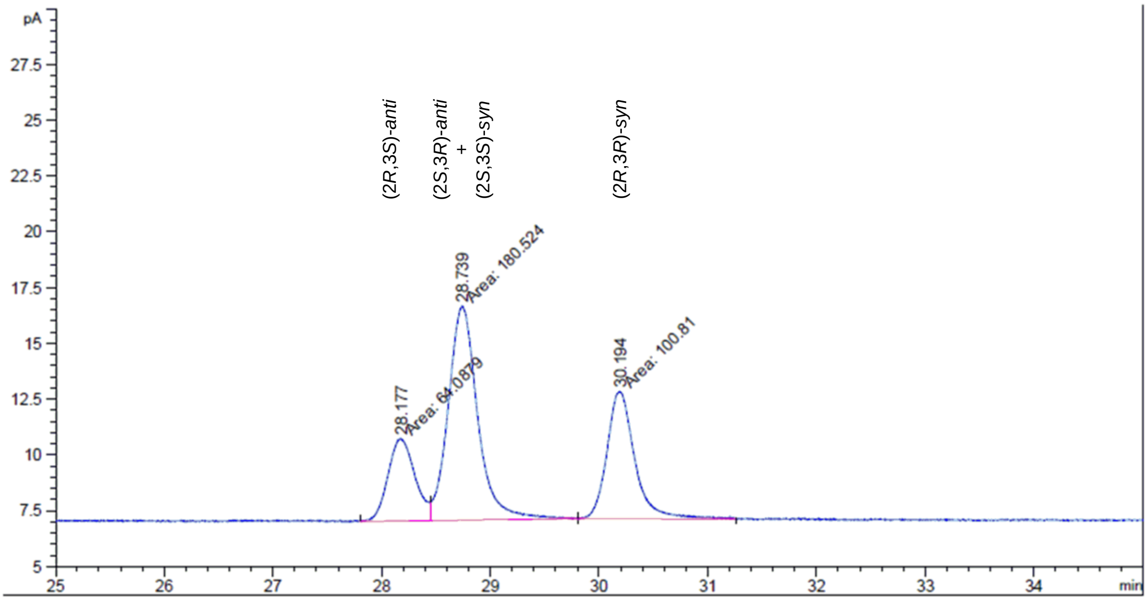


GC obtained from the epoxidation of the allylic alcohol **3b** with *m*CPBA (epoxy alcohol region):

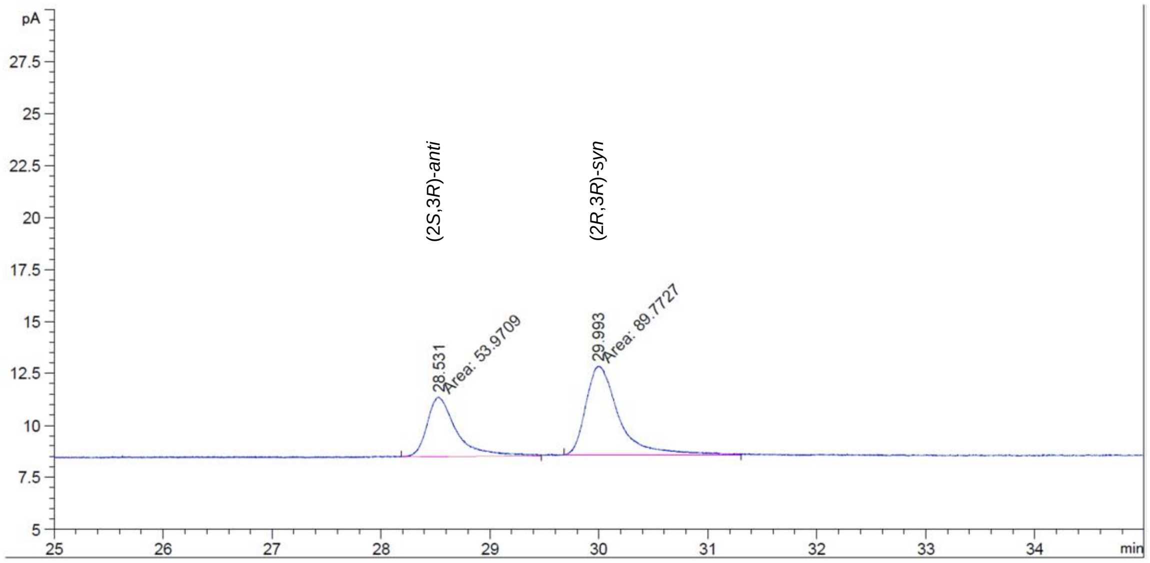

GC obtained from the epoxidation of the allylic alcohol **3b** with the titanium salalen catalyst **2** (epoxy alcohol region):

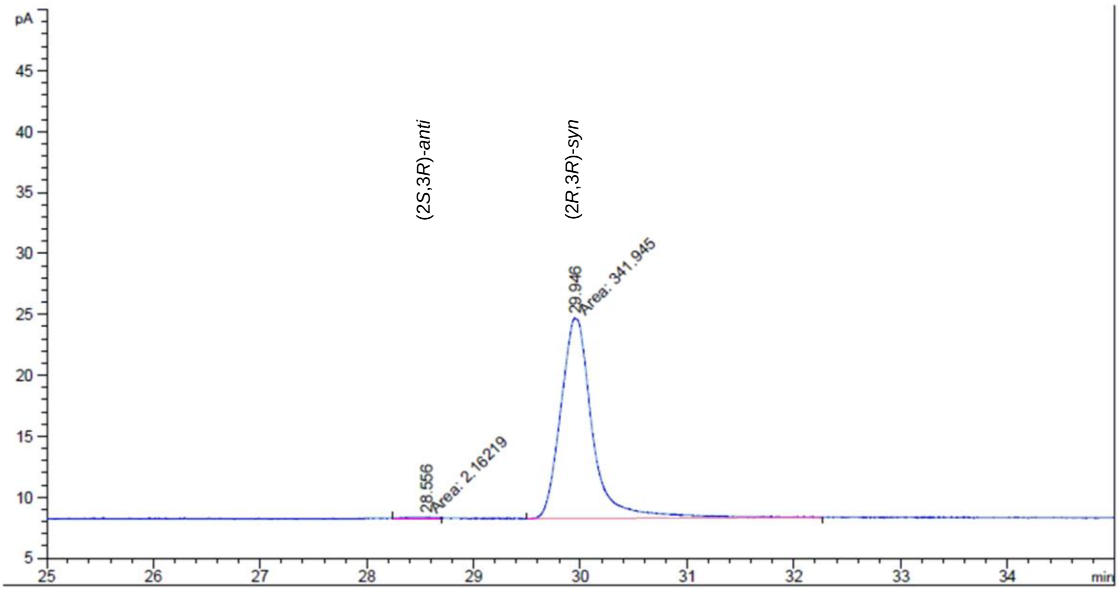


| Structure | $\tau_R$ | Chromatography and Method |
|---|---|---|
| 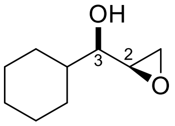 (2*R*,3*R*)-**4c** 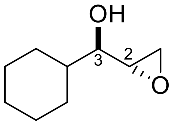 (2*S*,3*R*)-**4c** | $\tau_R$ = 43.3 min (2*S*,3*S*)-**4c**; $\tau_R$ = 47.1 min (2*R*,3*S*)- **4c** and (2*R*,3*R*)- **4c**; $\tau_R$ = 51.1 min (2*S*,3*R*)- **4c**. | GC, Chirasil-Dex CB split = 80:1; split flow = 125.3 mL/min, $N_2$; flow 1.6 mL/min; 100 °C isothermal 55 min, 10 °C/min to 180 °C isothermal 5 min. |

GC obtained from the epoxidation of the allylic alcohol *rac*-**3c** with *m*CPBA (epoxy alcohol region):

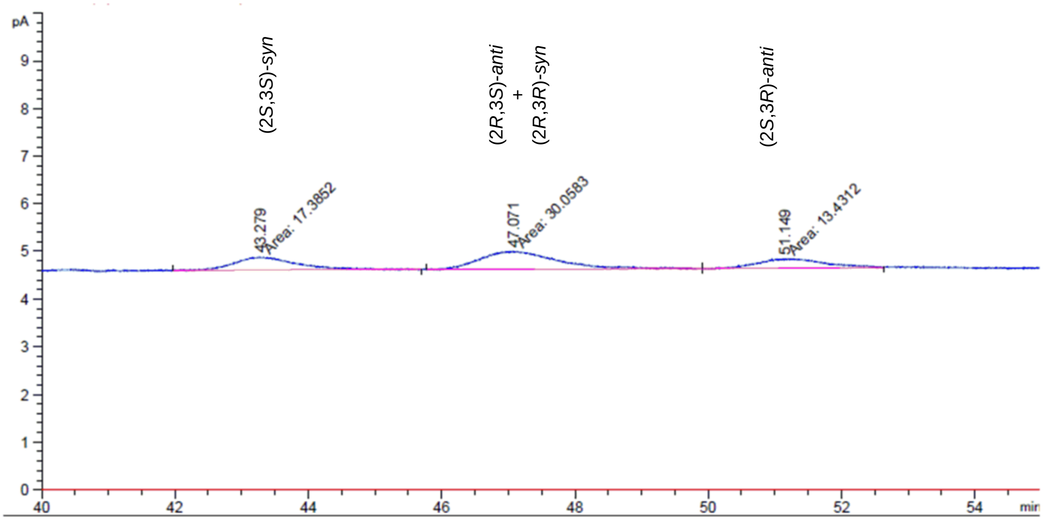

GC obtained from the epoxidation of the allylic alcohol **3c** with *m*CPBA (epoxy alcohol region):

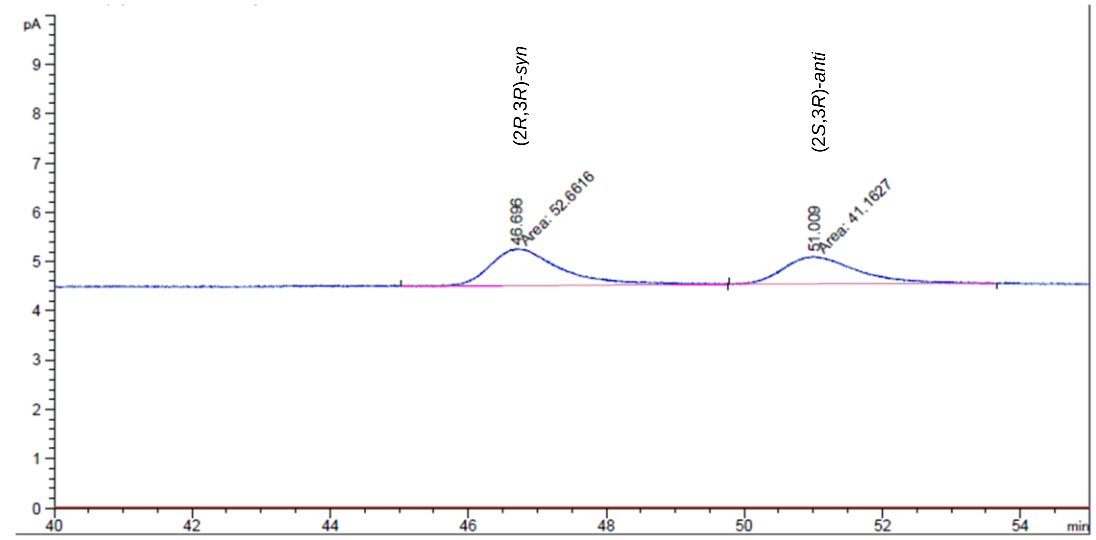


GC obtained from the epoxidation of the allylic alcohol **3c** with the titanium salalen catalyst **2** (epoxy alcohol region):

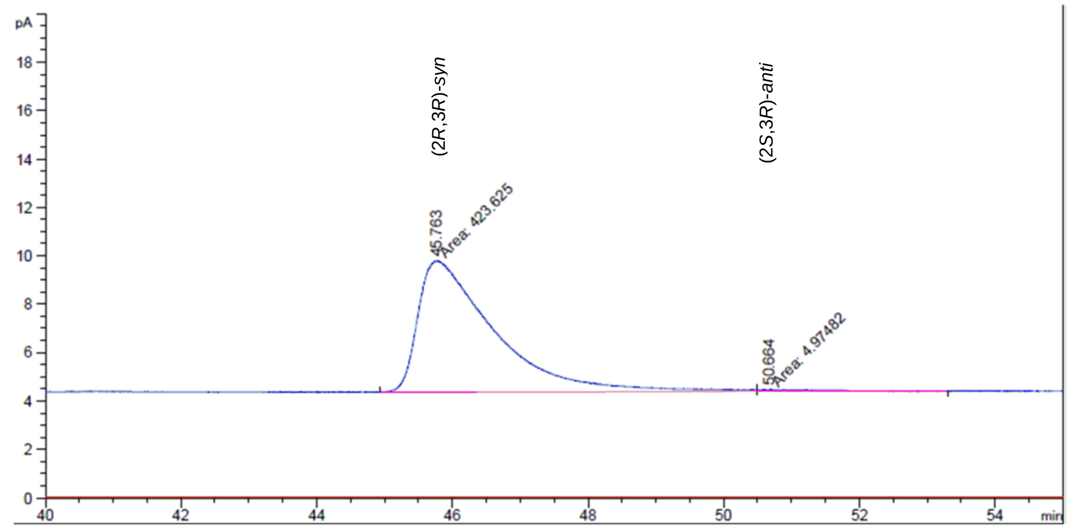


GC obtained from the crystal of the epoxy alcohol **4c** used for X-ray crystallography (epoxy alcohol region):

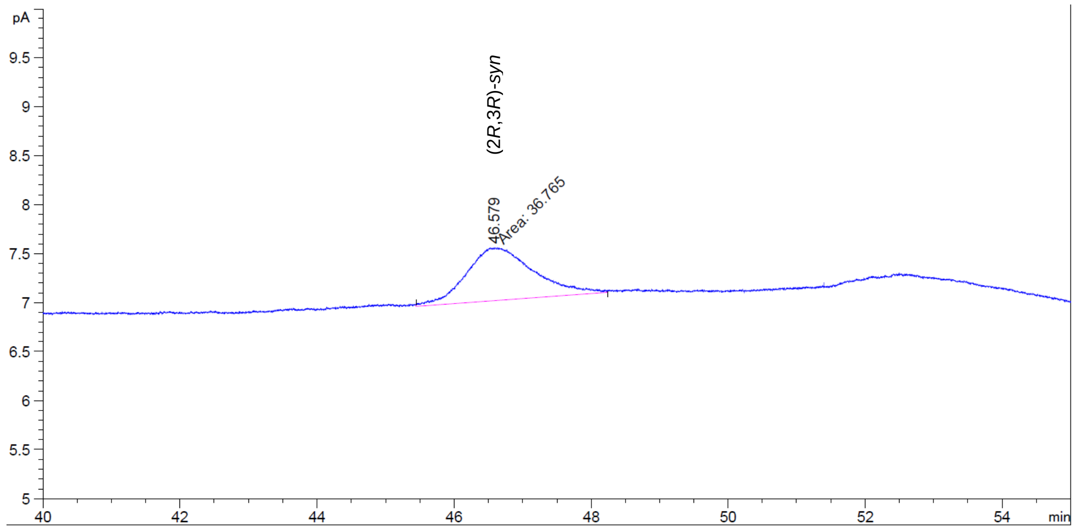

| Structure | $\tau_R$ | Chromatography and Method |
|---|---|---|
| 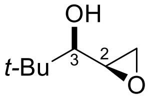 (2*R*,3*R*)-**4d** <br> 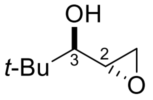 (2*S*,3*R*)-**4d** | $\tau_R$ = 16.4 min (2*S*,3*S*)-**4d**; <br> $\tau_R$ = 17.8 min (2*R*,3*R*)-**4d**; <br> $\tau_R$ = 19.8 min *anti*-**4d**; <br> $\tau_R$ = 21.6 min *anti*-**4d.** | GC, Chirasil-Dex CB split = 80:1; split flow = 100.2 mL/min, $N_2$; flow 1.3 mL/min; 80 °C isothermal 30 min, 10 °C/min to 100 °C isothermal 25 min, 10 °C/min to 180 °C isothermal 5 min. |

GC obtained from the epoxidation of the allylic alcohol *rac*-**3d** with mCPBA (epoxy alcohol region):

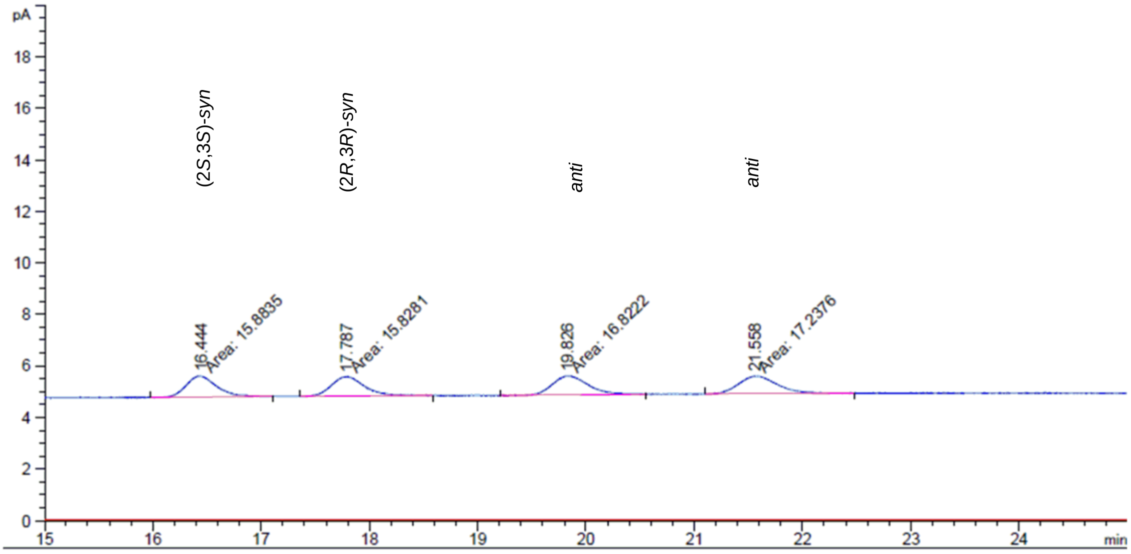


GC obtained from the epoxidation of the allylic alcohol **3d** (98 % *ee*) with the titanium salalen catalyst **2** (epoxy alcohol region):

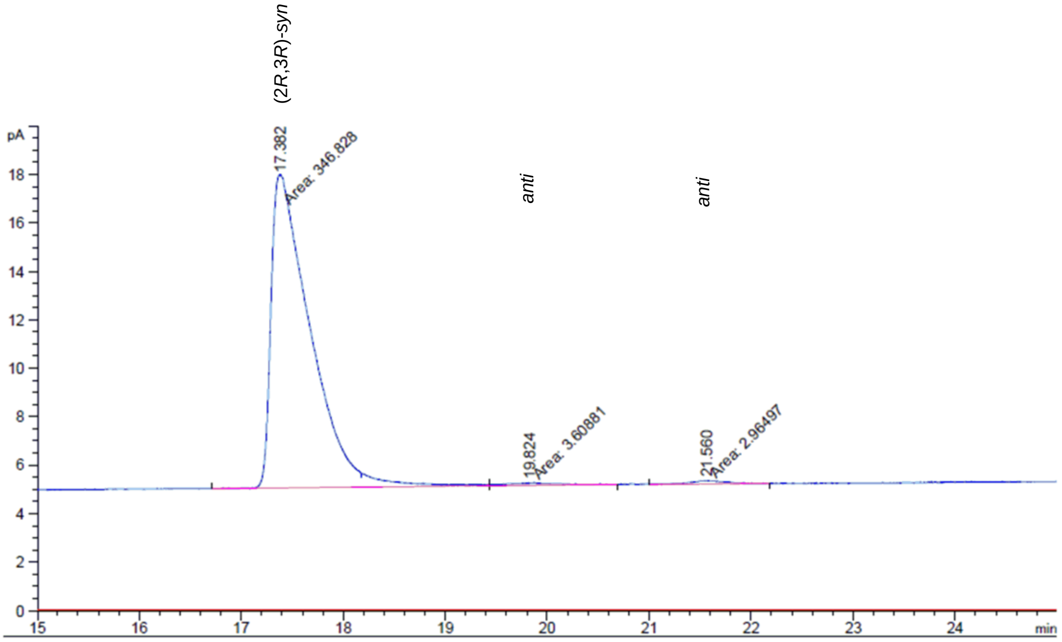

| Structure | $\tau_R$ | Chromatography and Method |
|---|---|---|
| 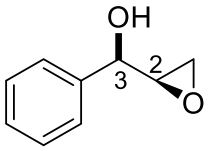 (2*R*,3*R*)-**4e** 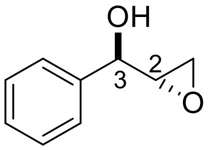 (2*S*,3*R*)-**4e** | $\tau_R$ = 17.1 min ((*S*)-**3e**); $\tau_R$ = 25.4 min ($Ph_2O$); $\tau_R$ = 48.4 min ((2*S*,3*S*)-**4e** or (2*R*,3*S*)-**4e**); $\tau_R$ = 48.6 min ((2*R*,3*S*)-**4e** or (2*S*,3*S*)-**4e**); $\tau_R$ = 48.8 min ((2*S*,3*R*)- **4e**); $\tau_R$ = 49.5 min ((2*R*,3*R*)-**4e**). | Hydrodex β-3-P split = 50:1; split flow = 50 mL/min, $N_2$; flow 1.0 mL/min; 165 °C isothermal 65 min, 10 °C/min to 160 !C isothermal 5 min. |

GC obtained from the epoxidation of the allylic alcohol *rac*-**3e** with mCPBA (epoxy alcohol region):

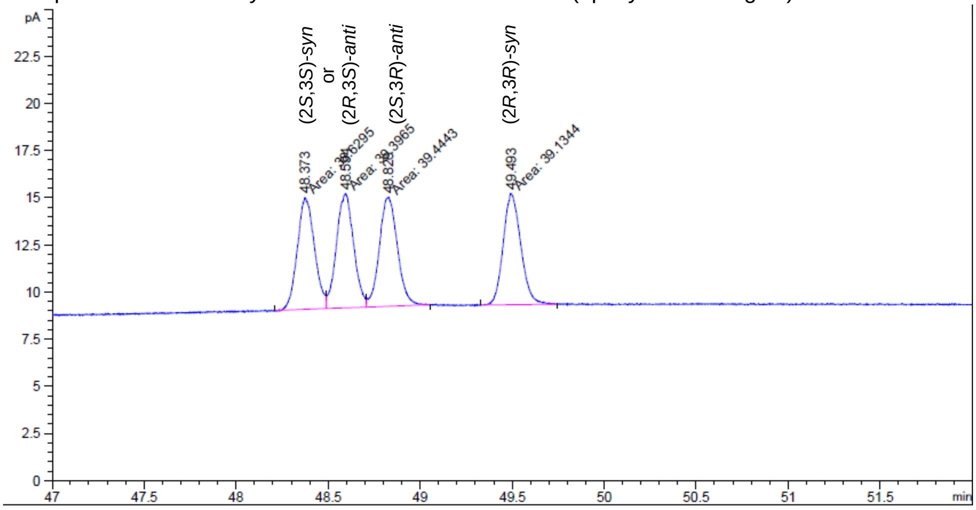


GC obtained from the epoxidation of the allylic alcohol **3e** with mCPBA (epoxy alcohol region):

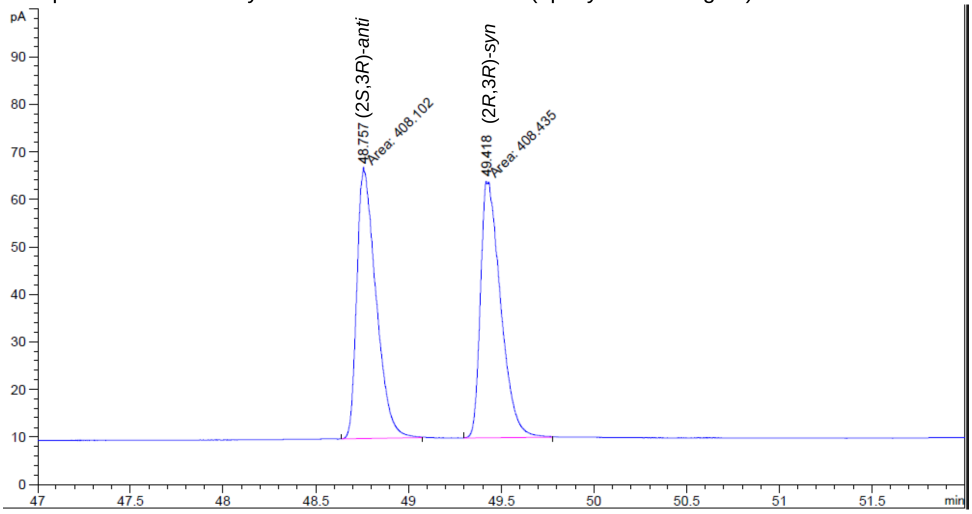

GC obtained from the epoxidation of the allylic alcohol **3e** with the titanium salalen catalyst **2** (epoxy alcohol region):

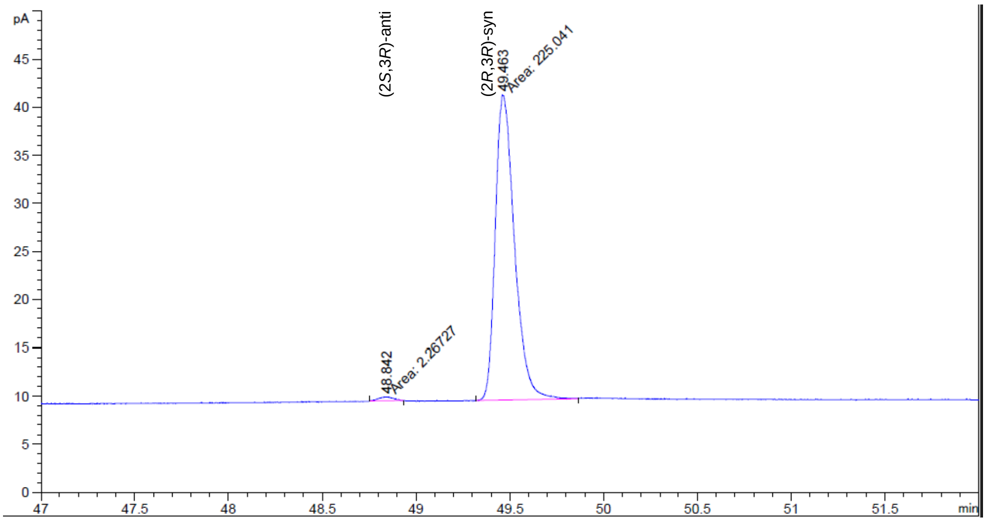


| Structure | $\tau_R$ | Chromatography and Method |
|---|---|---|
| 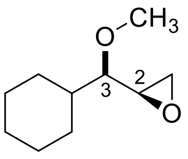<br>(2*R*,3*R*)-**7**<br>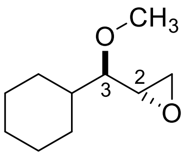<br>(2*S*,3*R*)-**7** | $\tau_R$ = 41.5 min (2*S*,3*R*)-**7;**<br>$\tau_R$ = 44.0 min (2*R*,3*R*)-**7.** | Chirasil-Dex CB<br>split = 80:1; split flow = 97.9 mL/min, $N_2$;<br>flow 1.2 mL/min; 85 °C isothermal<br>50 min, 10 °C/min to 100 °C,<br>Isothermal 16 min,<br>10 °C/min to 180 °C,<br>isothermal 5 min. |

GC obtained from the epoxidation of the methyl ether **6** with *m*CPBA (epoxy ether region):

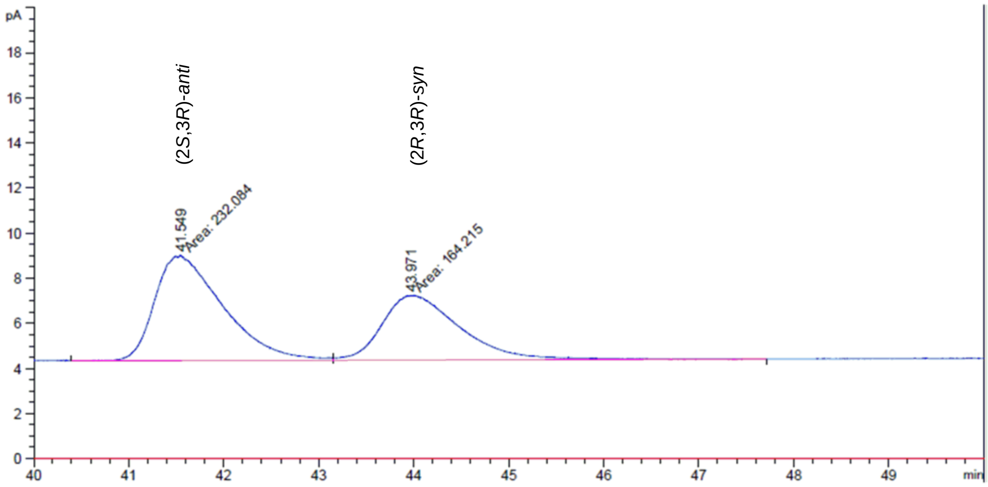

GC obtained from the epoxidation of the methyl ether **6** with the titanium salalen catalyst **2** (epoxy ether region):

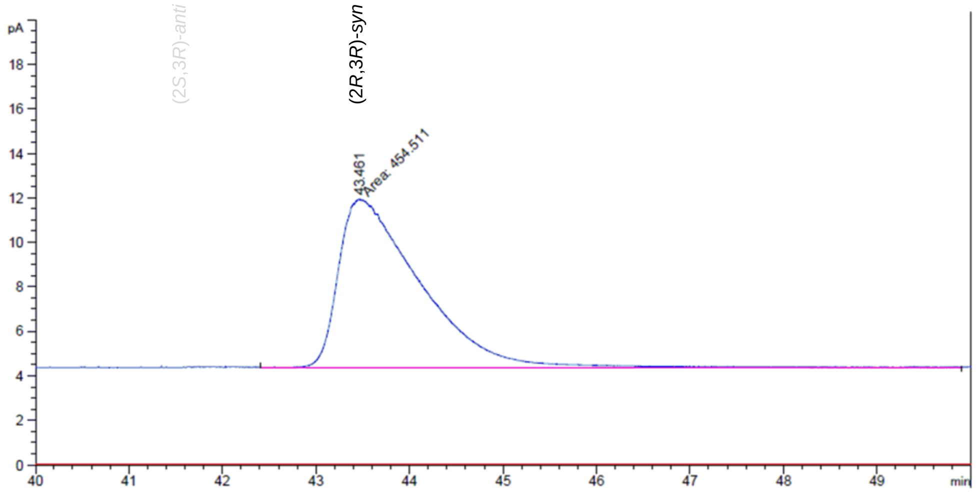


GC of the methyl ether **7** obtained from the methylation of the epoxy alcohol **4c** (epoxy ether region):

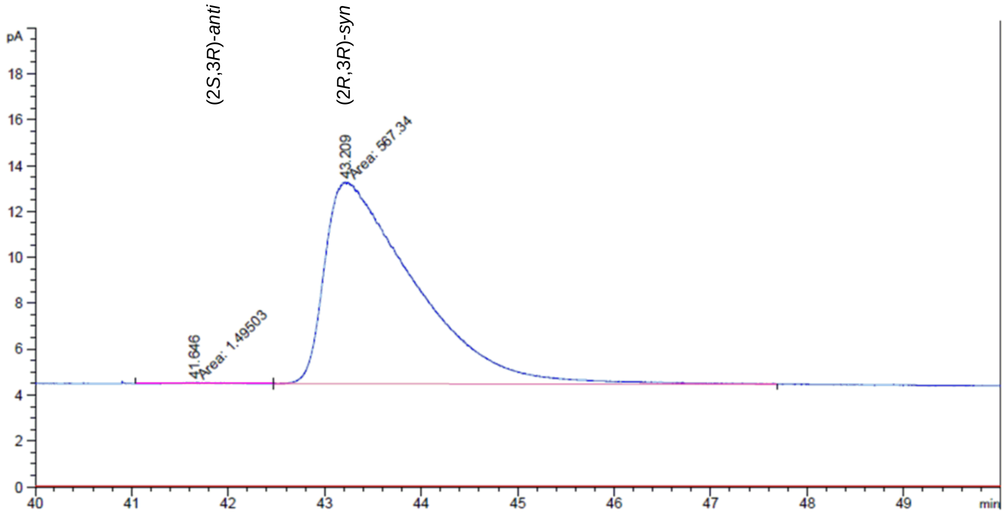

# 4. NMR-Spectra

## 4.1. Allylic alcohols and related compounde

$^{1}$H-NMR (300 MHz) of *(R)*-undec-1-en-3-ol (**3a**) in $CDCl_3$

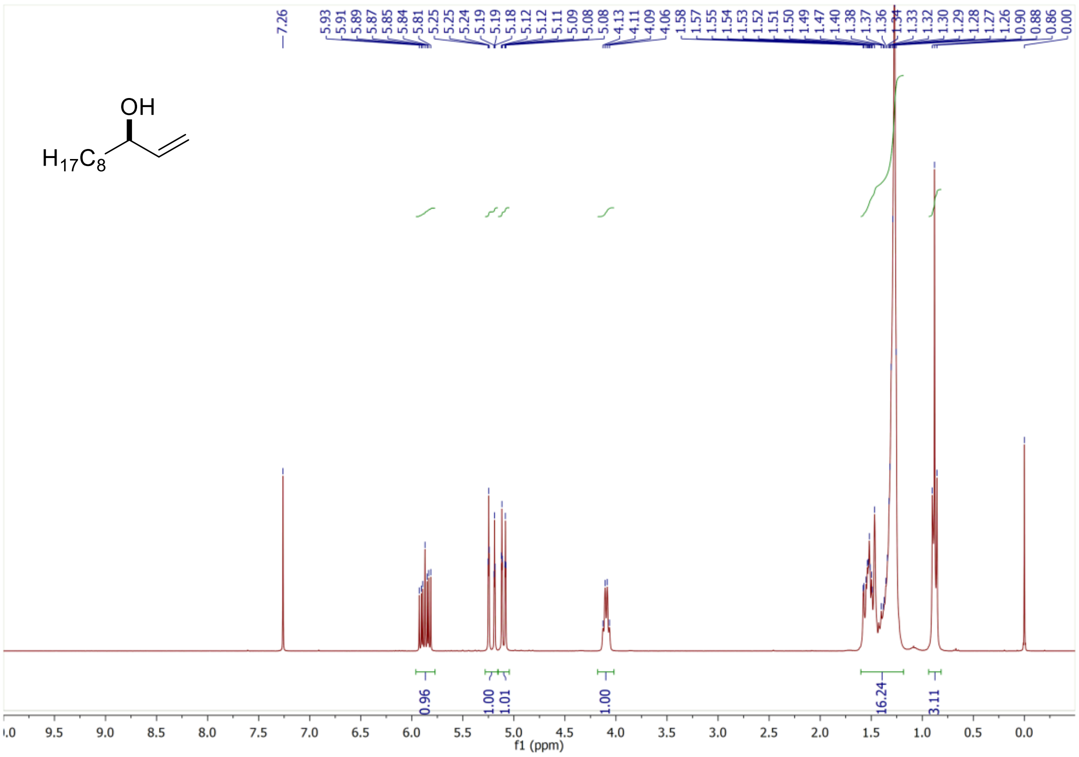

$^{1}$H-NMR (500 MHz) of *(R)*-1-phenylbut-3-en-2-ol (**3b**) in $CDCl_3$

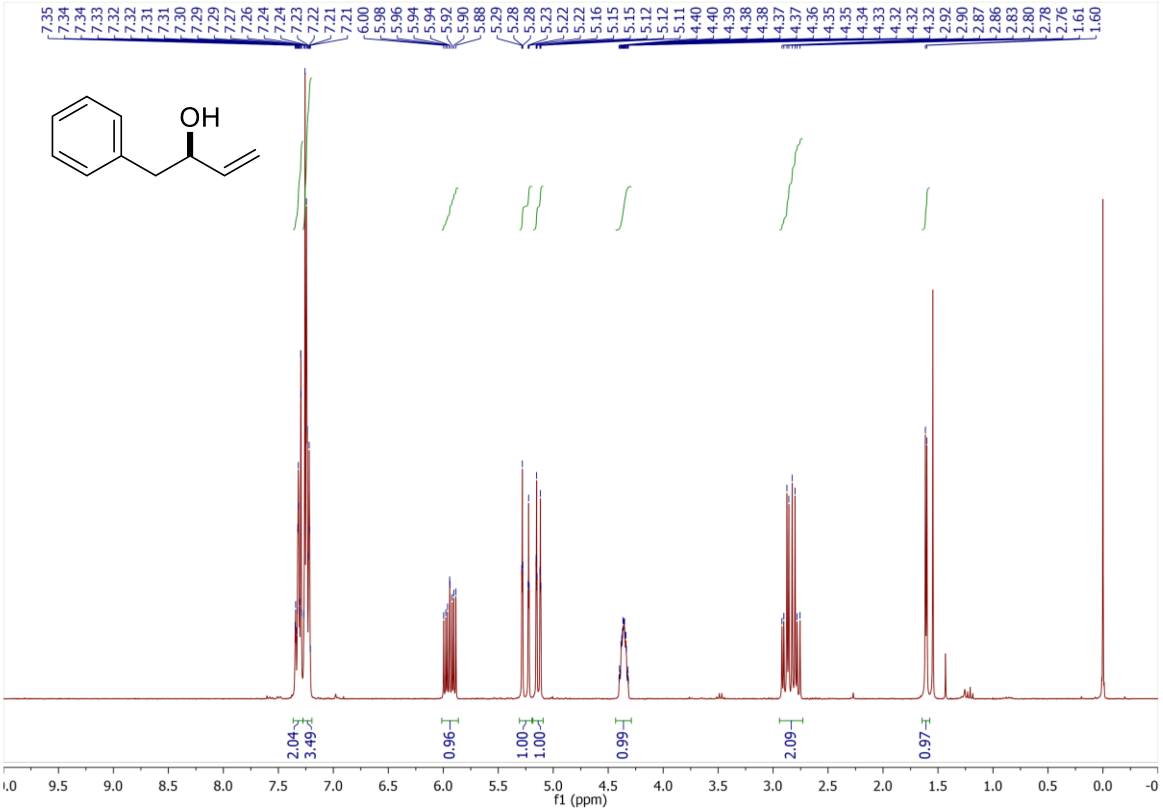

$^1$H-NMR (500 MHz) of *(S)*-1-cyclohexylprop-2-en-1-ol (**3c**) in $CDCl_3$

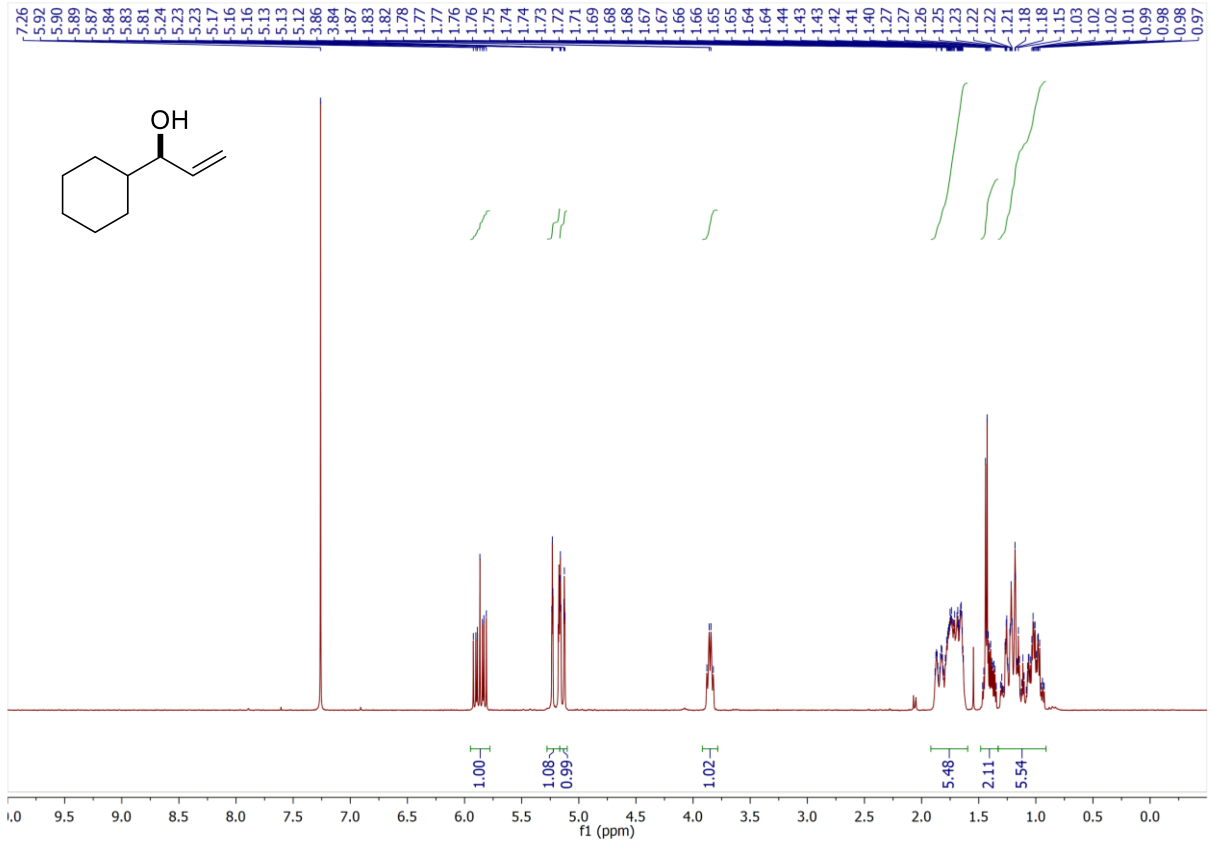

$^1$H-NMR (500 MHz) of *(S)*-4,4-dimethylpent-1-en-3-ol (**3d**) in $CDCl_3$

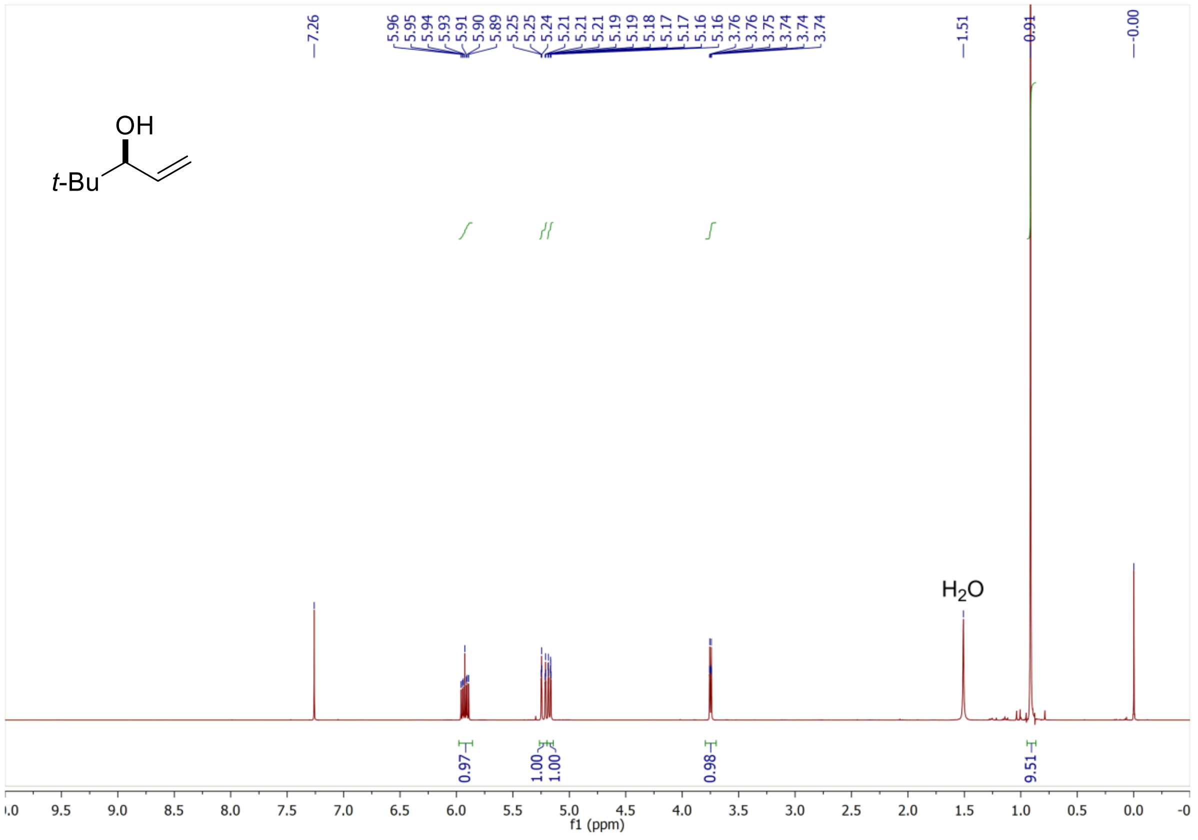

$^{13}$C-NMR (75 MHz) of *(S)*-4,4-dimethylpent-1-en-3-ol (**3d**) in $CDCl_3$

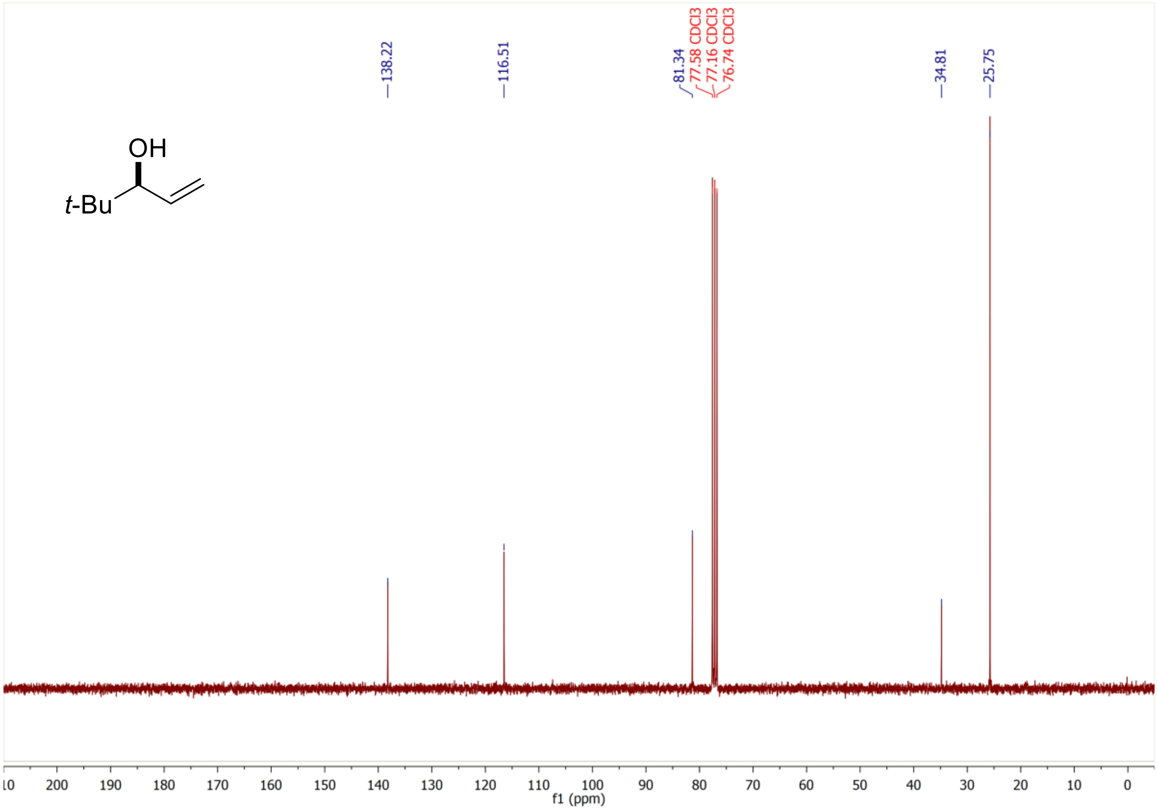


$^{1}$H-NMR (500MHz) of (*S*)-α-vinylbenzyl alcohol (**3e**) in $CDCl_3$

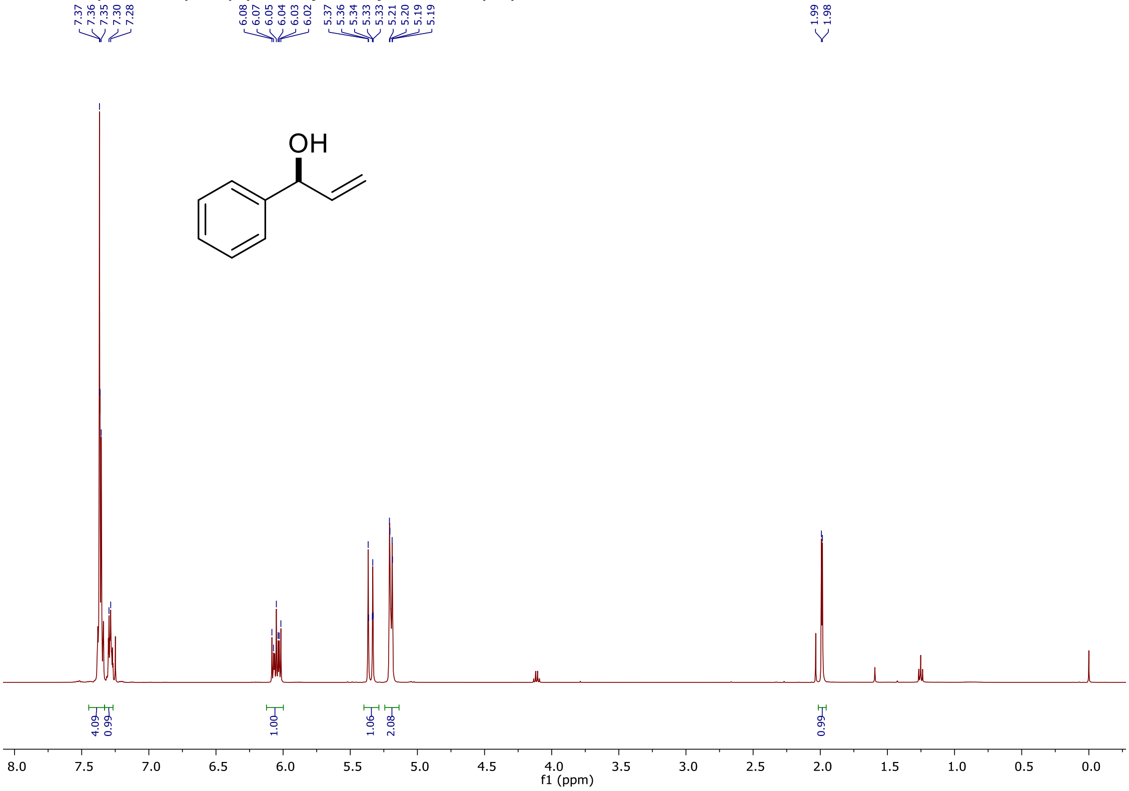

$^{13}$C-NMR (75 MHz) of (*S*)-α-vinylbenzyl alcohol (**3e**) in $CDCl_3$

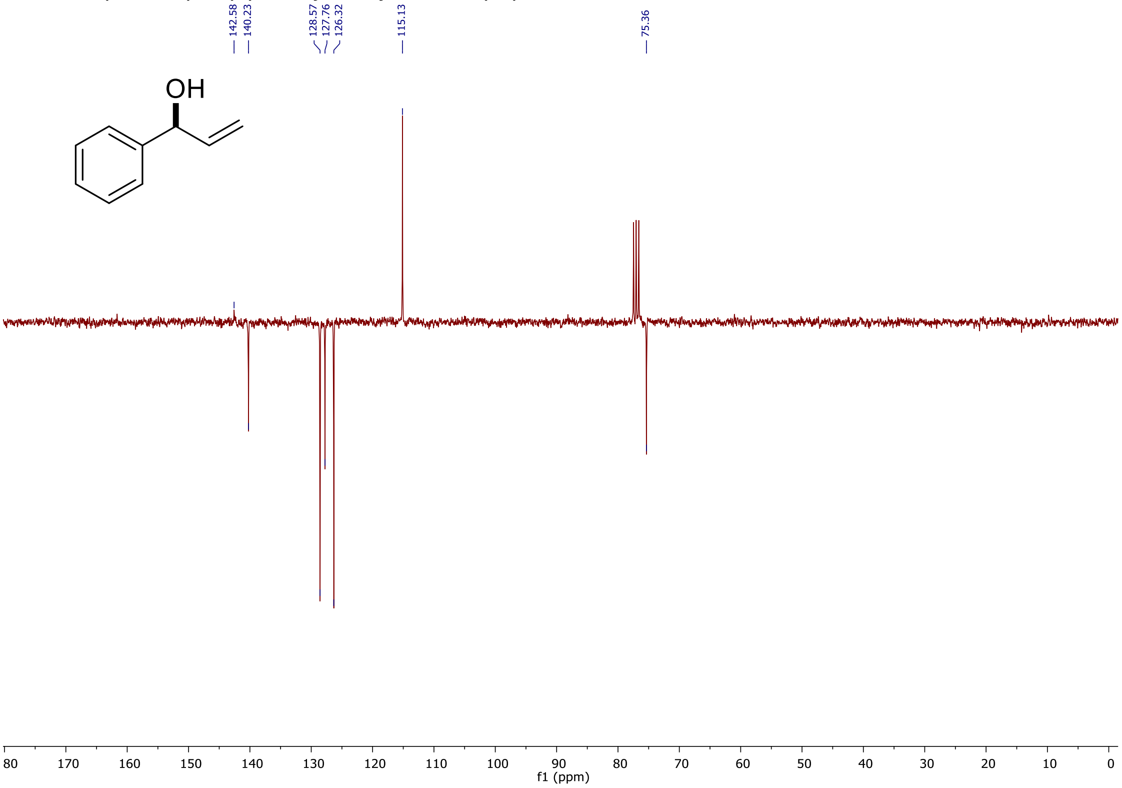


$^{1}$H-NMR (300 MHz) of *(R)*-pentadec-1-en-3-ol (**3f**) in $CDCl_3$

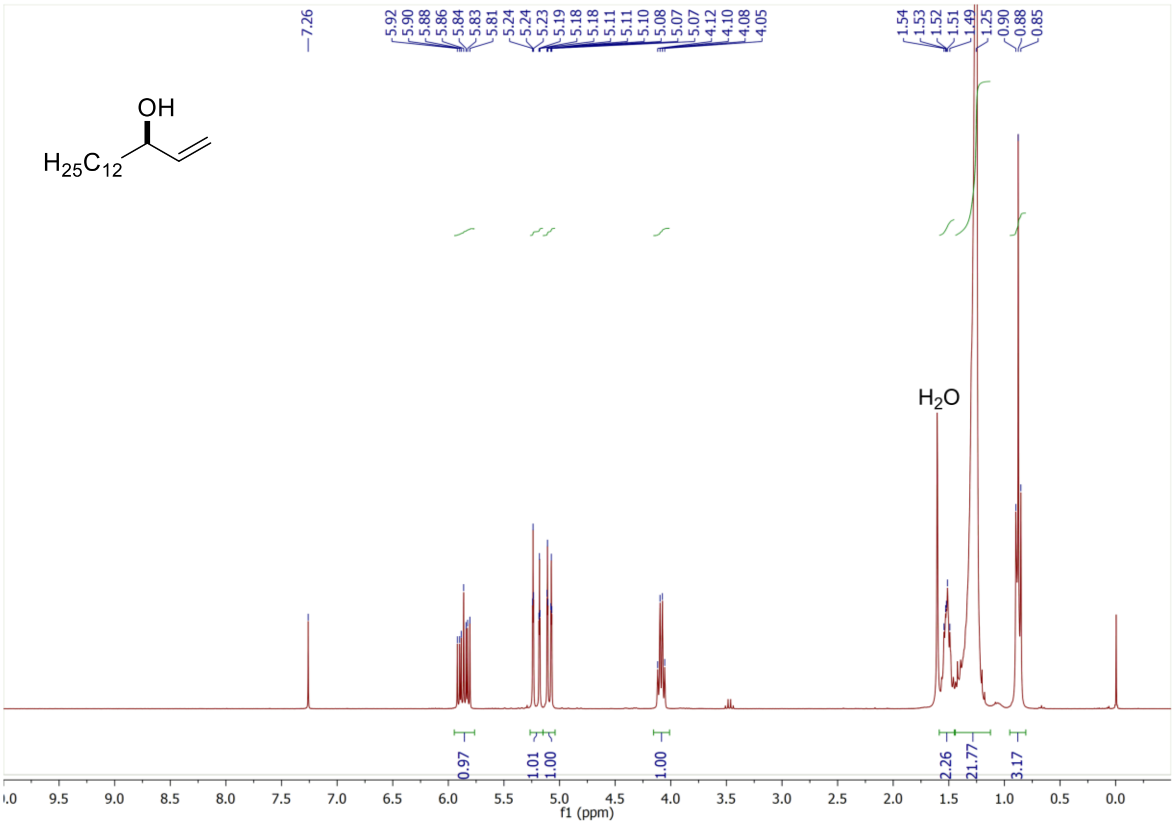

$^{13}$C-NMR (75 MHz) of *(R)*-pentadec-1-en-3-ol (**3f**) in $CDCl_3$

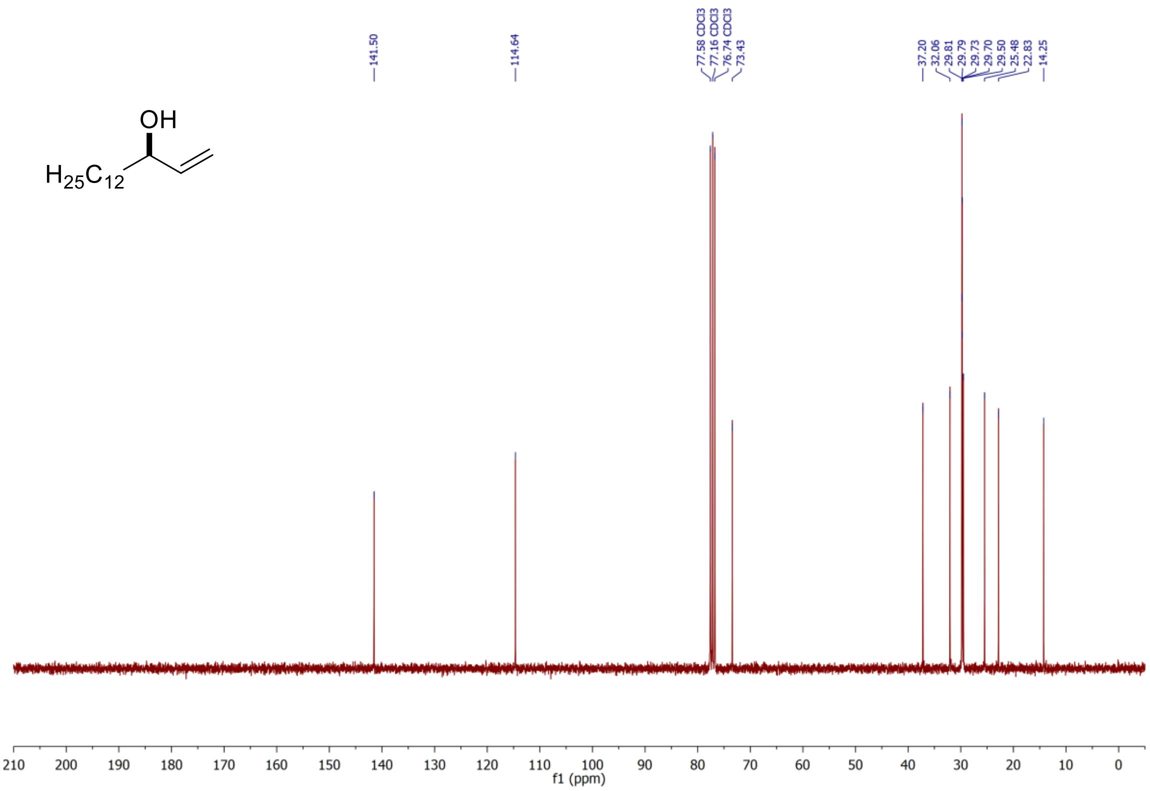


$^{1}$H-NMR (500 MHz) of *(S)*-(3-methoxyallyl)cyclohexane (**6**) in $CDCl_3$

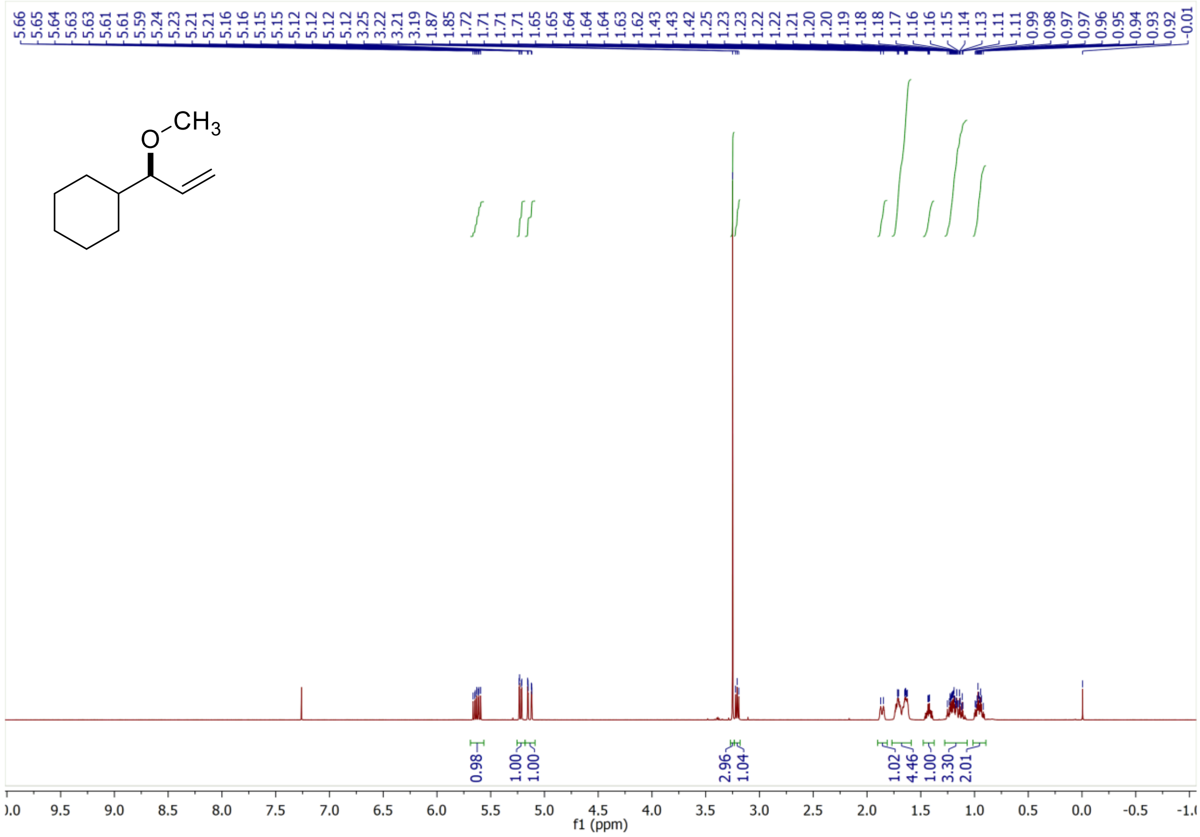

$^{13}$C-NMR (125 MHz) of *(S)*-(3-methoxyallyl)cyclohexane (**6**) in $CDCl_3$

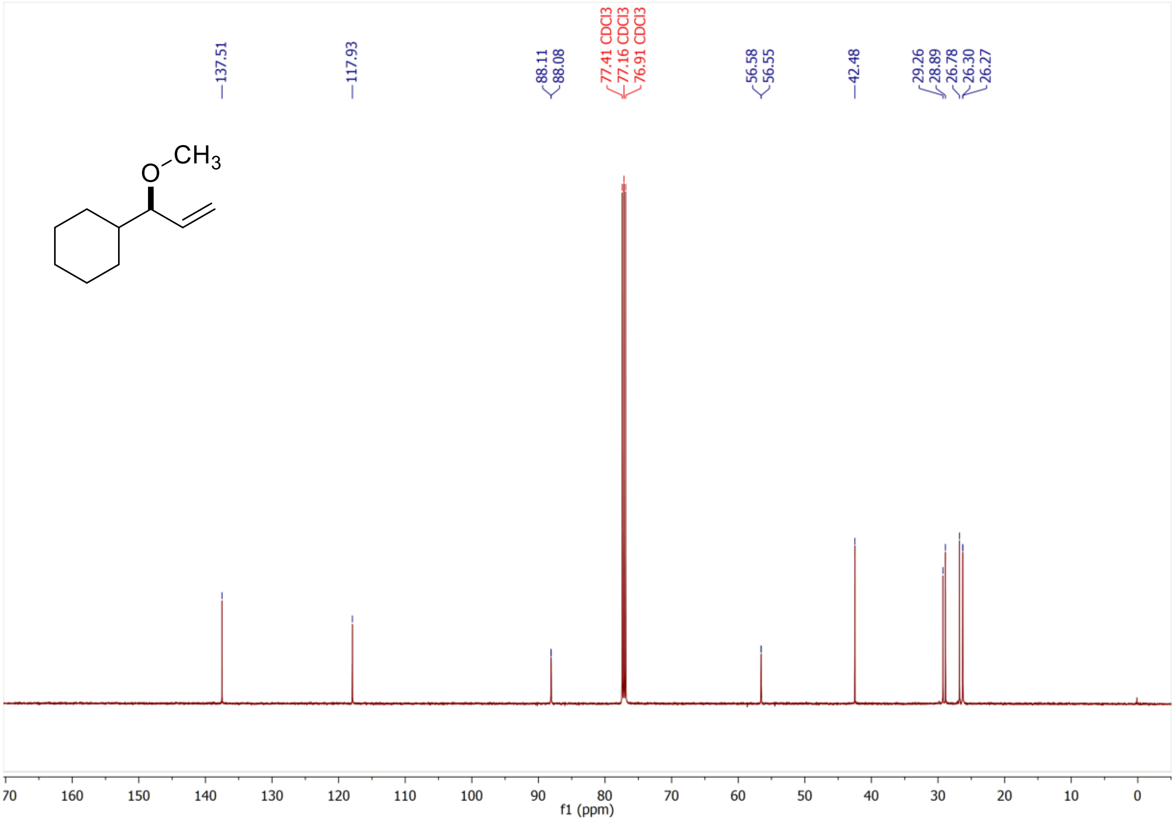


$^{1}$H-NMR (500 MHz) of pentadec-1-en-3-yl acetate (*ent*-**3f-Ac**) in $CDCl_3$

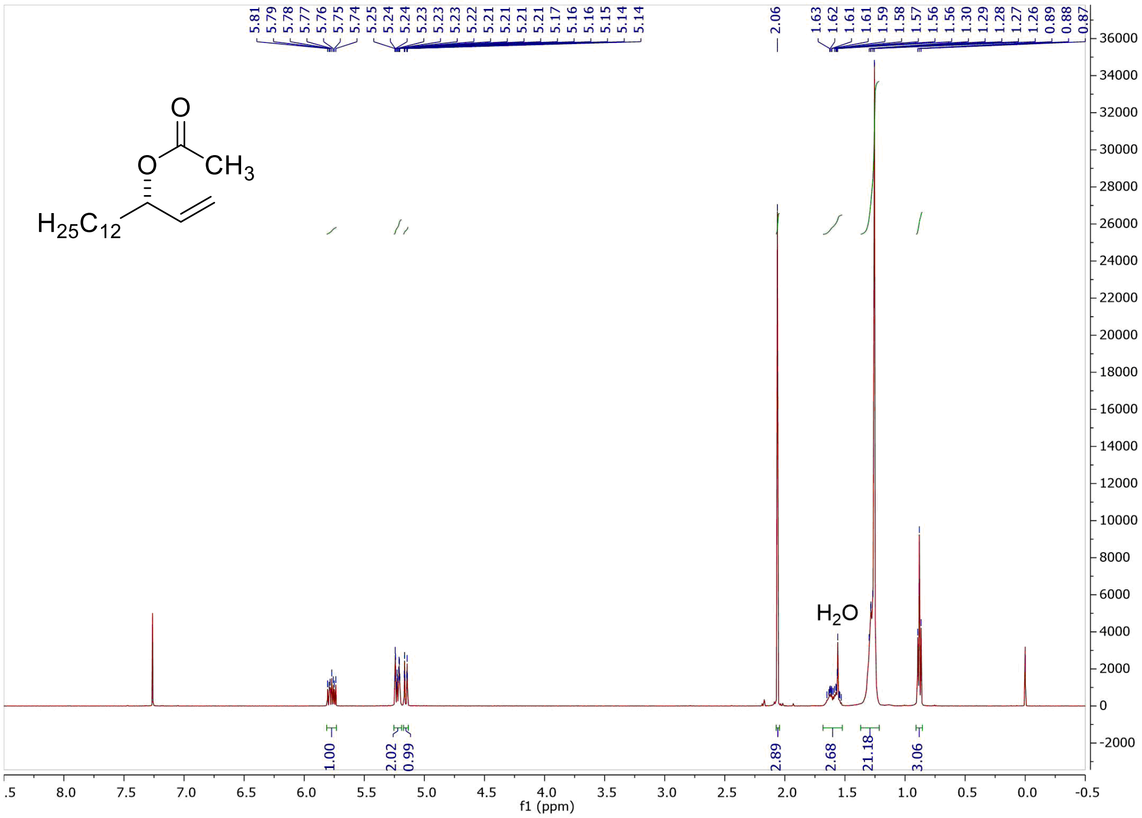

$^{13}$C-NMR (125 MHz) of pentadec-1-en-3-yl acetate (*ent*-**3f-Ac**) in $CDCl_3$

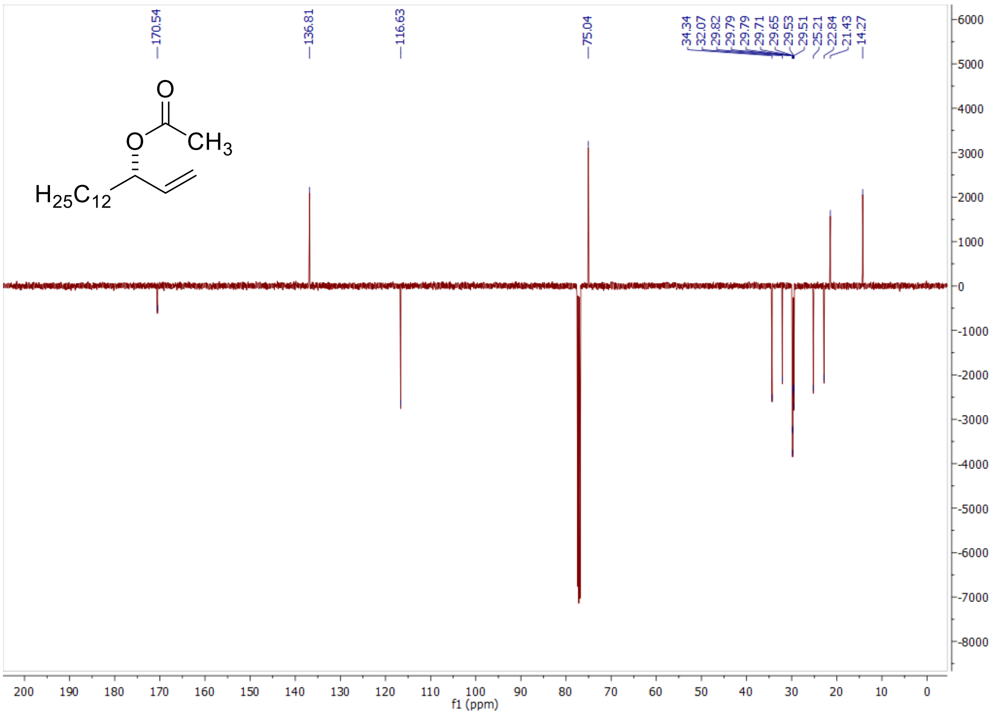

### 4.2. *syn/anti*-Epoxy alcohols obtained from mCPBA epoxidation

$^{1}$H-NMR (300 MHz) in $CDCl_3$, *rac*-undecec-1-en-ol (*rac*-**3a**) as substrate

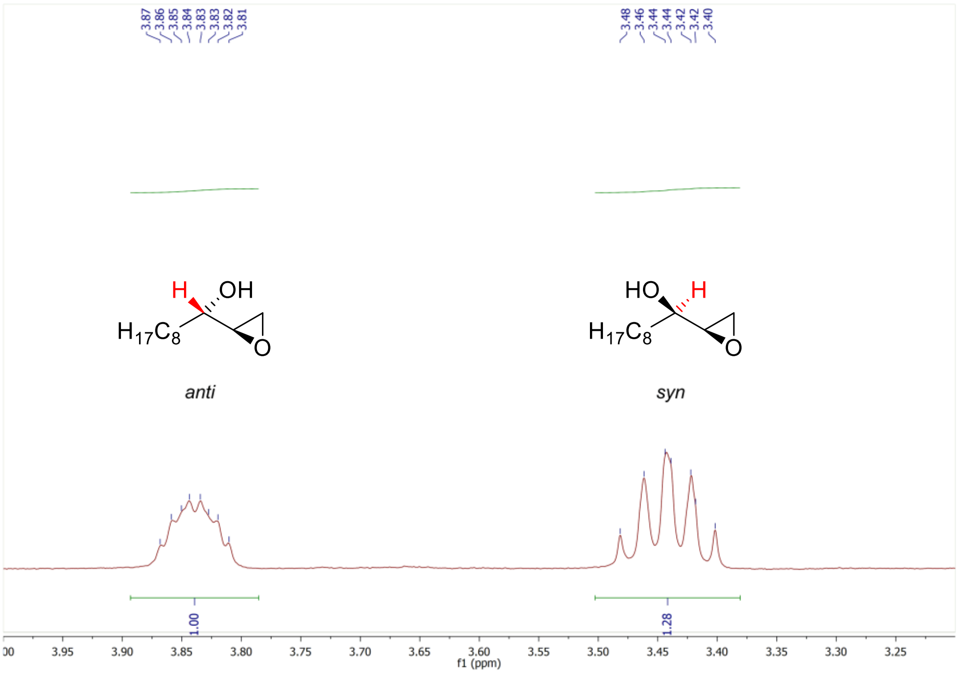

$^{1}$H-NMR (500 MHz) in $CDCl_3$, *rac*-1-phenylbut-3-en-2-ol (*rac*-**3b**) as substrate

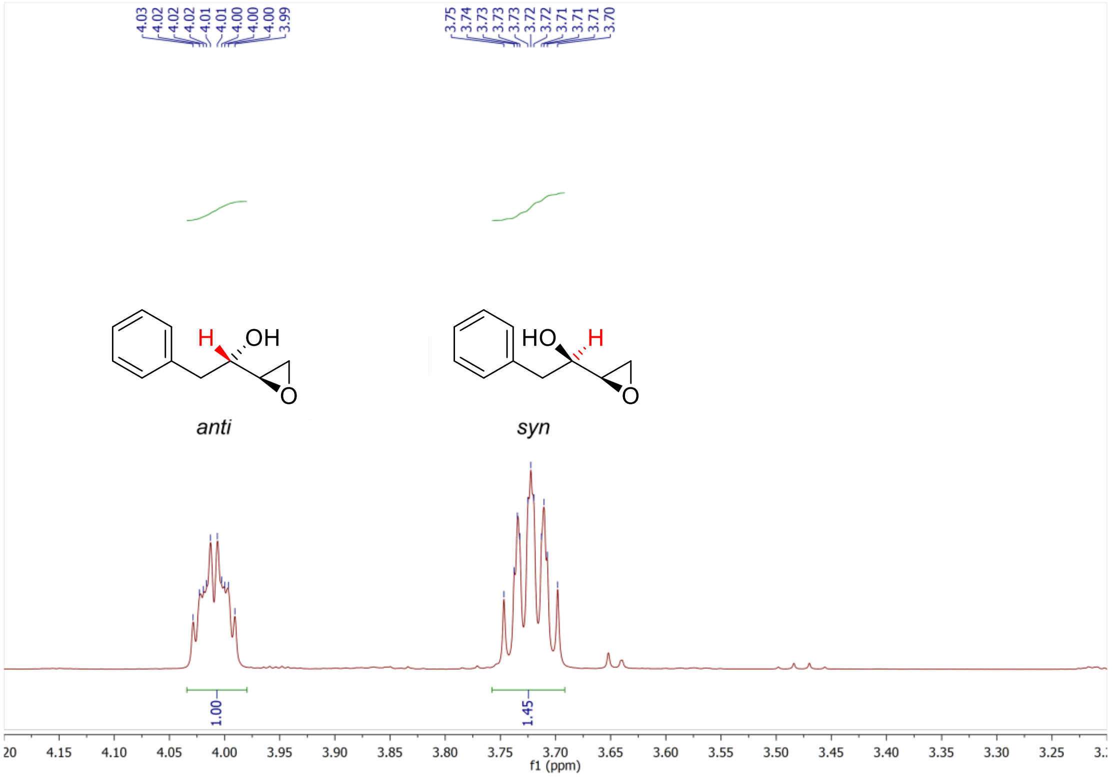


$^{1}$H-NMR (500 MHz) in $CDCl_3$, *rac*-1-cyclohexylprop-2-en-1-ol (*rac*-**3c**) as substrate

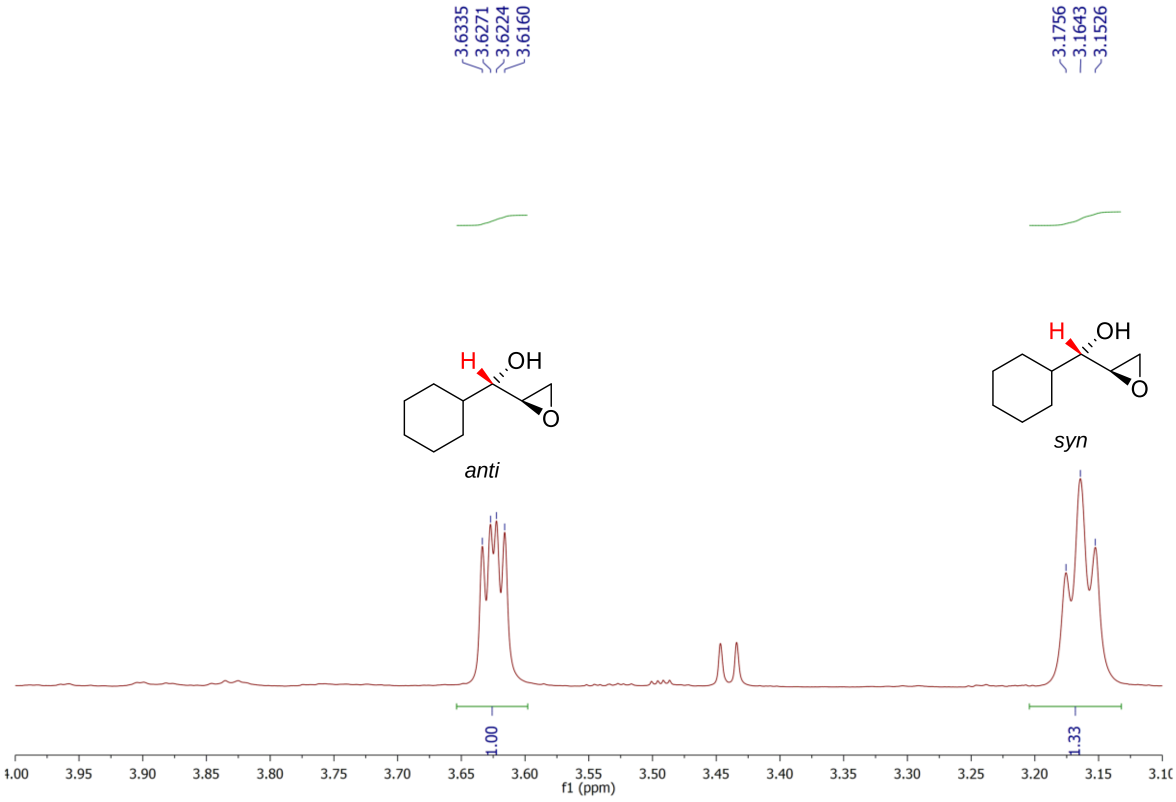

$^{1}$H-NMR (300 MHz) in $CDCl_3$, *rac*-4,4-dimethylpent-1-en-3-ol (*rac*-**3d**) as substrate

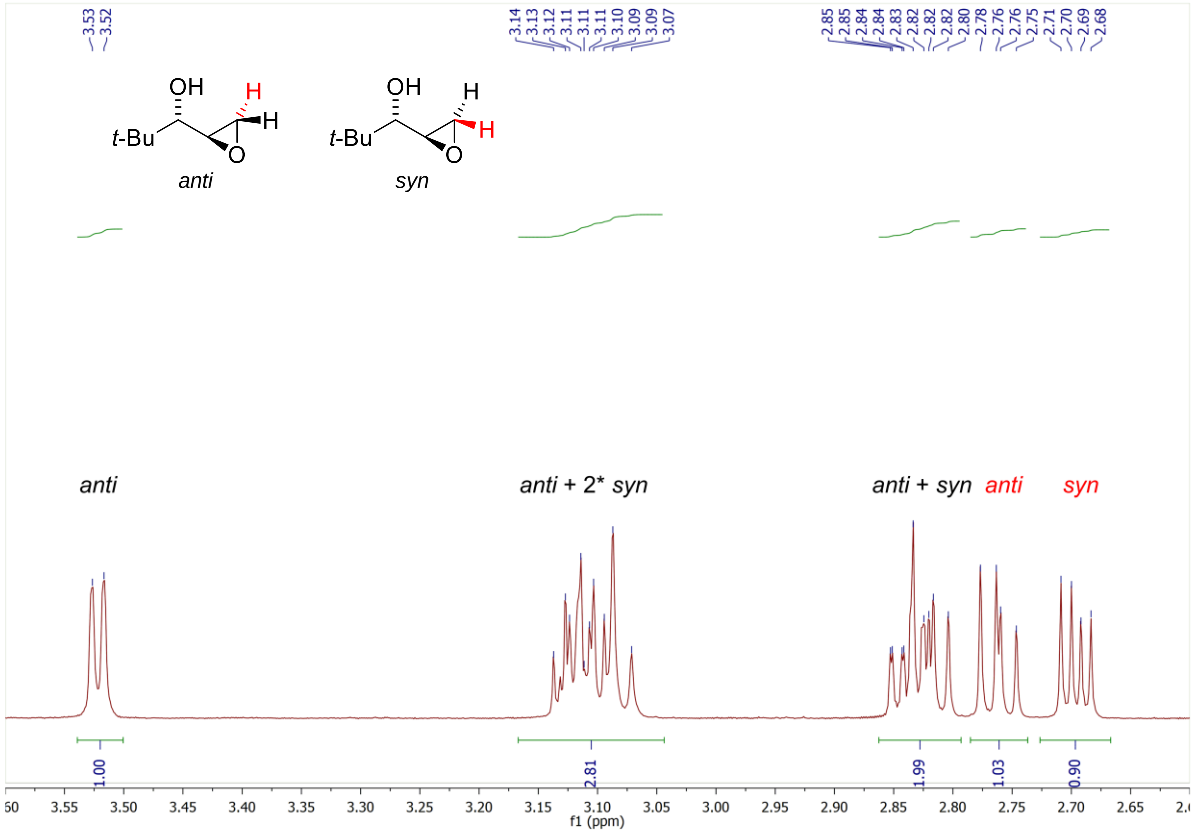


$^{1}$H-NMR (500 MHz) in $CDCl_3$, *rac*-α-vinylbenzyl alcohol (**3e**) as substrate

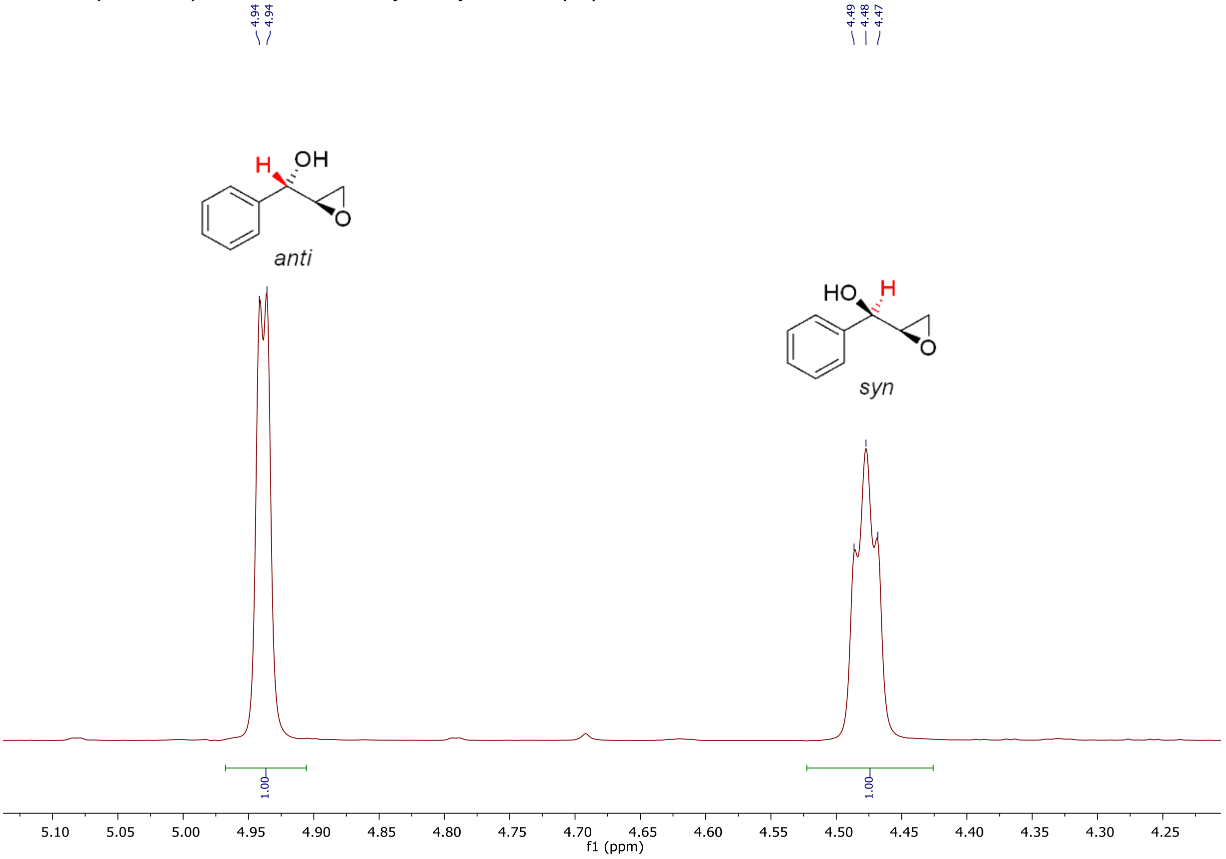

$^1$H-NMR (300 MHz) in $CDCl_3$, *rac*-pentadec-1-en-3-ol (*rac*-**3f**) as substrate

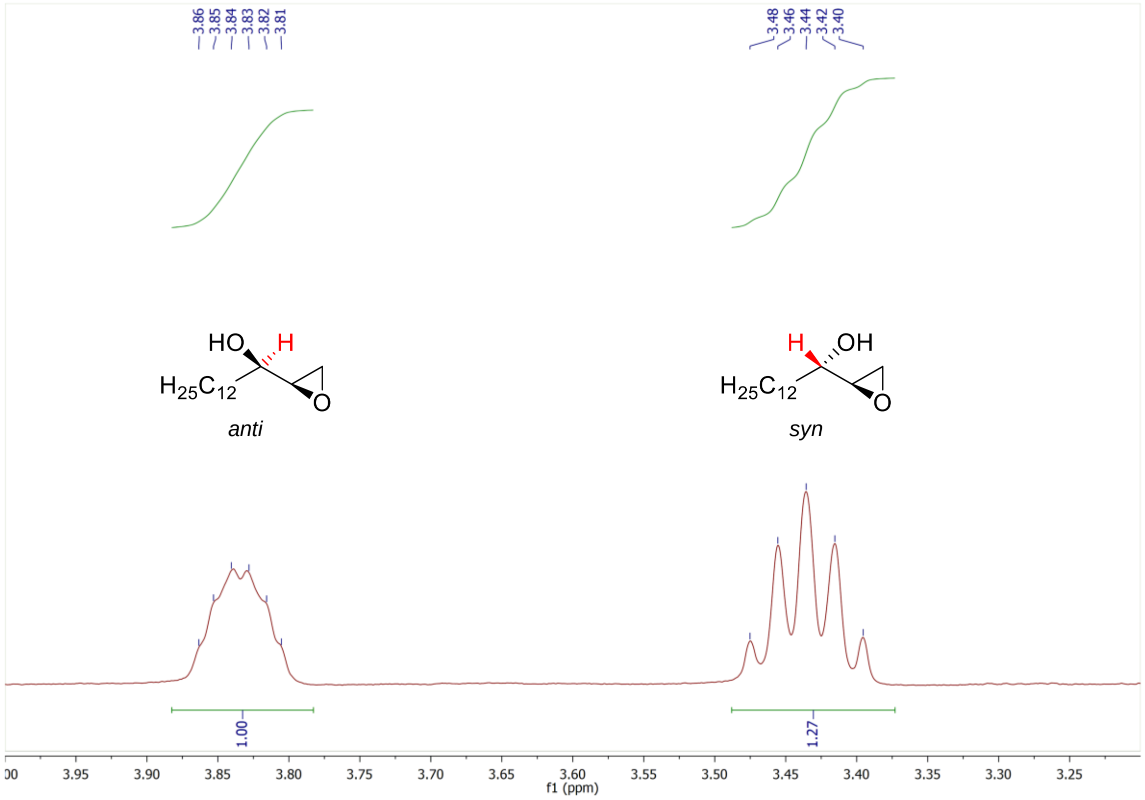

### 4.3. *syn*-Epoxy alcohols obtained by catalytic epoxidation with the titanium salalen catalyst 2

$^{1}H$-NMR (300 MHz) in $CDCl_3$, *(R)*-undecec-1-en-ol (**3a**) as substrate

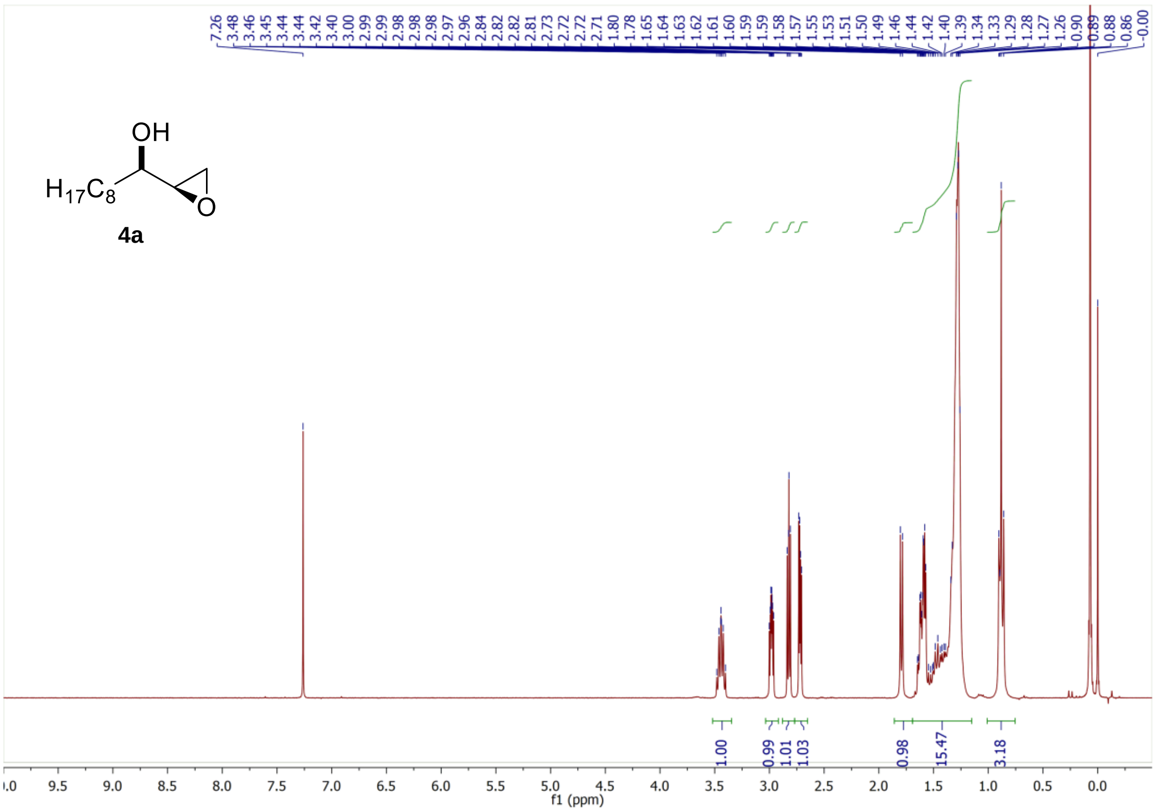


$^{13}C$-NMR (75 MHz) in $CDCl_3$, *(R)*-undecec-1-en-ol (**3a**) as substrate

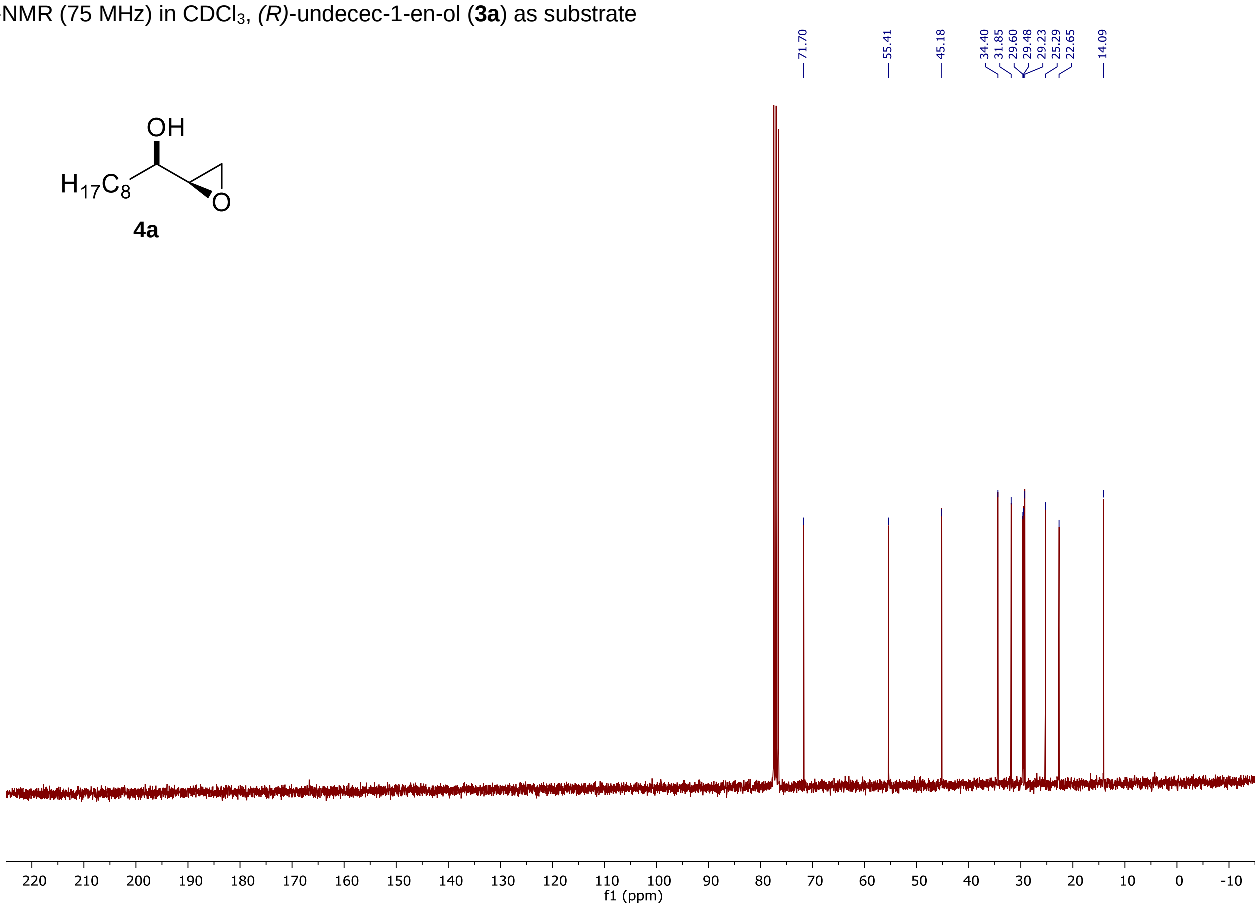

$^{1}$H-NMR (500 MHz) in $CDCl_3$, (*R*)-1-phenylbut-3-en-2-ol (**3b**) as substrate:

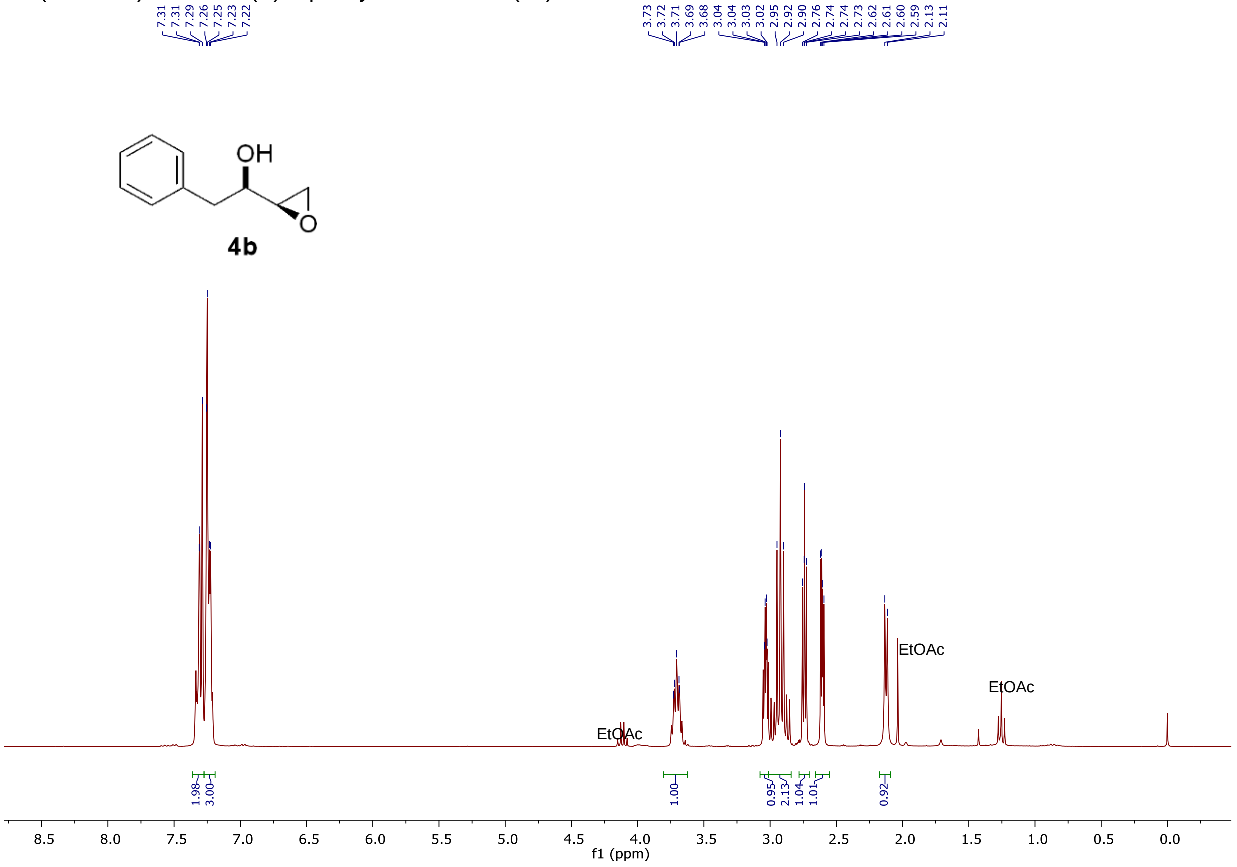


$^{13}$C-NMR (75MHz) in $CDCl_3$, (*R*)-1-phenylbut-3-en-2-ol (**3b**) as substrate:

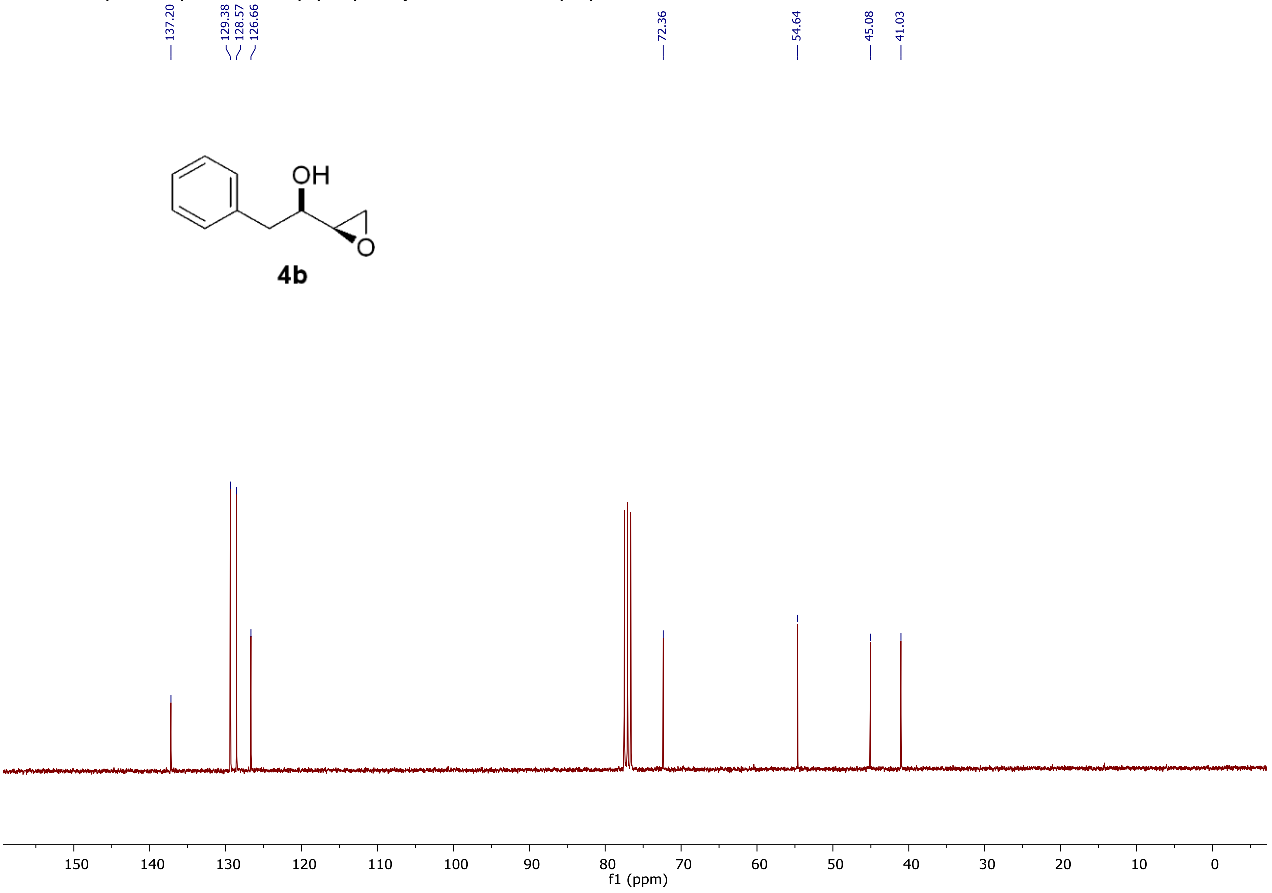

$^{1}$H-NMR (300 MHz) in $CDCl_3$, *(S)*-1-cyclohexylprop-2-en-1-ol (**3c**) as substrate

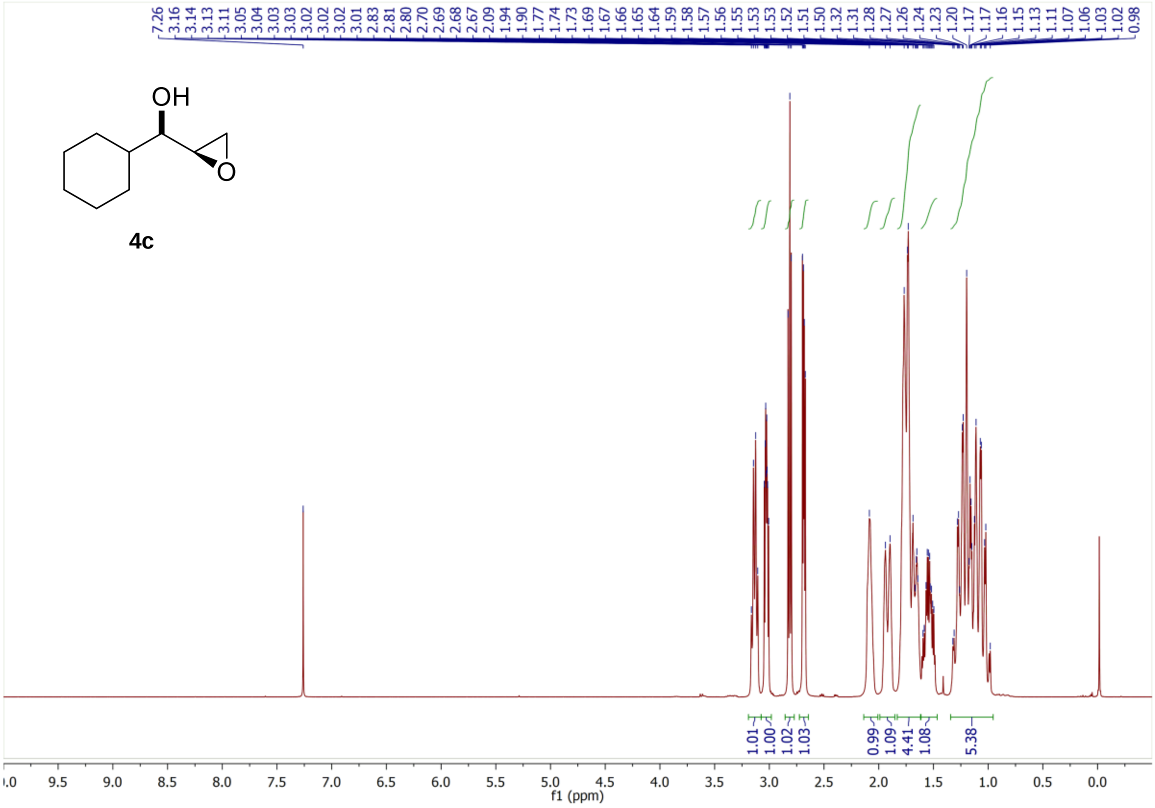


$^{13}$C-NMR (75 MHz) in $CDCl_3$, *(S)*-1-cyclohexylprop-2-en-1-ol (**3c**) as substrate

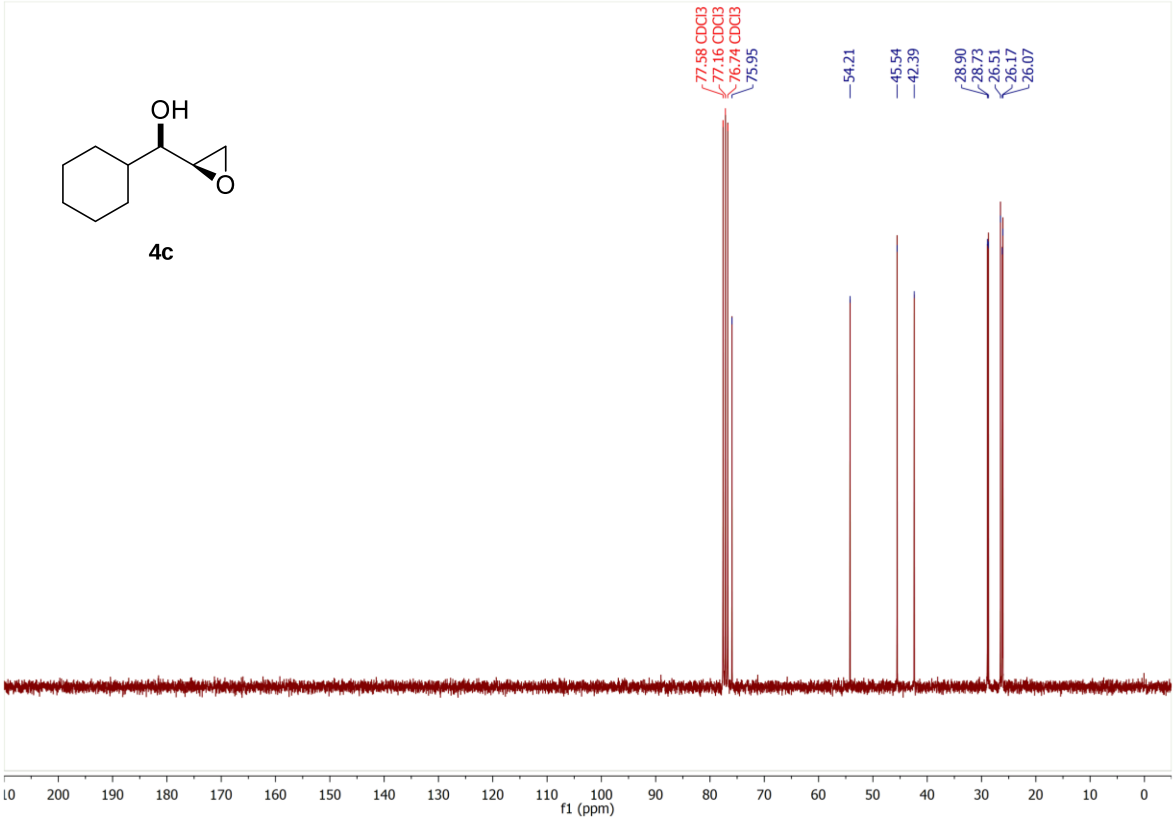

$^{1}$H-NMR (500 MHz) in $CDCl_3$, *(S)*-4,4-dimethylpent-1-en-3-ol (**3d**) as substrate

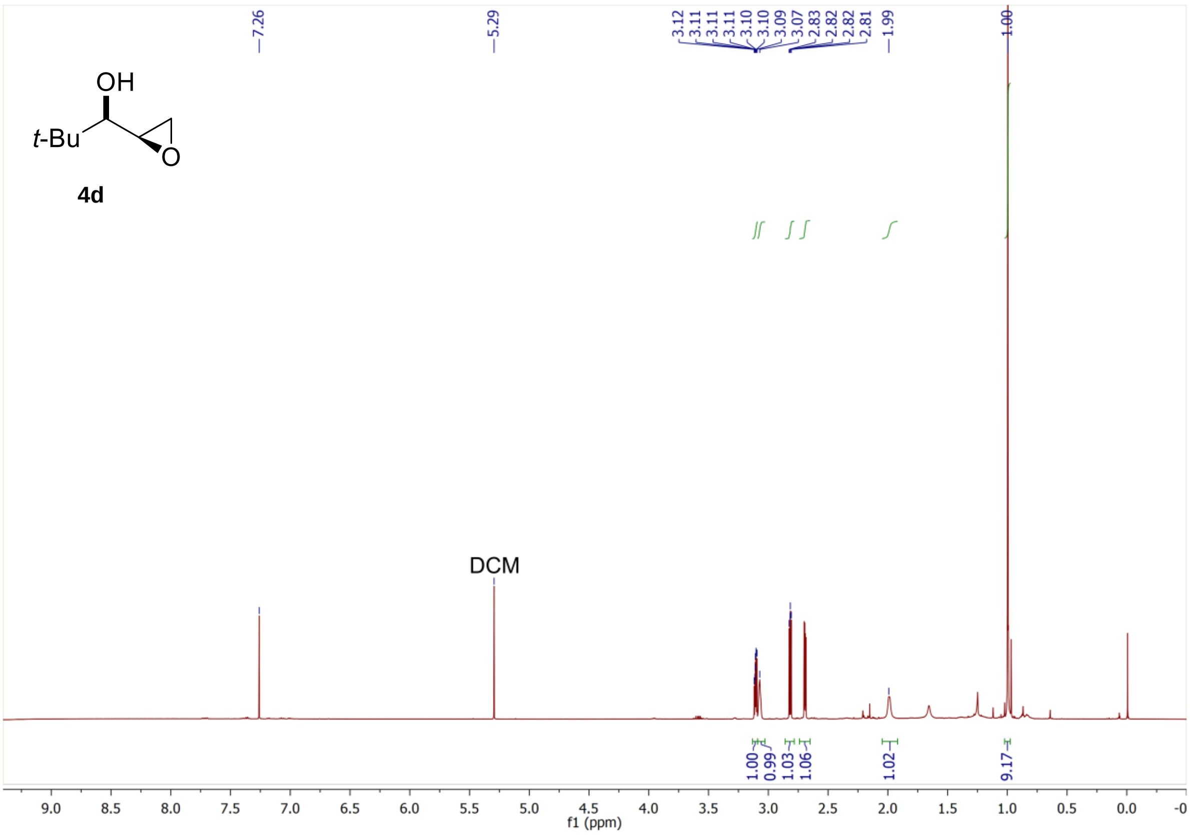


$^{13}$C-NMR (125 MHz) in $CDCl_3$, *(S)*-4,4-dimethylpent-1-en-3-ol (**3d**) as substrate

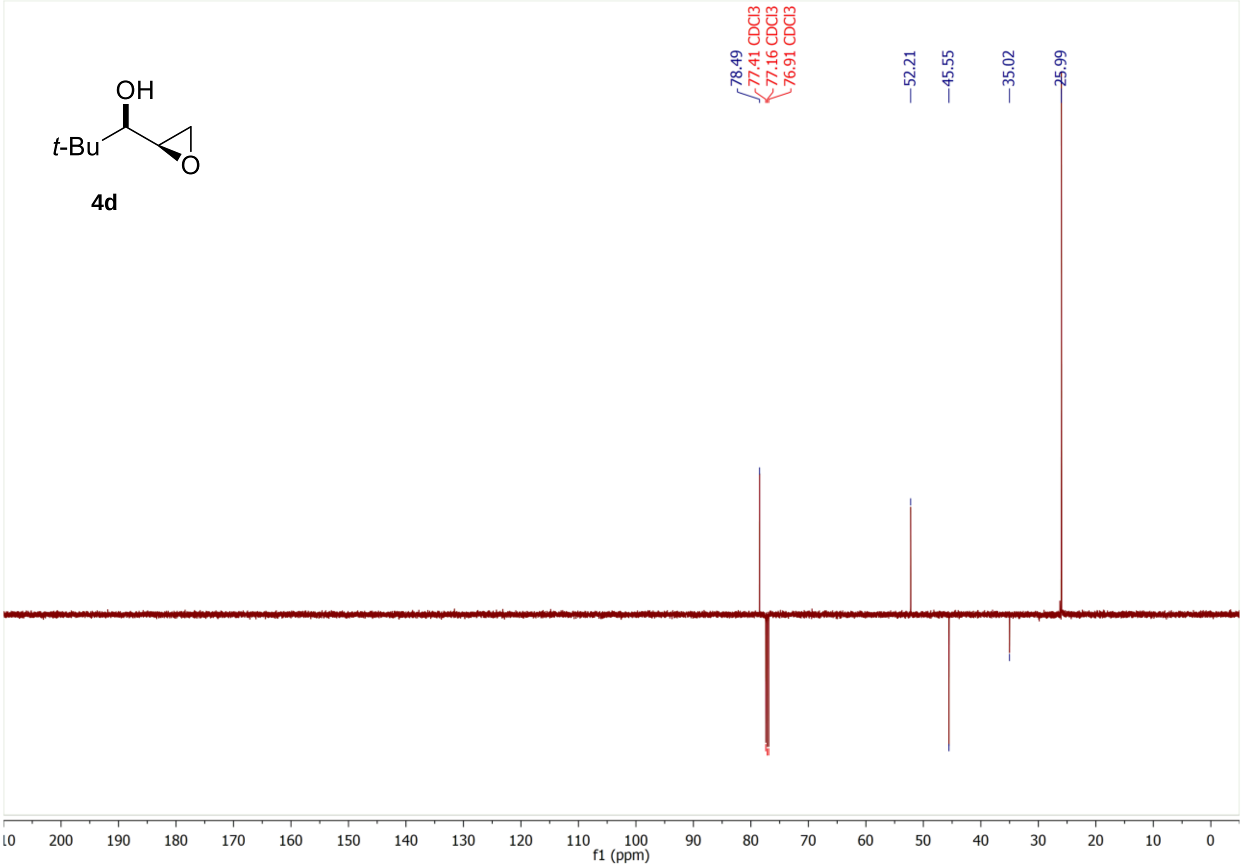

$^{1}$H-NMR (500 MHz) in $CDCl_3$, (*S*)-α-vinylbenzyl alcohol (**3e**) as substrate:

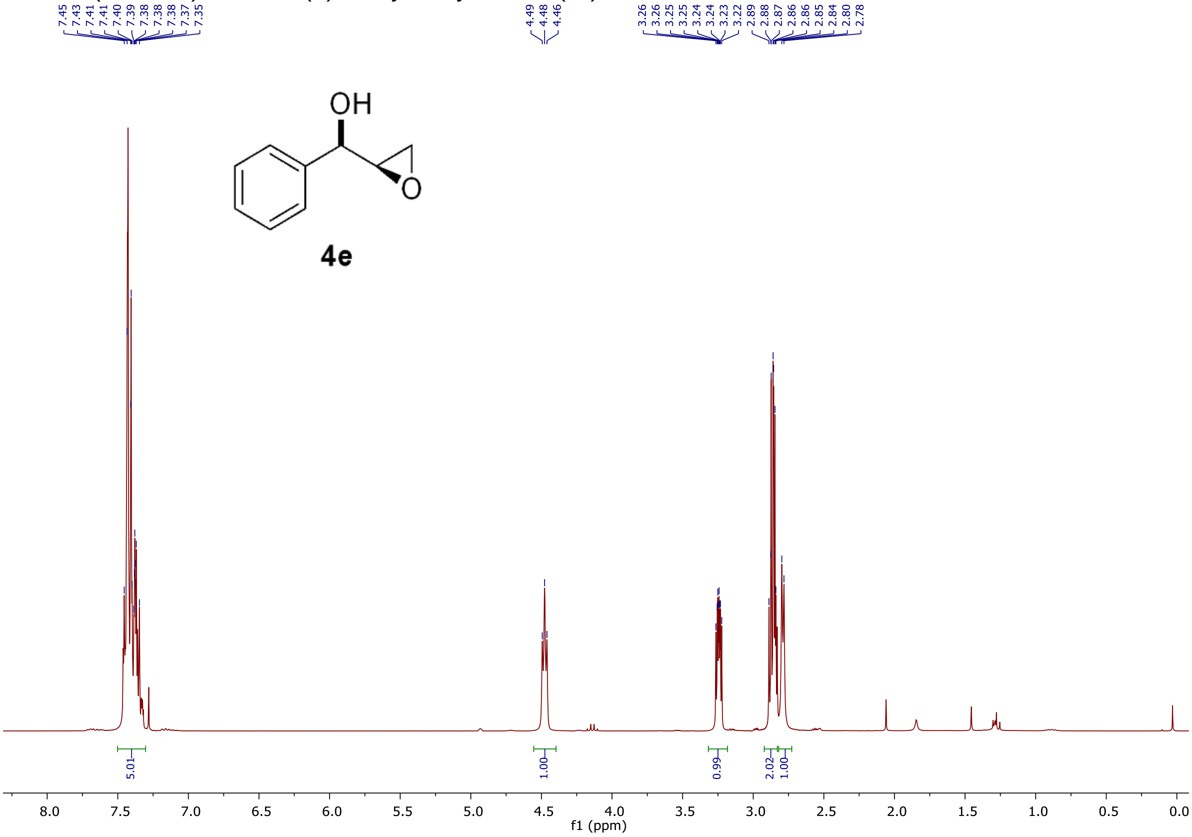


$^{13}$C-NMR (75MHz) in $CDCl_3$, (*S*)-α-vinylbenzyl alcohol (**3e**) as substrate:

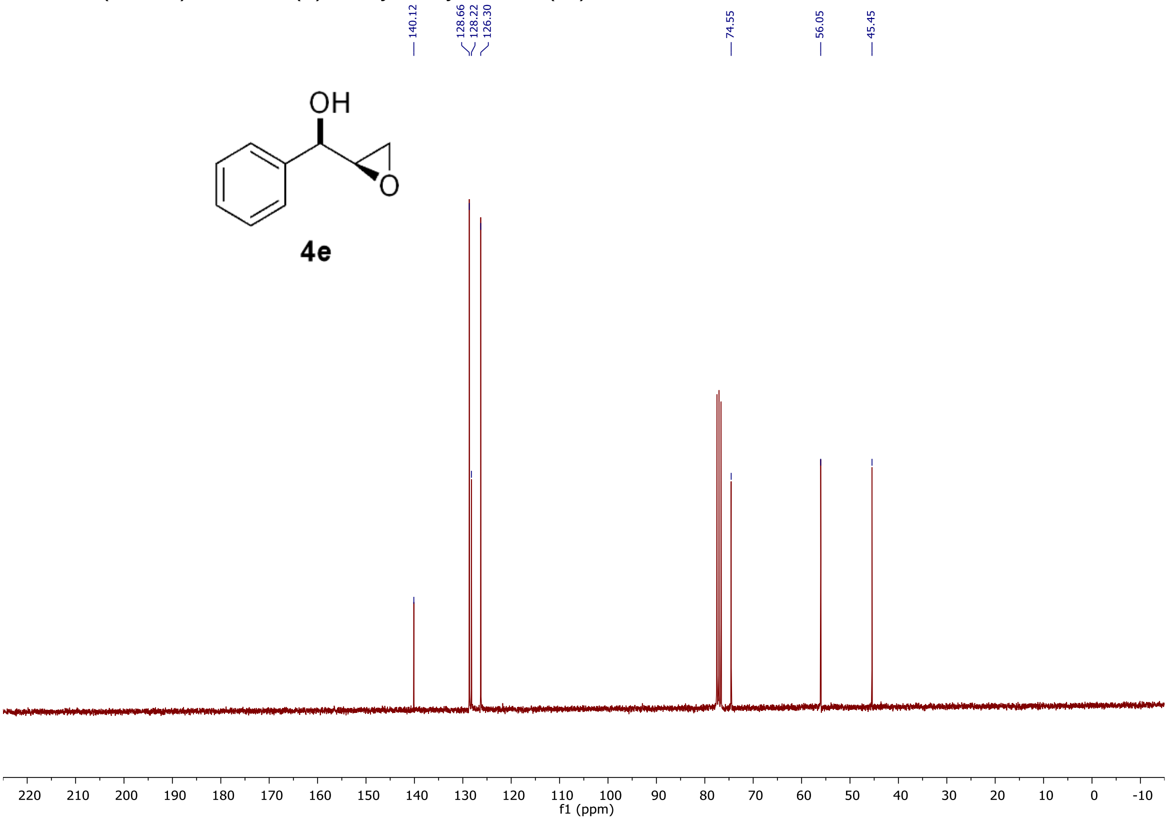

$^1$H-NMR (500 MHz) in $CDCl_3$, (*R*)-1-((*R*)-oxiran-2-yl)tridecan-1-ol (**3f**) as substrate

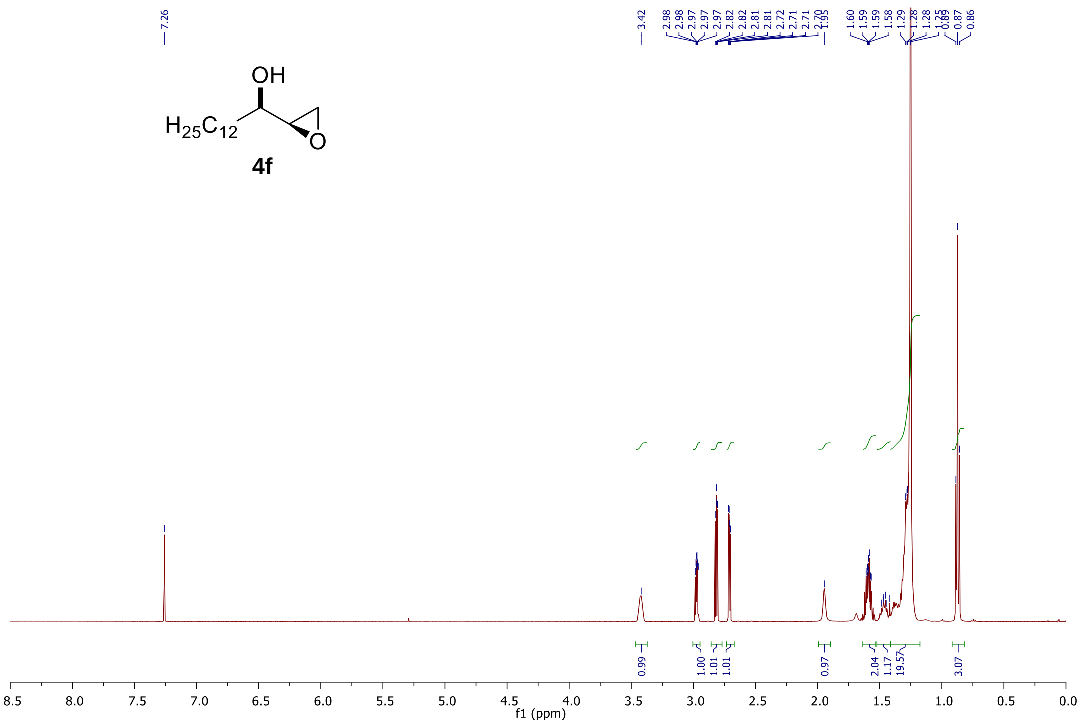


$^{13}$C-NMR (125 MHz) in $CDCl_3$, (*R*)-1-((*R*)-oxiran-2-yl)tridecan-1-ol (**3f**) as substrate

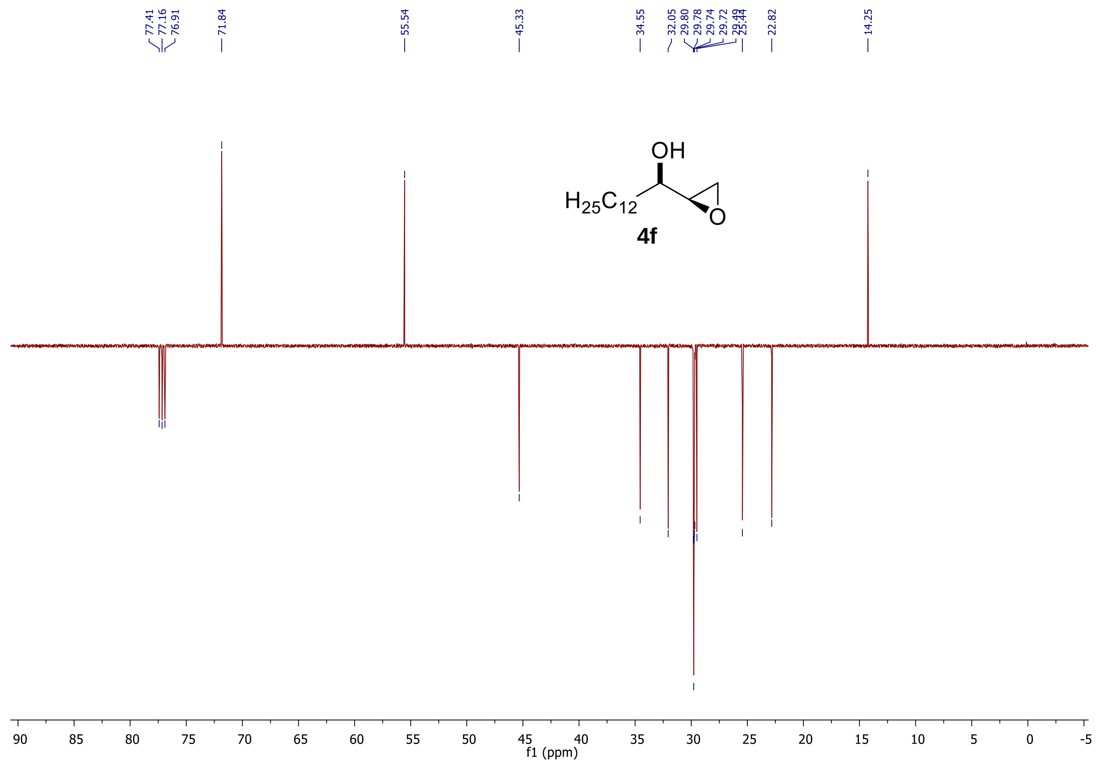

$^1H$-NMR (500 MHz) in $CDCl_3$, *(S)*-(3-methoxyallyl)cyclohexane (**6**) as substrate

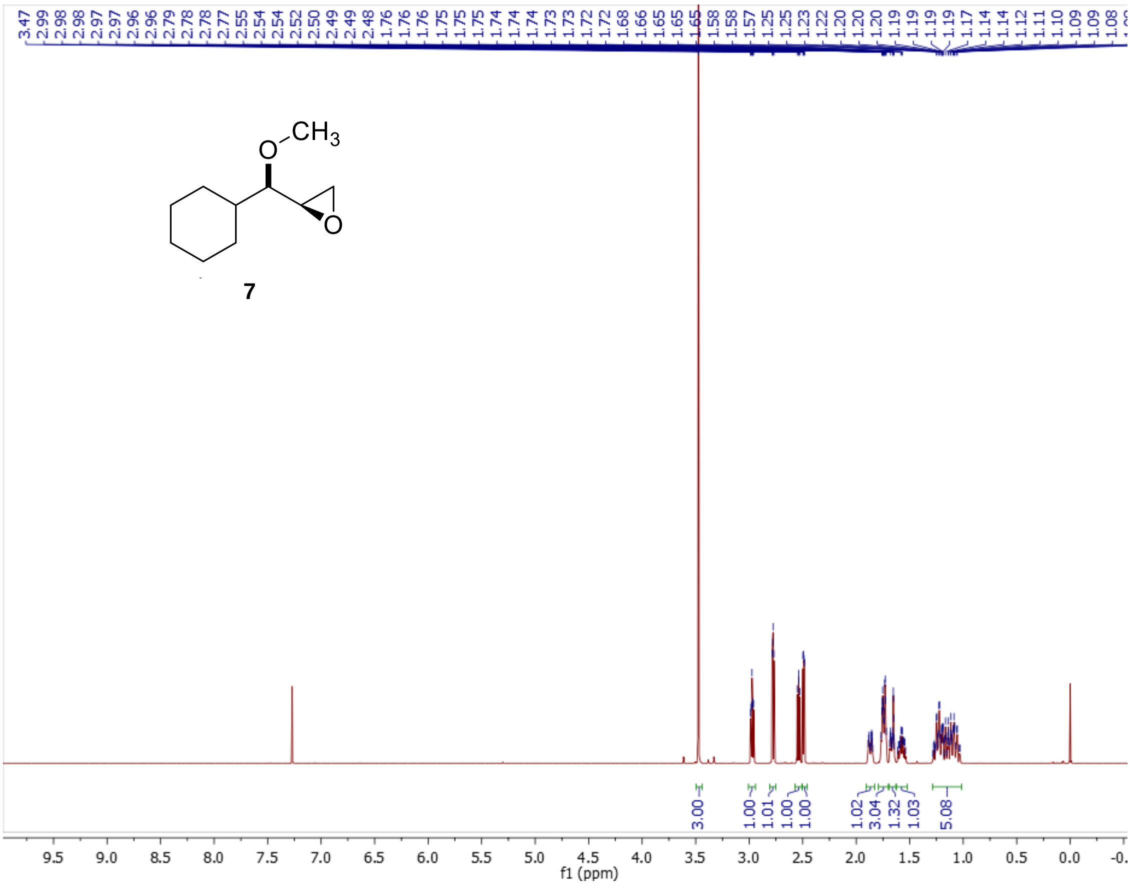


$^{13}C$-NMR (125 MHz) in $CDCl_3$, *(S)*-(3-methoxyallyl)cyclohexane (**6**) as substrate

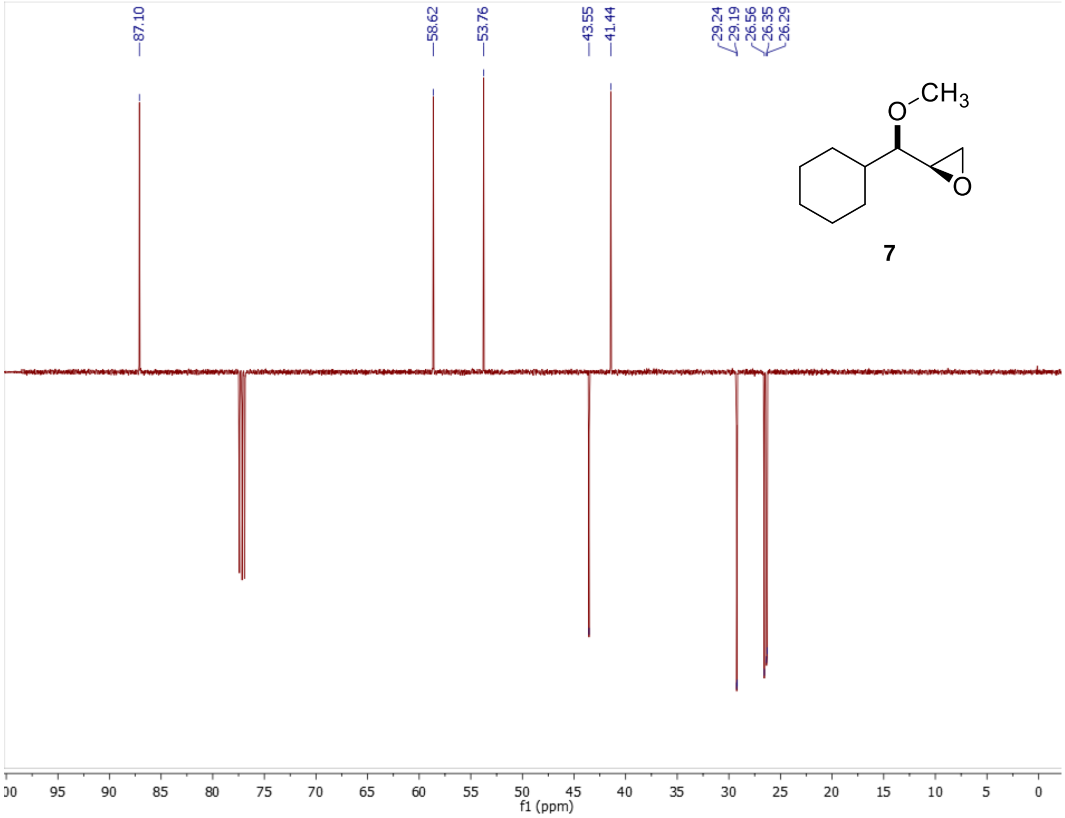

### 4.4. THF Building block 8 and synthetic intermediates 9 and 10

$^{1}$H-NMR (500MHz) of *tert*-butyl(((*R*)-1-((*R*)-oxiran-2-yl)tridecyl)oxy)diphenylsilane (**9**) in $CDCl_3$

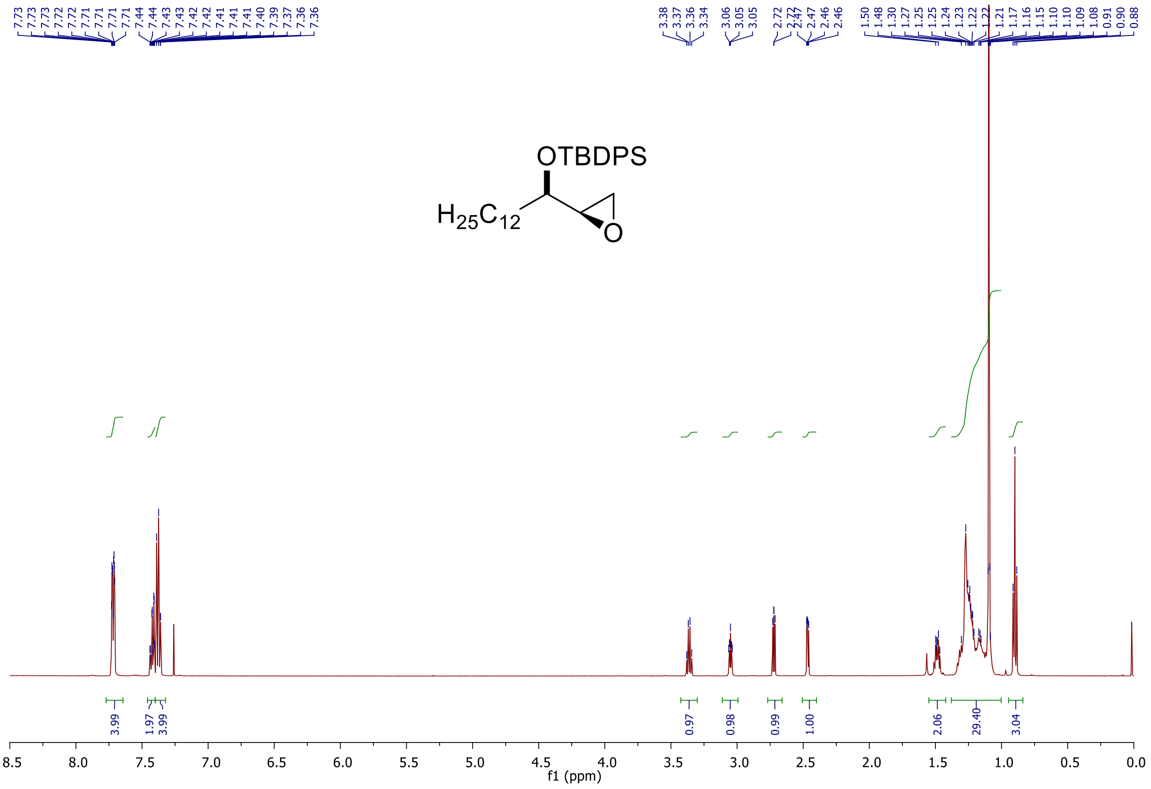

$^{13}$C-NMR (125MHz) of *tert*-butyl(((*R*)-1-((*R*)-oxiran-2-yl)tridecyl)oxy)diphenylsilane (**9**) in $CDCl_3$

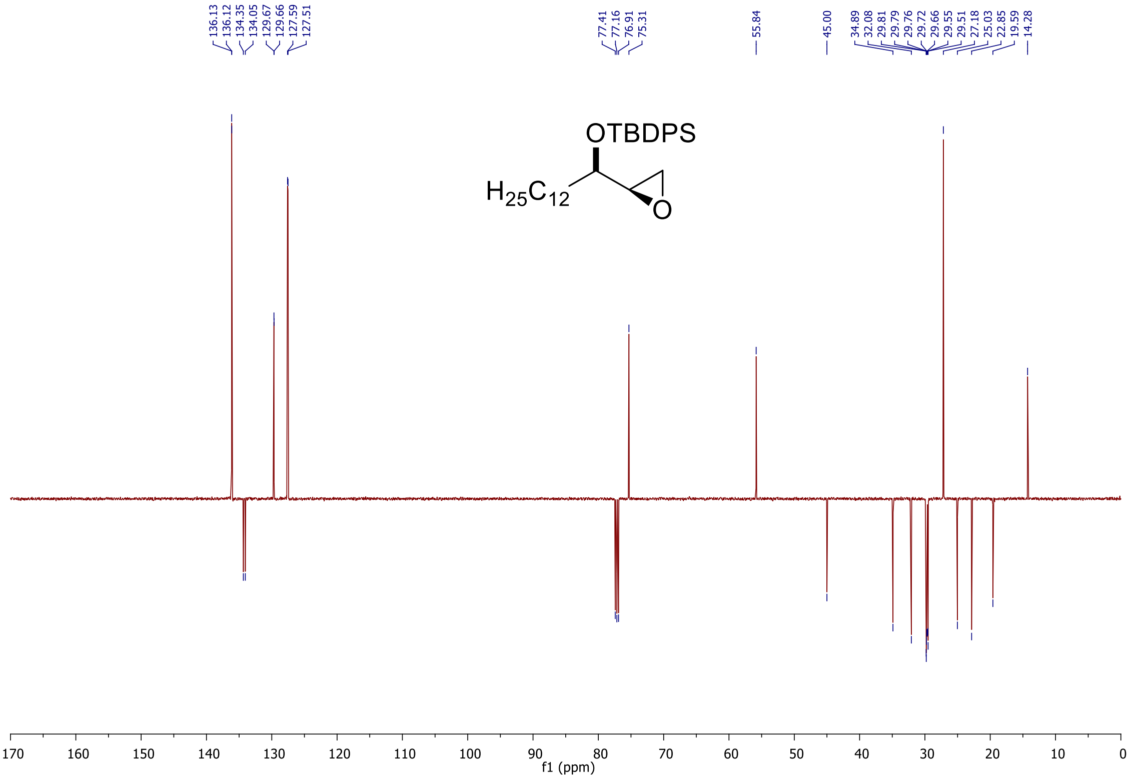

$^{1}H$-NMR (500MHz) of (5*R*,6*R*)-6-((*tert*-butyldiphenylsilyl)oxy)octadec-1-en-5-ol (**10**) in $CDCl_3$

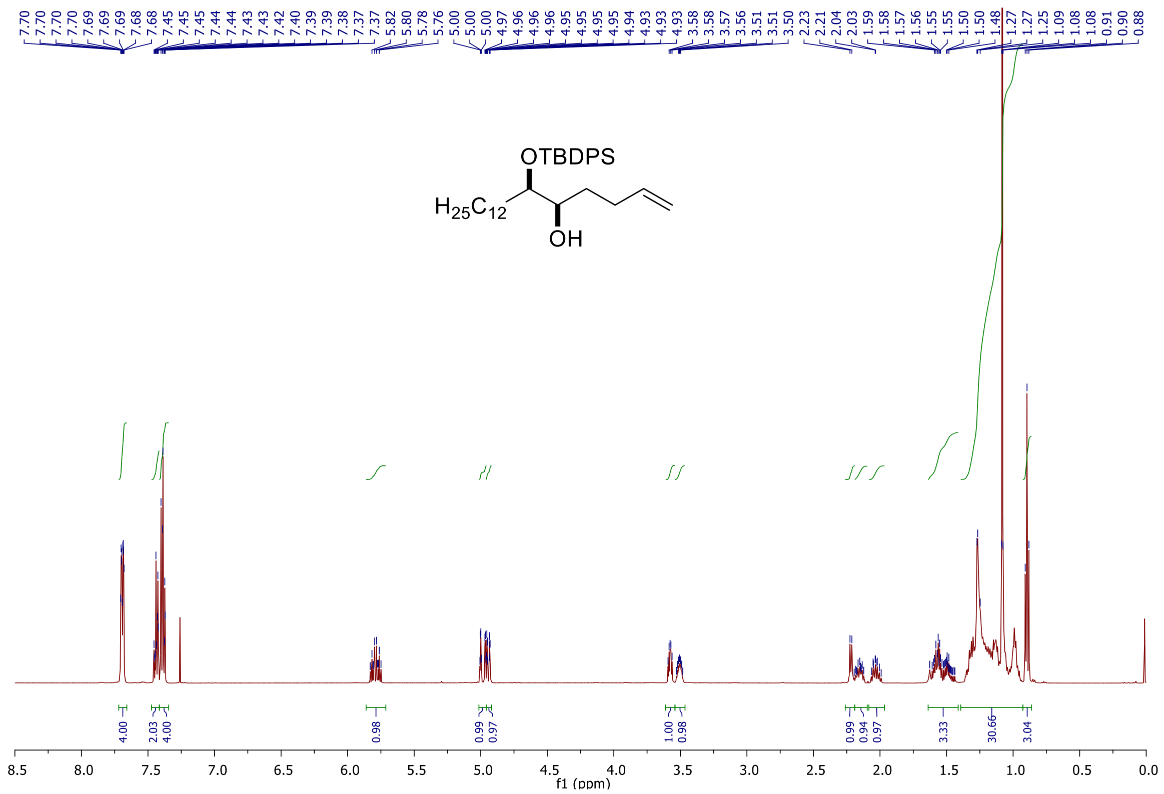

$^{13}C$-NMR (125MHz) of (5*R*,6*R*)-6-((*tert*-butyldiphenylsilyl)oxy)octadec-1-en-5-ol (**10**) in $CDCl_3$

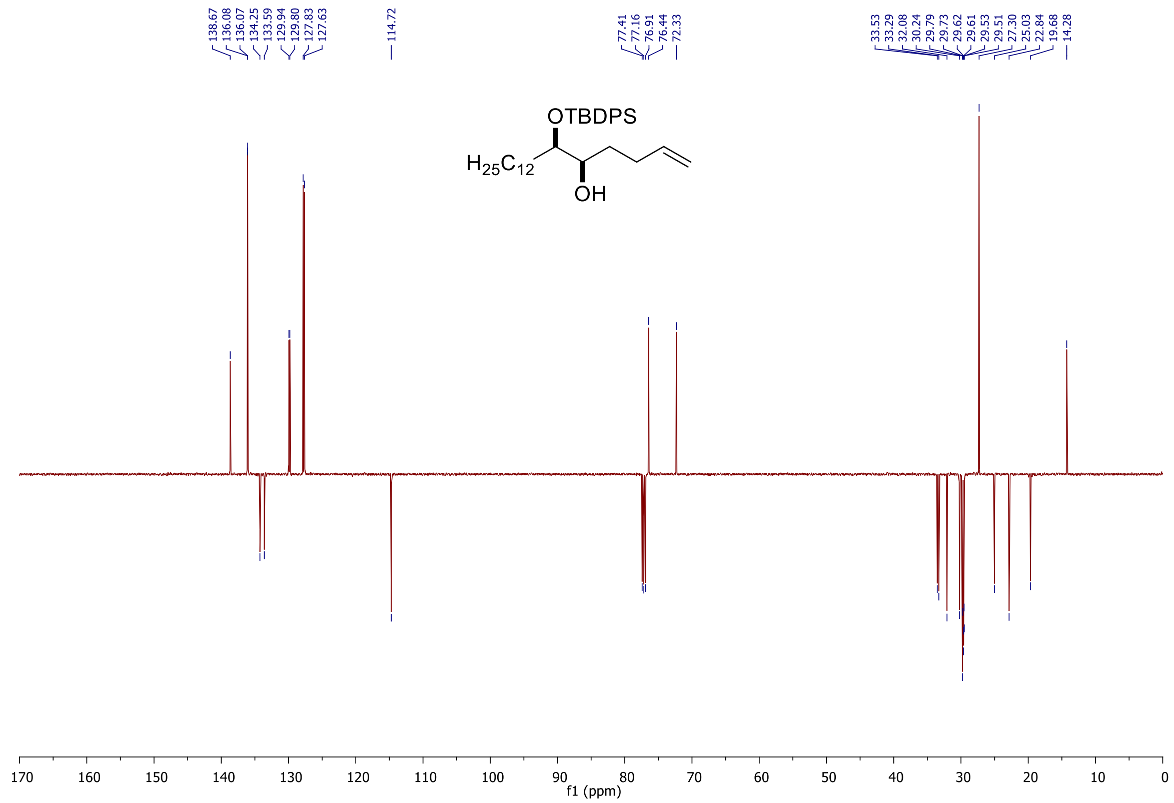

$^{1}$H-NMR (500MHz) of ((2*R*,5*R*)-5-((*R*)-1-((*tert*-butyldiphenylsilyl)oxy)tridecyl)tetrahydrofuran-2-yl)methanol (**8**) in $CDCl_3$

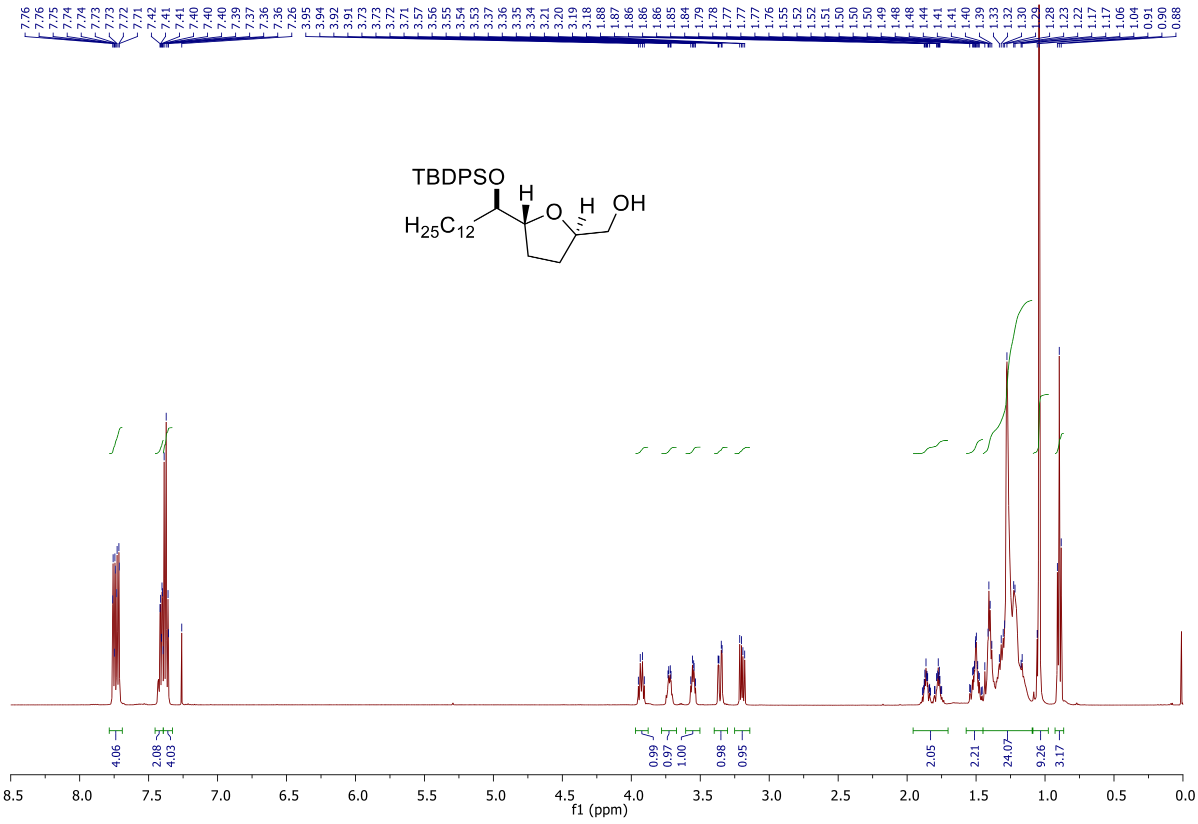

$^{13}$C-NMR (125MHz) of ((2*R*,5*R*)-5-((*R*)-1-((*tert*-butyldiphenylsilyl)oxy)tridecyl)tetrahydrofuran-2-yl)methanol (**8**) in $CDCl_3$

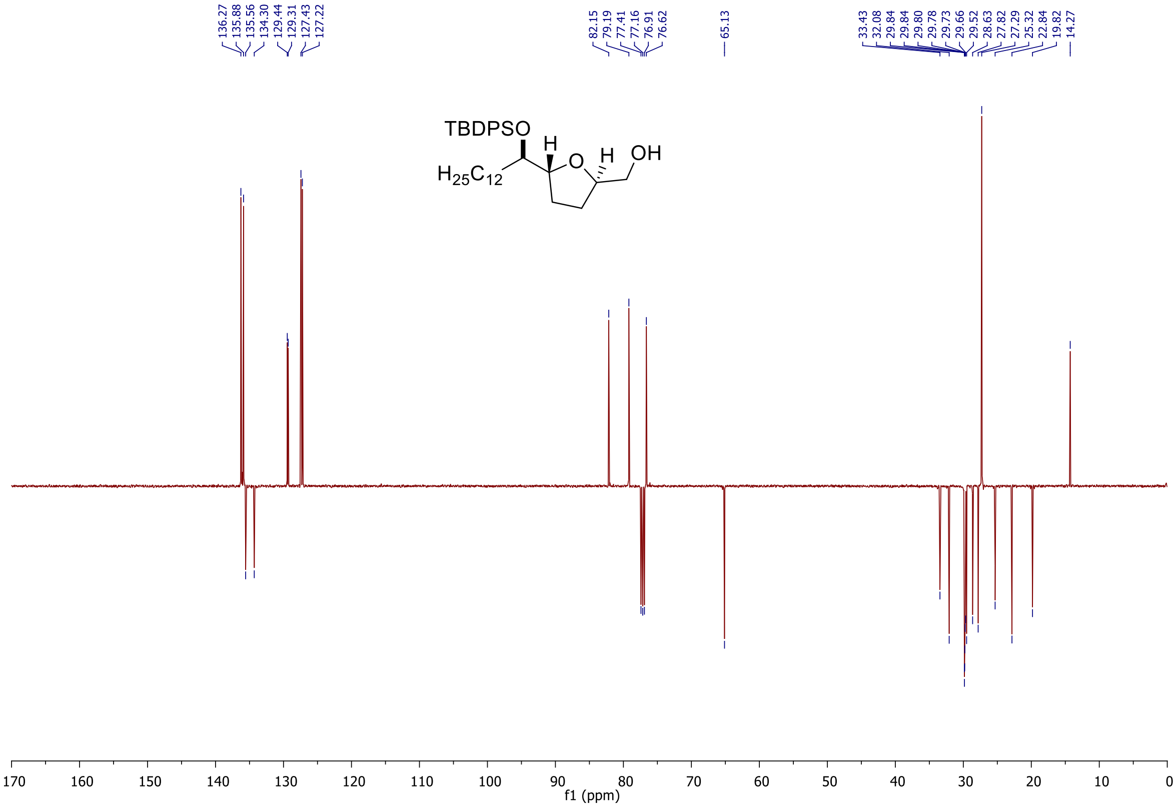

$^{1}$H-NMR (500MHz) monitoring of the epoxidation/ring closure of intermediate **10** to the THF building block **8** ($CDCl_3$).

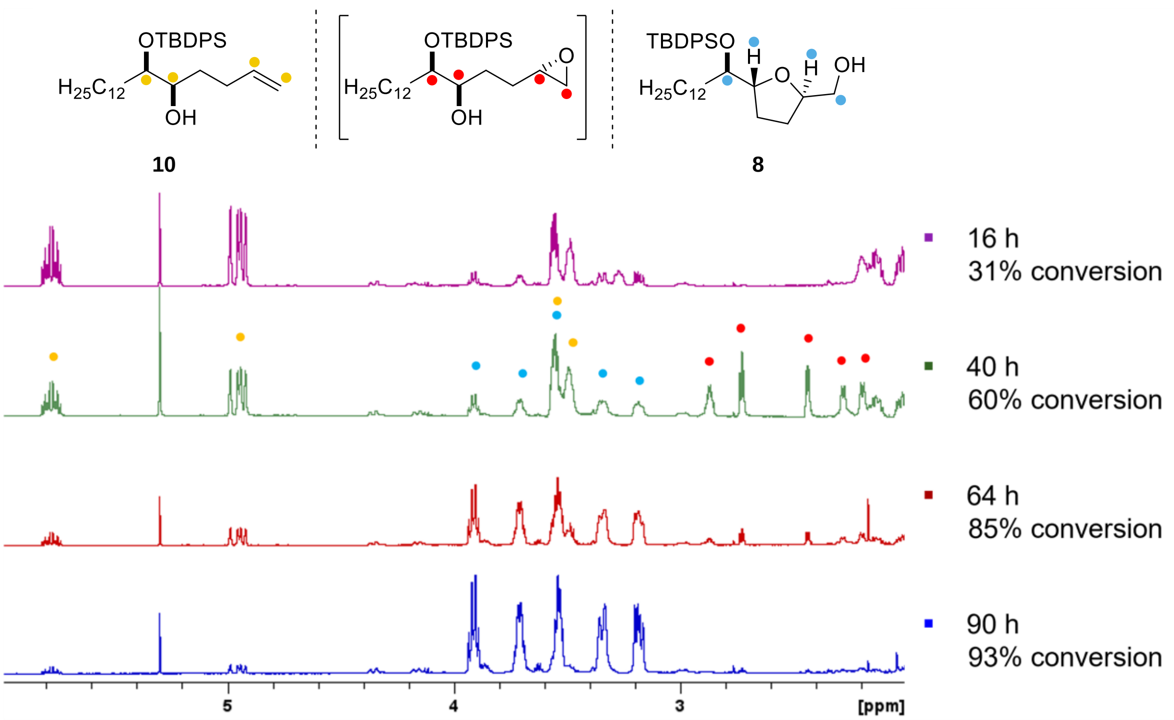

# 5. X-ray Crystallographic Data

## 5.1. X-Ray crystal structure of (*R*,*R*)-cyclohexyl(oxiran-2-yl)methanol (4c)

Crystals suitable for X-ray crystallography were obtained by slow evaporation of a solution in *n*-pentane.

| | |
|---|---|
| CCDC registry number | 2132886 |
| Empirical formula | $C_9H_{16}O_2$ |
| Moiety formula | $C_9H_{16}O_2$ |
| Formula weight | 156.22 |
| Temperature | 150(2) K |
| Wavelength | 1.54178 Å |
| Crystal system | Monoclinic |
| Space group | $P2_1$ |
| Unit cell dimensions | a = 5.2366(2) Å  $\alpha$ = 90 ° |
| | b = 31.8952(10) Å  $\beta$ = 90.3810(10) ° |
| | c = 10.4186(3) Å  $\gamma$ = 90 ° |
| Volume | 1740.10(10) $Å^3$ |
| Z | 8 |
| Density (calculated) | 1.193 $mg/m^3$ |
| Absorption coefficient | 0.656 $mm^{-1}$ |
| F(000) | 688 |
| Crystal size | 2.000 x 0.200 x 0.040 $mm^3$ |
| Theta range for data collection | 4.243 to 72.235°. |
| Index ranges | -5<=h<=6, -39<=k<=39, -12<=l<=12 |
| Reflections collected | 35958 |
| Independent reflections | 6833 [R(int) = 0.0388] |
| Completeness to theta = 67.679° | 99.7 % |
| Absorption correction | Semi-empirical from equivalents |
| Max. and min. transmission | 0.7536 and 0.6502 |
| Refinement method | Full-matrix least-squares on $F^2$ |
| Data / restraints / parameters | 6833 / 1 / 405 |
| Goodness-of-fit on $F^2$ | 1.053 |
| Final R indices [I>2sigma(I)] | R1 = 0.0269, wR2 = 0.0726 |
| R indices (all data) | R1 = 0.0273, wR2 = 0.0728 |
| Absolute structure parameter | 0.00(3) |
| Largest diff. peak and hole | 0.170 and -0.135 e•$Å^{-3}$ |

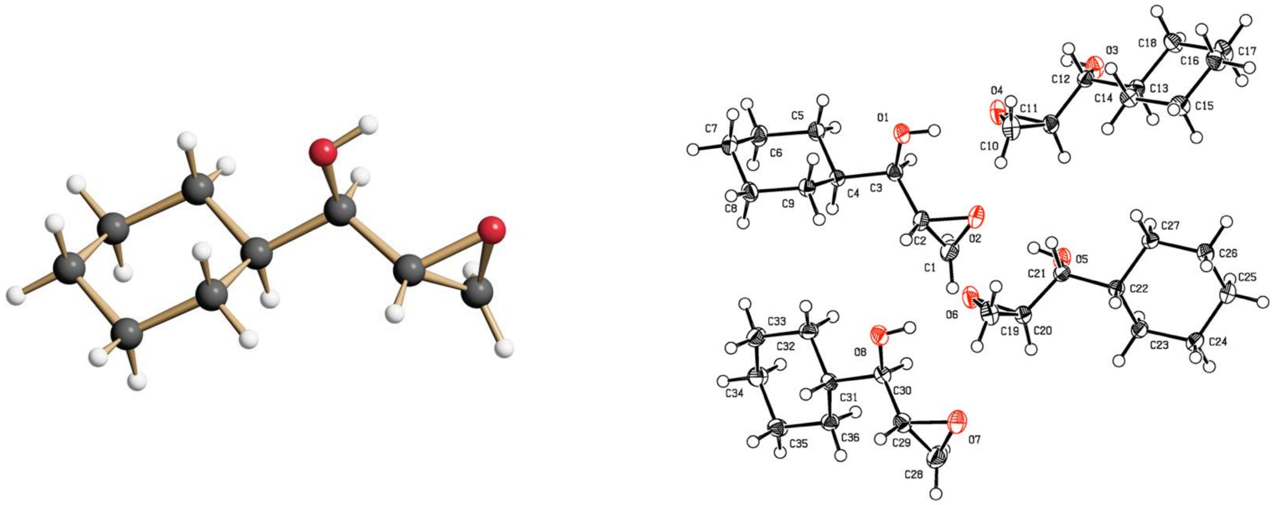

**Figure S1:** Molecular structure (left) and ORTEP (right, four independent molecules in the cell) of the X-ray crystal structure of the epoxy alcohol **4c**. Thermal ellipsoids are drawn at 50% probability level.

### 5.2. X-Ray crystal structure of (*R*,*R*)-1-(oxiran-2-yl)tridecan-1-ol (4f)

Crystals suitable for X-ray crystallography were obtained by slow evaporation of a solution in *n*-pentane.

| | | |
|---|---|---|
| CCDC registry number | 2132887 | |
| Empirical formula | $C_{15}H_{30}O_2$ | |
| Moiety formula | $C_{15}H_{30}O_2$ | |
| Formula weight | 242.39 | |
| Temperature | 100(2) K | |
| Wavelength | 1.54178 Å | |
| Crystal system | Monoclinic | |
| Space group | $P2_1$ | |
| Unit cell dimensions | a = 8.8612(5) Å | $\alpha$ = 90 ° |
| | b = 4.8888(4) Å | $\beta$ = 90.288(4) ° |
| | c = 33.909(2) Å | $\gamma$ = 90 ° |
| Volume | 1468.95(17) $Å^3$ | |
| Z | 4 | |
| Density (calculated) | 1.096 mg/$m^3$ | |
| Absorption coefficient | 0.538 $mm^{-1}$ | |
| F(000) | 544 | |
| Crystal size | 0.500 x 0.100 x 0.020 $mm^3$ | |
| Theta range for data collection | 2.606 to 72.205°. | |
| Index ranges | -10<=h<=10, -5<=k<=6, -41<=l<=41 | |
| Reflections collected | 28259 | |
| Independent reflections | 5640 [R(int) = 0.0658] | |
| Completeness to theta = 67.679° | 99.9 % | |
| Absorption correction | Semi-empirical from equivalents | |
| Max. and min. transmission | 0.7536 and 0.5828 | |
| Refinement method | Full-matrix least-squares on $F^2$ | |
| Data / restraints / parameters | 5640 / 1 / 317 | |
| Goodness-of-fit on $F^2$ | 1.068 | |
| Final R indices [I>2sigma(I)] | R1 = 0.0398, wR2 = 0.1072 | |
| R indices (all data) | R1 = 0.0429, wR2 = 0.1093 | |
| Absolute structure parameter | 0.10(10) | |
| Largest diff. peak and hole | 0.226 and -0.206 e•$Å^{-3}$ | |

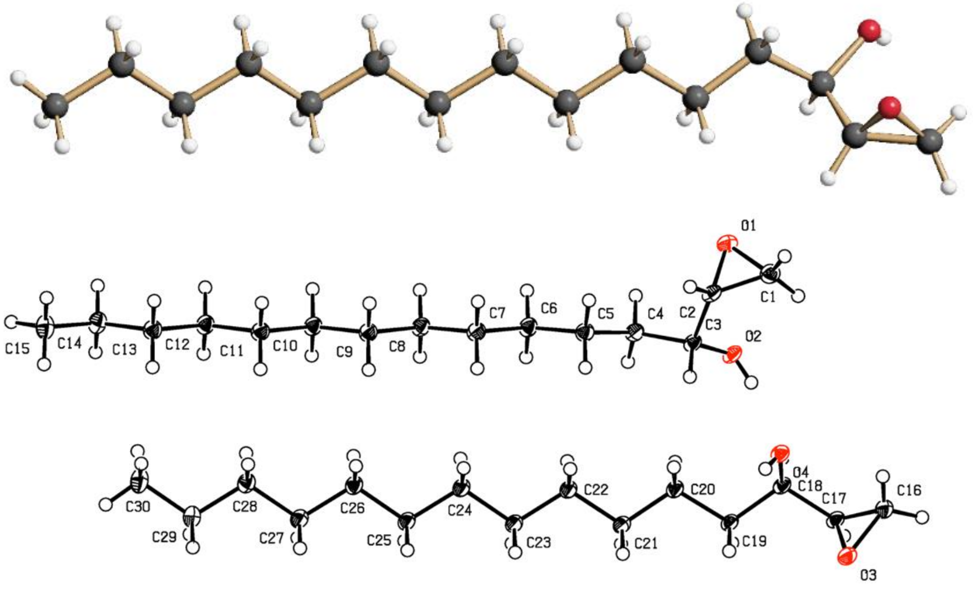


**Figure S2:** Molecular structure (top) and ORTEP (bottom, two independent molecules in the unit cell) of the X-ray crystal structure of the epoxy alcohol **4f**. Thermal ellipsoids are drawn at 50% probability level.

## 6. References

[1] a) H. Engler, M. Lansing, C. P. Gordon, J.-M. Neudörfl, M. Schäfer, N. E. Schlörer, C. Copéret, A. Berkessel, *ACS Catal.* **2021**, *11*, 3206-3217; b) M. Lansing, H. Engler, T. M. Leuther, J.-M. Neudörfl, A. Berkessel, *ChemCatChem* **2016**, *8*, 3706-3709.
[2] C. U. Grünanger, B. Breit, *Angew. Chem.* **2010**, *122*, 979-982; *Angew. Chem. Int. Ed.* **2010**, *49*, 967-970.
[3] R. Doran, P. J. Guiry, *Synthesis* **2014**, *46*, 761-770.
[4] A. Ramdular, K. A. Woerpel, *Org. Lett.* **2020**, *22*, 4113-4117.
[5] a) E. D. Mihelich, *Tetrahedron Lett.* **1979**, *20*, 4729-4732; b) B. E. Rossiter, T. R. Verhoeven, K. B. Sharpless, *Tetrahedron Lett.* **1979**, *20*, 4733-4736.
[6] a) H. V. Ferreira, L. C. Rocha, R. P. Severino, A. L. M. Porto, *Molecules* **2012**, *17*, 8955-8967; b) M. Farrag El-Behairy, E. Sundby, *Tetrahedron: Asymm.* **2013**, *24*, 285-289; c) F. A. Marques, M. A. Oliveira, G. Frensch, B. H. L. N. Sales Maia, A. Barison, C. A. Lenz, P. G. Guerrero Jr. *Lett. Org. Chem.* **2011**, *8*, 696-700.
[7] D. J. Covell, M. C. White, *Angew. Chem.* **2008**, *120*, 6548-6551; *Angew. Chem. Int. Ed.* **2008**, *47*, 6448-6451.
[8] J. Louvel, J. F. S. Carvalho, Z. Yu, M. Soethoudt, E. B. Lenselink, E. Klaasse, J. Brussee, A. P. Ijzerman, *J. Med. Chem.* **2013**, *56*, 9427-9440.
[9] J. H. Choi, Y. K. Choi, Y. H. Kim, E. S. Park, E. J. Kim, M.-J. Kim, J. Park, *J. Org. Chem.* **2004**, *69*, 1972-1977.
[10] M. de Greef, S. Z. Zard, *Org. Lett.* **2007**, *9*, 1773-1776.
[11] P. Van de Weghe, S. Bourg, J. Eustache, *Tetrahedron* **2003**, *59*, 7365-7376.
[12] J. A. Marshall, A. W. Garofalo, *J. Org. Chem.* **1993**, *58*, 3675-3680.
[13] Y. Kobayashi, S. Yoshida, Y. Nakayama, *Eur. J. Org. Chem.* **2001**, *2001*, 1873-1881.
[14] A. Mordini, D. Peruzzi, F. Russo, M. Valacchi, G. Reginato, A. Brandi, *Tetrahedron* **2005**, *61*, 3349-3360.
[15] H. Lin, Y. Tang, S. Dong, R. Lang, H. Chen, *Catal. Sci. Technol.* **2020**, *10*, 2145-2151.
[16] a) P. K. Mandal, G. Maiti, S. C. Roy, *J. Org. Chem.* **1998**, *63*, 2829-2834; b) T. Kawakami, I. Shibata, A. Baba, H. Matsuda, *J. Org. Chem.* **1993**, *58*, 7608-7609; c) N. K. Jobson, A. R. Crawford, D. Dewar, S. L. Pimlott, A. Sutherland *Bioorg. Med. Chem. Lett.* **2009**, *19*, 4996-4998.
[17] B. M. Trost, J. Rey, *Org. Lett.* **2012**, *14*, 5632-5635.
[18] B. Dhotare, A. Chattopadhyay, *Tetrahedron Lett.* **2005**, *46*, 3103-3105.
